# Spontaneous Coronary Artery Dissection: Insights on Rare Genetic Variation From Genome Sequencing

**DOI:** 10.1161/CIRCGEN.120.003030

**Published:** 2020-10-29

**Authors:** Keren J. Carss, Anna A. Baranowska, Javier Armisen, Tom R. Webb, Stephen E. Hamby, Diluka Premawardhana, Abtehale Al-Hussaini, Alice Wood, Quanli Wang, Sri V. V. Deevi, Dimitrios Vitsios, Samuel H. Lewis, Deevia Kotecha, Nabila Bouatia-Naji, Stephanie Hesselson, Siiri E. Iismaa, Ingrid Tarr, Lucy McGrath-Cadell, David W. Muller, Sally L. Dunwoodie, Diane Fatkin, Robert M. Graham, Eleni Giannoulatou, Nilesh J. Samani, Slavé Petrovski, Carolina Haefliger, David Adlam

**Affiliations:** 1 Centre for Genomics Research, Discovery Sciences, BioPharmaceuticals R&D, AstraZeneca (K.J.C., J.A., Q.W., S.V.V.D., D.V., S.H.L., S.P., C.H.).; 2 Department of Cardiovascular Sciences and NIHR Leicester Biomedical Research Centre, University of Leicester, United Kingdom (A.A.B., T.R.W., S.E.H., D.P., A.A.-H., A.W., D.K., N.J.S., D.A.).; 3 Université de Paris, Inserm UMR 970 – Paris, Centre de Recherche Cardiovasculaire, France (N.B.-N).; 4 Victor Chang Cardiac Research Institute, Darlinghurst (S.H., S.E.I., I.T., D.W.M., S.L.D., D.F., R.M.G., E.G.).; 5 St Vincent’s Clinical School, University of NSW Sydney, Kensington (S.E.I., L.M.-C., D.W.M., S.L.D., D.F., R.M.G., E.G.).; 6 Cardiology Department, St Vincent’s Hospital, Darlinghurst, NSW, Australia (D.F.).

**Keywords:** coronary artery dissection, dissection, genetics, spontaneous

## Abstract

Spontaneous coronary artery dissection (SCAD) occurs when an epicardial coronary artery is narrowed or occluded by an intramural hematoma. SCAD mainly affects women and is associated with pregnancy and systemic arteriopathies, particularly fibromuscular dysplasia. Variants in several genes, such as those causing connective tissue disorders, have been implicated; however, the genetic architecture is poorly understood. Here, we aim to better understand the diagnostic yield of rare variant genetic testing among a cohort of SCAD survivors and to identify genes or gene sets that have a significant enrichment of rare variants.

**Methods:**

We sequenced a cohort of 384 SCAD survivors from the United Kingdom, alongside 13 722 UK Biobank controls and a validation cohort of 92 SCAD survivors. We performed a research diagnostic screen for pathogenic variants and exome-wide and gene-set rare variant collapsing analyses.

**Results:**

The majority of patients within both cohorts are female, 29% of the study cohort and 14% validation cohort have a remote arteriopathy. Four cases across the 2 cohorts had a diagnosed connective tissue disorder. We identified pathogenic or likely pathogenic variants in 7 genes (*PKD1*, *COL3A1*, *SMAD3*, *TGFB2*, *LOX*, *MYLK*, and *YY1AP1*) in 14/384 cases in the study cohort and in 1/92 cases in the validation cohort. In our rare variant collapsing analysis, *PKD1* was the highest-ranked gene, and several functionally plausible genes were enriched for rare variants, although no gene achieved study-wide statistical significance. Gene-set enrichment analysis suggested a role for additional genes involved in renal function.

**Conclusions:**

By studying the largest sequenced cohort of SCAD survivors, we demonstrate that, based on current knowledge, only a small proportion have a pathogenic variant that could explain their disease. Our findings strengthen the overlap between SCAD and renal and connective tissue disorders, and we highlight several new genes for future validation.

## Abstract 2

Supplemental Digital Content is available in the text.

Spontaneous coronary artery dissection (SCAD) results from the development of an expanding hematoma within the wall of a coronary artery, caused either by hemorrhage in the tunica media or possibly an intimal tear and resulting in the development of a false lumen.^1^ As the hematoma enlarges, it compresses the true lumen resulting in coronary insufficiency leading to myocardial ischemia, infarction, or both and, in some cases, heart failure and sudden death. Once considered a rare disease, it is now clear that the prevalence has been underestimated^2,3^ and up to 4% of patients with an acute coronary syndrome (ST-segment–elevation myocardial infarct, non–ST-segment–elevation myocardial infarct, or unstable angina) who undergo coronary angiography present with SCAD.^4^

SCAD mainly affects young to middle-aged women without an increase in typical cardiovascular risk factors and accounts for up to half of pregnancy-associated myocardial infarction. SCAD has also been associated with multiparity, systemic arteriopathies (particularly fibromuscular dysplasia [FMD]), connective tissue disorders (CTD), inflammatory diseases, and polycystic kidney disease (PCKD), and associated precipitating factors include intensive exercise or emotional stress.^2,3^

The cause of SCAD is believed to include a genetic component, although this remains poorly understood. There have been reports of familial SCAD risk^5^ and 5% to 8% of SCAD patients have been found to carry deleterious variants in genes that cause heritable CTDs, including *FBN1*, *COL3A1*, and *SMAD3*.^6–8^ Of less clear significance, variants in *PKD1*, associated with autosomal dominant PCKD, and *LMX1B*, associated with Nail-patella syndrome, have also been described in SCAD.^7^ More recently, variants in *TSR1* and *TLN1* have been reported in familial and sporadic SCAD cases.^9,10^ Additionally, common variants including in *PHACTR1/EDN1* have been associated with SCAD,^11^ highlighting the genetic and etiological heterogeneity of the condition.

Sequencing enables the examination of rare variation to better understand its contribution to the genetic architecture of SCAD. The objectives of this study were to leverage genome sequencing data generated on SCAD survivors and exome-sequence data from appropriate UK biobank controls to (1) better understand the diagnostic yield of rare variants in the largest sequenced cohorts of SCAD survivors, (2) gain insight into the genetic architecture of SCAD by identifying genes or gene sets with an excess of rare variants in the case/control populations, and (3) identify biologically plausible genes that could be candidates for further validation.

## Methods

Methods are provided in Material in the Data Supplement. The data that support the findings of this study are available from the corresponding author upon reasonable request. The UK SCAD cohort was approved by the UK National Research Ethics Service (14/EM/0056) and the UK Health Research Authority and conducted in accordance with the Declaration of Helsinki. All patients provided signed informed consent before the study start. The Victor Chang Cardiac Research Institute SCAD cohort was approved by the St Vincent’s Human Research Ethics Committee (2019/ETH03171) and conducted in accordance with the National Health and Medical Research Council’s National Statement on Ethical Conduct in Human Research and the Committee for Proprietary Medicinal Products/International Conference on Harmonization Note for Guidance on Good Clinical Practice. All patients provided informed consent before the start of the study. Controls for association analyses were selected from UK Biobank participants after screening for cardiovascular disease.

## Results

### Patient Cohorts

The SCAD survivors sequenced for this study consists of 384 patients from the UK SCAD registry and 92 patients from the Victor Chang Cardiac Research Institute SCAD cohort. All patients had angiographically confirmed diagnosis of SCAD. The clinical characteristics of the SCAD patients are described in Table 1. The majority of patients are females of European ancestry with a single SCAD event. Remote arteriopathy, including dilations, dissections, aneurysms, and fibromuscular dysplasia, in another vascular bed were present in 29.17% of the UK SCAD cohort and 14% of the Victor Chang Cardiac Research Institute SCAD cohort have a remote arteriopathy, including dilations, dissections, aneurysms, and fibromuscular dysplasia, in another vascular bed. Only 3 individuals in the UK SCAD cohort and one patient in the Victor Chang Cardiac Research Institute SCAD cohort have a CTD.

**Table 1.**
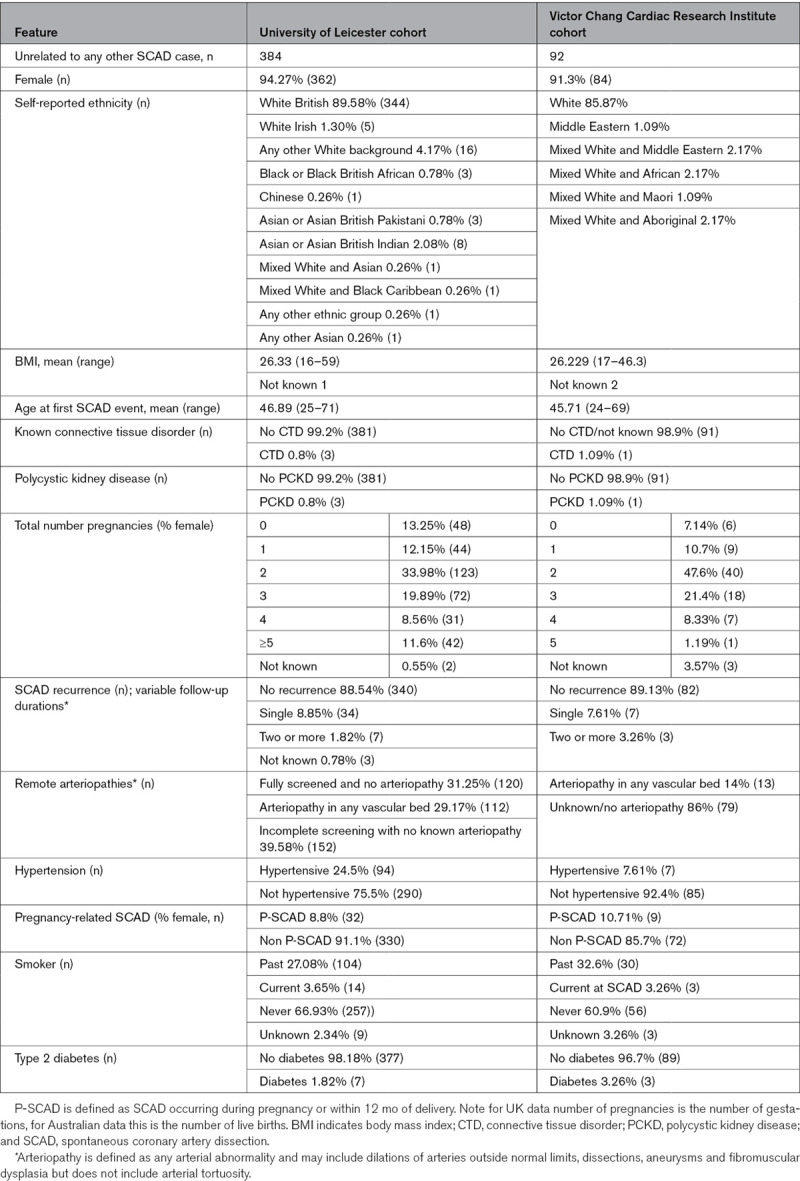
SCAD Patient Characteristics

### Pathogenic Variants Were Identified in 3.6% of SCAD Patients in the UK Cohort

We assessed genes previously reported in SCAD patients or related conditions (tier 1 and tier 2 gene lists as described in Methods and Table I in the Data Supplement) and identified 15 different pathogenic or likely pathogenic variants according to American College of Medical Genetics guidelines^12^ in 14/384 (3.6%) cases (Table 2). At least 99% of the SCAD cases have read depth ≥10X for ≥98% of the consensus coding sequence of the 6 tier 1 genes (*COL3A1*, *FBN1*, *PKD1*, *SMAD3*, *TLN1*, and *TSR1*; Figure I in the Data Supplement), suggesting adequate coverage to detect protein-coding single nucleotide variants and indels in these genes. Nine individuals had variants in tier 1 genes (*COL3A1* n=2, *PKD1* n=5, and *SMAD3* n=2) and 5 individuals with variants in tier 2 genes (*LOX* n=1, *MYLK* n=1, *TGFB2* n=2, and *YY1AP1* n=1). One of our SCAD cases has 2 heterozygous variants in *YY1AP1*. Homozygous or compound heterozygous protein-truncating variants (PTVs) in *YY1AP1* can cause Grange syndrome, which is characterized by severe, early-onset vaso-occlusive disease and FMD-like vascular features, brachydactyly, syndactyly, fragile bones, and learning disabilities^14^; however, we have been unable to phase the 2 heterozygous *YY1AP1* PTVs in the patient. Of the 15 different pathogenic/likely pathogenic alleles, 11 (73%) have previously been reported in SCAD or a related condition in HGMD or ClinVar, and 4 (2 in *PKD1* and 2 in the single *YY1AP1* case) are novel PTVs reported here for the first time.

**Table 2.**
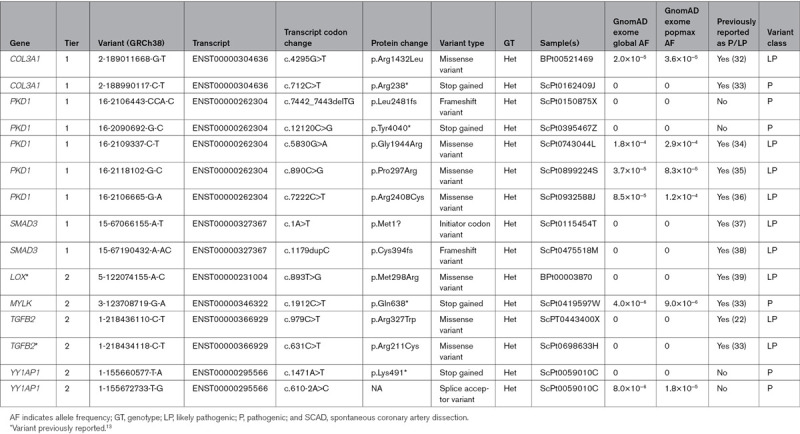
Pathogenic and Likely Pathogenic Variants Identified in 384 SCAD Cases

There were an additional 19 cases where a single heterozygous variant was identified in a recessive tier 1 or 2 genes, including 3 cases with structural variants (SVs; Figure II in the Data Supplement). These were not considered pathogenic or likely pathogenic because they were not identified in biallelic form (Table II in the Data Supplement).

We did not identify any pathogenic or likely pathogenic SVs. However, we did identify a heterozygous deletion (GRCh38.10:52048268-52058807del; 11 kb) in ScPt0668423L that causes an in-frame deletion of exon 6 of *PRKG1*. The deletion is absent in gnomAD v2 SVs, Decipher, and ClinVar, and a clear drop in coverage is visible on examination of the reads (Figure II in the Data Supplement).

We sought to further investigate the 7 genes in which we identified pathogenic/likely pathogenic variants in the UK cohort in an independent Victor Chang Cardiac Research Institute cohort of 92 sporadic SCAD cases. Among these 7 genes, we identified a single putatively pathogenic PTV in *COL3A1* (2-189004115-A-AG, ENST00000304636: c.2798dupG [p.Ser934fs]).

### Clinical Features of SCAD Patients With Identified Pathogenic Variants

Clinical details of the 14 cases carrying pathogenic or likely pathogenic variants are provided in Table III in the Data Supplement. Two (ScPt0150875X and ScPt0395467Z) of the 5 cases with *PKD1* variants, both PTVs, have PCKD. The mother of ScPt0395467Z has PCKD, and hypermobility and their maternal grandmother had PCKD and died due to ruptured berry aneurysm. ScPt0150875X has no family history of PCKD, cardiovascular, other than hypertension and hypercholesterolemia, or CTDs. One case (ScPt0743044L) with a likely pathogenic missense variant in *PKD1* has hypermobility with Ehlers-Danlos syndrome-like features, reports easy bruising and has a family history of SCAD (first cousin) and hypermobility (son). Of the remaining 2 patients, one (ScPt0899224S) has an aortic root diameter at the upper limit of normal, neither patient has any other SCAD-associated phenotype or relevant family history. Notably, one other participant in the UK SCAD cohort and one patient in the Victor Chang Cardiac Research Institute SCAD cohort have PCKD and missense variants of uncertain significance in *PKD1* (16-2100038-A-G, ENST00000262304: c.9746T>C [p.Leu3249Pro] and 16-2103514-A-G, ENST00000262304: c.8543T>C [p.Val2848Ala], respectively).

Neither case (BPt00521469 and ScPt0162409J) with *COL3A1* variants has typical characteristics of vascular Ehlers-Danlos syndrome. BPt00521469 has high palate and pes planus and has one male sibling with patella dislocation and another with recurrent pneumothorax. ScPt0162409J has subconjunctival hemorrhage, and both she and her mother have scoliosis.

One patient (ScPT0443400X) with a *TGFB2* variant, which is mutated in Loeys-Dietz syndrome (LDS), has hypermobility, Chiari malformation (mother and sister also affected) and reports easily bruising. The other case (ScPt0698633H) with likely pathogenic *TGFB2* variant has remote arteriopathies including left carotid artery dissection and right internal carotid aneurysm, and no family history of disease. The 2 patients with *SMAD3* variants, which also causes LDS) have not yet been screened for remote arteriopathies. Both have a family history of aortic aneurysm.

Our patient with a *LOX* variant also had right internal carotid dissection and FMD and their mother died due to intracerebral bleed secondary to aneurysm. The patient with a pathogenic variant in *MYLK* has dyslipidemia and systemic inflammatory disease and no relevant family history. The case with 2 *YY1AP1* variants has FMD, renal artery stenosis, brachydactyly, and migraines.

We could identify no significant differences between the 14 cases with pathogenic or likely pathogenic variants and the remainder of cases in terms of their age, recurrence, and several other clinical end points including remote arteriopathies and hypermobility (Table IV in the Data Supplement).

The patient with a deletion of *PRKG1* exon 6 had no other notable clinical characteristics besides a single SCAD event.

### Gene-Level Collapsing Analysis

To identify genes enriched for rare variants in SCAD cases in the UK cohort compared with controls, we used gene-level collapsing analysis.^15–17^ Cases are the subset who are of European ancestry, are unrelated, and pass quality control filters (n=357). For controls, we used exome-sequencing data from 13 722 individuals from the UK Biobank who had high-quality exome-sequencing data, were unrelated, of European ancestry, and had no report of a relevant disease (Table V in the Data Supplement). We ran 11 different collapsing analysis models with different definitions of qualifying variants (QVs), each designed to capture slightly different genetic architecture (Methods in the Data Supplement and Table 3).

**Table 3.**
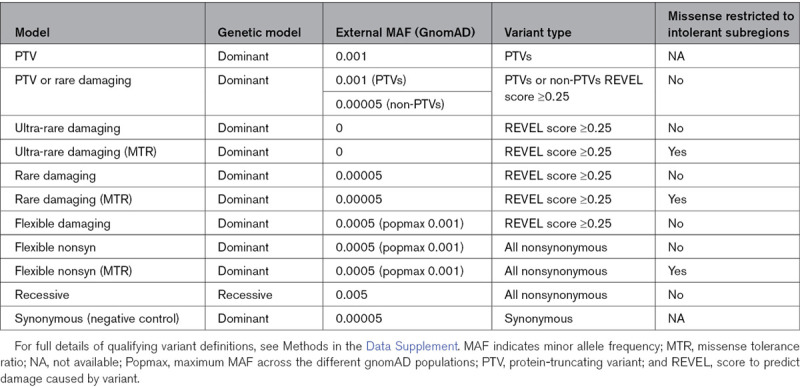
Eleven Different Genetic Models Used to Define Qualifying Variants for Collapsing Analysis

No association reached study-wide significance (Table 4, Tables VI and Figure III in the Data Supplement). One of the highest-ranked associations was *PKD1*, which was the highest-ranked gene in the ultra-rare damaging missense tolerance ratio model (*P*=7.3×10^−6^). The association for the ultra-rare damaging model is weaker (*P*=0.0018), demonstrating that variants in regions of *PKD1* that are intolerant to missense variation are more likely to be associated with SCAD (Table VII in the Data Supplement). The *PKD1* signal could be considered significant upon restricting the search-space and thus multiple-testing correction to the top decile (n=1928) highest expressed genes in the coronary artery tissue data from the GTEX database (accessed November 19, 2019).^18^

**Table 4.**
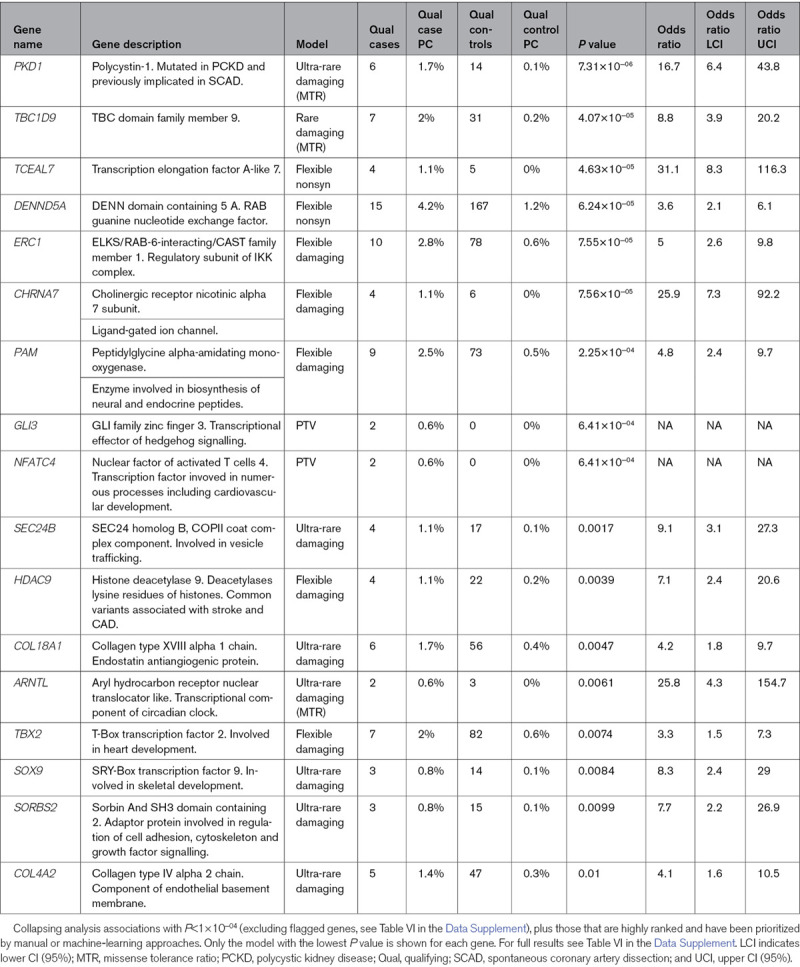
Selected Highly Ranked Collapsing Analysis Results

### Prioritization of Nonsignificant Genes From Collapsing Analysis Results by Manual Review and Automated Machine Learning

Although no association reached study-wide significance, we hypothesized that within the highly ranked results may be genes in which rare variants do increase the risk of SCAD, but our current study is underpowered to highlight them. Therefore, we further investigated highly ranked results with the aim of identifying a shortlist of genes not previously associated with SCAD but are functionally plausible and could be further investigated in future larger SCAD studies. We used 2 complementary approaches: manual review alongside an automated machine-learning approach. Genes prioritized by the manual approach (which was conducted blind to the results of the automated machine-learning method) include *PAM, GLI3, SEC24B, COL18A1, NFATC4, ARNTL, TBX2, HDAC9, SOX9, SORBS2*, and *COL4A2*; all implicated in blood pressure regulation and cardiovascular system development and morphology (Table VIII in the Data Supplement).

Although the manual approach is flexible and thorough, it requires substantial expertise in the phenotype and can be laborious. Thus, we also used mantis-ml,^19^ a machine-learning method for gene prioritization. We trained mantis-ml on SCAD tier 1 and 2 genes to identify which of the remaining genes share characteristics most commonly with those genes. During the application on SCAD tier 1 and 2 genes, mantis-ml predictions were primarily driven by disease/phenotype-specific mouse knockout models, protein-protein interactions with known SCAD-associated genes, gene expression in heart and aorta, GWAS hits and heart-associated Gene Ontology terms (Figure IV in the Data Supplement).

To assess whether the top-ranked genes from the collapsing analysis were preferentially enriched for the top mantis-ml predictions, we performed multiple hypergeometric tests between the top 5% mantis-ml predictions and the top gene hits from collapsing analysis (*P*<0.05) for different types of QVs. We observed that the top-ranked genes from the ultrarare variant collapsing analysis were significantly enriched for the top 5% mantis-ml predictions (Figure VA in the Data Supplement). There was no significant enrichment when adopting the synonymous variant collapsing analysis model (Figure VB in the Data Supplement), suggesting that mantis-ml’s predictions are likely pointing towards the top-ranked genes from collapsing analyses that are likely to be SCAD risk genes.

Mantis-ml yielded a consensus list of 10 genes that are highly ranked in the collapsing analysis for ultra-rare variants (Table 4 and Figure VC in the Data Supplement). All 10 consensus predictions from mantis-ml were also prioritized by the manual approach.

### Gene-Set Enrichment Analysis

We next explored gene-set enrichment among 9339 predefined gene sets, including our SCAD gene lists. Only the SCAD tier 1 gene set (*P*=3.6×10^−7^, comprising 6 genes) reached the Bonferroni-corrected significance threshold of *P*<5.4×10^−7^; a key positive control demonstrating that there is clear statistical enrichment of damaging variants in tier 1 genes among SCAD cases compared with controls. This signal is driven by 6 individuals with QVs in *PKD1*, 3 in *COL3A1*, and 2 in *SMAD3*. The gene set with the second lowest *P* value is Loop of Henle development genes (*P*=2.4×10^−6^, comprising 11 genes), driven by the same 6 individuals with QVs in *PKD1*, 2 in *HES5*, 2 in *UMOD*, and one in *DLL1* (Tables VII and IX and Figure VI in the Data Supplement). Importantly, for these 2 gene sets, upon excluding individuals with *PKD1* QVs, both remained highly ranked albeit no longer significant (for SCAD tier 1 genes *P*=0.016 [122/9339 gene sets] and for Loop of Henle development genes *P*=0.04 [268/9339 gene sets]). Thus, while the signals observed in these gene sets are clearly primarily driven by *PKD1*, there appear to be suggestive signal from the remaining genes in the gene sets.

## Discussion

This study is the largest analysis to date of Mendelian-like rare genetic variants that might be responsible for SCAD. We have assessed the entire coding genome, rather than solely applying a candidate approach and have investigated the contribution of rare genetic variants to SCAD by analyzing genomes of a large cohort of SCAD survivors for pathogenic variants and performing rare variant collapsing analyses. We identified variants deemed pathogenic or likely pathogenic for CTDs and PCKD, including in genes not previously reported in SCAD. Our findings strengthen the evidence that SCAD is an occasional clinical outcome in these conditions with implications for both clinical and genetic screening of SCAD patients. We also identified several new genes enriched for rare variants that require validation in larger future studies.

Overall, we identified variants that might be responsible for SCAD in 14/384 (3.6%) cases in the study cohort, which is in line with expectation from smaller studies.^6,7^ Importantly, for controls, we used exome-sequencing data from the UK Biobank. The size of this cohort along with available phenotypic data allowed us to apply strict selection criteria, providing a major advantage over the controls of convenience used by many previous rare variant studies. As such, our identification of pathogenic or likely pathogenic variants probably represents an accurate reflection of the genetic burden of rare variants in SCAD and provides a better understanding of the genetic component of disease.

These findings have important implications for patient management. The role of clinical genetic testing in SCAD survivors has been uncertain. Our results support the hypothesis that rare variants are likely causal in only a small subpopulation of SCAD cases, some with clinical features or a family history of CTDs or PCKD, suggesting that although these represent an important and pathophysiologically informative group, the yield from routine clinical genetic screening based on our current knowledge of SCAD genetics would be low. The combination of careful clinical phenotyping (including assessment for typical changes in the palate, skin, and facial features as well as musculoskeletal abnormalities^20,21^), remote arteriopathy cross-sectional imaging from brain to pelvis (which will necessarily include renal imaging) and assessment of family history, to include CTDs and PCKD, will identify most patients with pathogenic variants for further genetic assessment. Our data suggest a small number of patients with pathogenic variants will still be missed by this approach. However, given the rarity of such patients, the psychological morbidity of genetic screening and the lack of genotype-specific effective medical interventions, the merit of routine genetic screening of all SCAD survivors is debatable.

Five of our SCAD patients had pathogenic or likely pathogenic in *PKD1*, and *PKD1* was also one of the highest-ranked associations from the collapsing analysis. Two additional SCAD patients were also noted with PCKD and *PKD1* missense variants of uncertain significance. Pathogenic *PKD1* variants have previously been reported in SCAD patients,^7,22^ and the observation here highlights that the co-occurrence of SCAD and *PKD1* dysfunction is moving beyond being merely anecdotal, although notably, not every SCAD patient with *PKD1* variants had polycystic kidneys. Polycystin 1 has been implicated in the structural integrity of blood vessels, providing a plausible genotype-phenotype mechanism for SCAD and potentially a useful paradigm to aid understanding of the coronary biomechanical processes leading to SCAD.^23,24^ The involvement of *PKD1* but not *PKD2* may be explained by the known milder phenotype, especially in females of *PKD2* where renal disease occurs later and fewer intracranial aneurysms are reported. The population prevalence of *PKD2* disease variants is also lower than for *PKD1*.^25–27^ Gene-set analysis identified Loop of Henle development genes, driven by individuals with QVs in *PKD1*, *UMOD*, *HES5*, and *DLL1*, suggesting that the association between SCAD events and renal dysfunction may be more extensive than has been recognized. Experiments in mice would support a role for these genes in SCAD pathogenesis via a direct effect on vessel development or maintenance.^28,29^

We also found pathogenic variants in *COL3A1* and *SMAD3*, which respectively cause vascular Ehlers-Danlos syndrome, and LDS, and have previously been identified in multiple SCAD patients.^6,7^ We also detected variants in *TGFB2*, which is also mutated in LDS, and *MYLK*, where variants are associated with aortic dissection and FMD, where variants have been described in single SCAD patients.^30^ These are the first reported SCAD patients with variants in *LOX* and *YY1AP1*. *LOX* encodes an enzyme that cross-links fibers in connective tissue matrices, and PTVs in this gene cause thoracic aortic aneurysms and dissections,^31^ while homozygous or compound heterozygous PTVs in *YY1AP1* can cause Grange syndrome.^13^ As with our patients with *PKD1* variants, phenotypic expressivity of patients with variants in these genes was variable, with patient not always having classical clinical features typical of vascular Ehlers-Danlos syndrome (*COL3A1*) or LDS (*SMAD3* and *TGFB2*). This lack of phenotypic concordance suggests selecting subsets of SCAD patients for genetic screening based on associated clinical phenotypes would miss some patients with mutations in those genes.

Although we did not find any pathogenic SVs, we did detect an in-frame deletion of *PRKG1* exon 6. *PRKG1* is associated with autosomal dominant familial aortic aneurysm, and the mechanism is thought to be gain-of-function.^32^ It remains possible that the deletion in this SCAD patient produces a gain-of-function as it deletes a single, small, in-frame exon, but this would require further functional studies.

We prioritized a total of 11 genes highly ranked in the collapsing analysis, namely, *PAM, GLI3, SEC24B, COL18A1, NFATC4, ARNTL, TBX2, HDAC9, SOX9, SORBS2*, and *COL4A2*, for future validation. Each of these genes represents a credible candidate for SCAD, based on function, expression, and mouse phenotype, but require validation in future studies. Ten of these genes (bar *PAM*) were also prioritized by our machine-learning tool mantis-ml, demonstrating the complementarity of the approaches and confirming mantis-ml as a promising addition to the gene prioritization toolbox.

### Limitations

Given the low frequency and genetic heterogeneity of SCAD, our study is relatively underpowered for novel gene discovery. Furthermore, while we used the power of genome sequencing to some extent (ie, by investigating deletions), several classes of variation were beyond the scope of this study including novel clinically relevant noncoding variants, more complex SVs, and the contribution of common variants, including polygenic risk. Finally, the SCAD cohort recruited at the Victor Chang Cardiac Research Institute was only adopted for reviewing additional variants in the subset of tier 1 and 2 genes where variants had been found in the UK cohort.

### Conclusions

We have demonstrated that only ≈3.6% of SCAD survivors have a pathogenic variant that is likely responsible for their phenotype. Our study supports previous reports of a connection between *PKD1* and SCAD, indicating a statistically confident association. Moreover, gene-set enrichment analyses suggest the relationship might extend beyond *PKD1* to other renal disease genes. The repertoire of CTD genes reported to be involved in SCAD may also be higher than previously thought, as suggested in this study by the identification of patients with pathogenic variants in *TGFB2*, *LOX*, *MYLK*, and *YY1AP1*. We anticipate that the growing catalog of candidate SCAD risk alleles we have identified here could assist in delineating meaningful genetic endotypes, although the overall contribution of rare variants to disease is small.

## Acknowledgments

We acknowledge the leadership of the ESC-ACVC (European Society of Cardiology, Acute Cardiovascular Care Association) Spontaneous Coronary Artery Dissection (SCAD) Study Group. We thank SCAD study participants and the participants and investigators in the UK biobank study (Resource Application Number 26041), and UK and Australian clinical colleagues who have referred SCAD cases. We acknowledge Jenny Middleton, Jane Plume, Donna Alexander, Sue Sterland, Daniel Lawday, Emma Beeston, Ellie Clarke, Tara Maitland Andrea Marshall, Pamella Mackenzie, Sarah Ford, and Liz Stern. The views presented in this paper are those of the authors and not the NHS, Department of Health or National Institute for Health Research (NIHR). We thank the AstraZeneca Centre for Genomics Research Analytics and Informatics team for processing and analysis of sequencing data.

## Sources of Funding

The sequencing was funded by AstraZeneca’s Centre for Genomics Research, Discovery Sciences, BioPharmaceuticals R&D, and grants from the Cardiac Society of Australia and New Zealand, National Health and Medical Research Council, Australia (grant number APP1161200; Drs Iismaa and Graham), St Vincent’s Clinic Foundation (Dr Graham), Catholic Archdiocese of Sydney (Dr Graham), Perpetual Philanthropy (Dr Graham), NSW Health CVD Clinician-Scientist Grant (Dr Graham), and SCAD Research Inc. The UK SCAD study was supported by the British Heart Foundation (BHF) PG/13/96/30608, the National Institute for Health Research (NIHR) rare disease translational collaboration, the Leicester NIHR Biomedical Research Centre and BeatSCAD. Dr Bouatia-Naji is supported by a European grant from the European Commission (ERC-Stg-ROSALIND-716628). Dr Webb is funded by the funded by the British Heart Foundation (SP/16/4/32697).

## Disclosures

Dr Carss, Dr Armisen, Q. Wang, Dr Deevi, Dr Vitsios, Dr Lewis, Dr Petrovski, and Dr Haefliger are employees of AstraZeneca. Dr Adlam has received research funding from Abbott vascular inc to support a clinical research fellow and has undertaken unrelated consultancy for GE Inc. The other authors report no conflicts.

## Supplementary Material



## References

[1] Jackson R Al-Hussaini A Joseph S van Soest G Wood A Macaya F Gonzalo N Cade J Caixeta A Hlinomaz O. Spontaneous coronary artery dissection: pathophysiological insights from optical coherence tomography. JACC Cardiovasc Imaging. 2019;12:2475–2488. doi: 10.1016/j.jcmg.2019.01.015 30878439 10.1016/j.jcmg.2019.01.015

[2] Adlam D Alfonso F Maas A Vrints C; Writing Committee. European Society of Cardiology, acute cardiovascular care association, SCAD study group: a position paper on spontaneous coronary artery dissection. Eur Heart J. 2018;39:3353–3368. doi: 10.1093/eurheartj/ehy080 29481627 10.1093/eurheartj/ehy080 PMC6148526

[3] Hayes SN Kim ESH Saw J Adlam D Arslanian-Engoren C Economy KE Ganesh SK Gulati R Lindsay ME Mieres JH; American Heart Association Council on Peripheral Vascular Disease; Council on Clinical Cardiology; Council on Cardiovascular and Stroke Nursing; Council on Genomic and Precision Medicine; and Stroke Council. Spontaneous coronary artery dissection: current state of the science: a scientific statement from the American Heart Association. Circulation. 2018;137:e523–e557. doi: 10.1161/CIR.0000000000000564 29472380 10.1161/CIR.0000000000000564 PMC5957087

[4] Nishiguchi T Tanaka A Ozaki Y Taruya A Fukuda S Taguchi H Iwaguro T Ueno S Okumoto Y Akasaka T. Prevalence of spontaneous coronary artery dissection in patients with acute coronary syndrome. Eur Heart J Acute Cardiovasc Care. 2016;5:263–270. doi: 10.1177/2048872613504310 24585938 10.1177/2048872613504310

[5] Goel K Tweet M Olson TM Maleszewski JJ Gulati R Hayes SN. Familial spontaneous coronary artery dissection: evidence for genetic susceptibility. JAMA Intern Med. 2015;175:821–826. doi: 10.1001/jamainternmed.2014.8307 25798899 10.1001/jamainternmed.2014.8307

[6] Henkin S Negrotto SM Tweet MS Kirmani S Deyle DR Gulati R Olson TM Hayes SN. Spontaneous coronary artery dissection and its association with heritable connective tissue disorders. Heart. 2016;102:876–881. doi: 10.1136/heartjnl-2015-308645 26864667 10.1136/heartjnl-2015-308645

[7] Kaadan MI MacDonald C Ponzini F Duran J Newell K Pitler L Lin A Weinberg I Wood MJ Lindsay ME. Prospective cardiovascular genetics evaluation in spontaneous coronary artery dissection. Circ Genom Precis Med. 2018;11:e001933. doi: 10.1161/CIRCGENETICS.117.001933 29650765 10.1161/CIRCGENETICS.117.001933

[8] von Hundelshausen P Oexle K Bidzhekov K Schmitt MM Hristov M Blanchet X Kaemmerer H Matyas G Meitinger T Weber C. Recurrent spontaneous coronary dissections in a patient with a de novo fibrillin-1 mutation without Marfan syndrome. Thromb Haemost. 2015;113:668–670. doi: 10.1160/TH14-11-0913 25519456 10.1160/TH14-11-0913

[9] Sun Y Chen Y Li Y Li Z Li C Yu T Xiao L Yu B Zhao H Tao M. Association of TSR1 variants and spontaneous coronary artery dissection. J Am Coll Cardiol. 2019;74:167–176. doi: 10.1016/j.jacc.2019.04.062 31296287 10.1016/j.jacc.2019.04.062

[10] Turley TN Theis JL Sundsbak RS Evans JM O’Byrne MM Gulati R Tweet MS Hayes SN Olson TM. Rare missense variants in TLN1 are associated with familial and sporadic spontaneous coronary artery dissection. Circ Genom Precis Med. 2019;12:e002437. doi: 10.1161/CIRCGEN.118.002437 30888838 10.1161/CIRCGEN.118.002437 PMC6625931

[11] Adlam D Olson TM Combaret N Kovacic JC Iismaa SE Al-Hussaini A O’Byrne MM Bouajila S Georges A Mishra K; DISCO Consortium; CARDIoGRAMPlusC4D Study Group. Association of the PHACTR1/EDN1 genetic locus with spontaneous coronary artery dissection. J Am Coll Cardiol. 2019;73:58–66. doi: 10.1016/j.jacc.2018.09.085 30621952 10.1016/j.jacc.2018.09.085 PMC10403154

[12] Richards S Aziz N Bale S Bick D Das S Gastier-Foster J Grody WW Hegde M Lyon E Spector E; ACMG Laboratory Quality Assurance Committee. Standards and guidelines for the interpretation of sequence variants: a joint consensus recommendation of the American College of Medical Genetics and Genomics and the Association for Molecular Pathology. Genet Med. 2015;17:405–424. doi: 10.1038/gim.2015.30 25741868 10.1038/gim.2015.30 PMC4544753

[13] Verstraeten A Perik MHAM Baranowska AA Meester JAN Van Den Heuvel L Bastianen J Kempers M Krapels IPC Maas A Rideout A; European/International Fibromuscular Dysplasia Registry and Initiative (FEIRI); Collaborators of the European/International Fibromuscular Dysplasia Registry and Initiative (FEIRI). Enrichment of rare variants in Loeys-Dietz Syndrome genes in spontaneous coronary artery dissection but not in severe fibromuscular dysplasia. Circulation. 2020;142:1021–1024. doi: 10.1161/CIRCULATIONAHA.120.045946 32897753 10.1161/CIRCULATIONAHA.120.045946

[14] Guo DC Duan XY Regalado ES Mellor-Crummey L Kwartler CS Kim D Lieberman K de Vries BBA Pfundt R Schinzel A; University of Washington Center for Mendelian Genomics. Loss-of-function mutations in YY1AP1 lead to grange syndrome and a fibromuscular dysplasia-like vascular disease. Am J Hum Genet. 2017;100:21–30. doi: 10.1016/j.ajhg.2016.11.008 27939641 10.1016/j.ajhg.2016.11.008 PMC5223026

[15] Cameron-Christie S Wolock CJ Groopman E Petrovski S Kamalakaran S Povysil G Vitsios D Zhang M Fleckner J March RE. Exome-based rare-variant analyses in CKD. J Am Soc Nephrol. 2019;30:1109–1122. doi: 10.1681/ASN.2018090909 31085678 10.1681/ASN.2018090909 PMC6551770

[16] Cirulli ET Lasseigne BN Petrovski S Sapp PC Dion PA Leblond CS Couthouis J Lu YF Wang Q Krueger BJ; FALS Sequencing Consortium. Exome sequencing in amyotrophic lateral sclerosis identifies risk genes and pathways. Science. 2015;347:1436–1441. doi: 10.1126/science.aaa3650 25700176 10.1126/science.aaa3650 PMC4437632

[17] Petrovski S Todd JL Durheim MT Wang Q Chien JW Kelly FL Frankel C Mebane CM Ren Z Bridgers J. An exome sequencing study to assess the role of rare genetic variation in pulmonary fibrosis. Am J Respir Crit Care Med. 2017;196:82–93. doi: 10.1164/rccm.201610-2088OC 28099038 10.1164/rccm.201610-2088OC PMC5519963

[18] GTEx Consortium. The genotype-tissue expression (GTEx) project. Nat Genet. 2013;45:580–585. doi: 10.1038/ng.2653 23715323 10.1038/ng.2653 PMC4010069

[19] Vitsios D Petrovski S. Stochastic semi-supervised learning to prioritise genes from high-throughput genomic screens. Am J Hum Genet. 2020;106:659–678. doi: 10.1016/j.ajhg.2020.03.012 32386536 10.1016/j.ajhg.2020.03.012 PMC7212270

[20] Malfait F Francomano C Byers P Belmont J Berglund B Black J Bloom L Bowen JM Brady AF Burrows NP. The 2017 international classification of the Ehlers-Danlos syndromes. Am J Med Genet C Semin Med Genet. 2017;175:8–26. doi: 10.1002/ajmg.c.31552 28306229 10.1002/ajmg.c.31552

[21] Meester JAN Verstraeten A Schepers D Alaerts M Van Laer L Loeys BL. Differences in manifestations of Marfan syndrome, Ehlers-Danlos syndrome, and Loeys-Dietz syndrome. Ann Cardiothorac Surg. 2017;6:582–594. doi: 10.21037/acs.2017.11.03 29270370 10.21037/acs.2017.11.03 PMC5721110

[22] Klingenberg-Salachova F Limburg S Boereboom F. Spontaneous coronary artery dissection in polycystic kidney disease. Clin Kidney J. 2012;5:44–46. doi: 10.1093/ndtplus/sfr158 26069747 10.1093/ndtplus/sfr158 PMC4400459

[23] Varela A Piperi C Sigala F Agrogiannis G Davos CH Andri MA Manopoulos C Tsangaris S Basdra EK Papavassiliou AG. Elevated expression of mechanosensory polycystins in human carotid atherosclerotic plaques: association with p53 activation and disease severity. Sci Rep. 2015;5:13461. doi: 10.1038/srep13461 26286632 10.1038/srep13461 PMC4541068

[24] Hassane S Claij N Lantinga-van Leeuwen IS Van Munsteren JC Van Lent N Hanemaaijer R Breuning MH Peters DJ DeRuiter MC. Pathogenic sequence for dissecting aneurysm formation in a hypomorphic polycystic kidney disease 1 mouse model. Arterioscler Thromb Vasc Biol. 2007;27:2177–2183. doi: 10.1161/ATVBAHA.107.149252 17656674 10.1161/ATVBAHA.107.149252

[25] Demetriou K Tziakouri C Anninou K Eleftheriou A Koptides M Nicolaou A Deltas CC Pierides A. Autosomal dominant polycystic kidney disease-type 2. Ultrasound, genetic and clinical correlations. Nephrol Dial Transplant. 2000;15:205–211. doi: 10.1093/ndt/15.2.205 10648666 10.1093/ndt/15.2.205

[26] Hateboer N v Dijk MA Bogdanova N Coto E Saggar-Malik AK San Millan JL Torra R Breuning M Ravine D. Comparison of phenotypes of polycystic kidney disease types 1 and 2. European PKD1-PKD2 Study Group. Lancet. 1999;353:103–107. doi: 10.1016/s0140-6736(98)03495-3 10023895 10.1016/s0140-6736(98)03495-3

[27] Torra R Badenas C Darnell A Nicolau C Volpini V Revert L Estivill X. Linkage, clinical features, and prognosis of autosomal dominant polycystic kidney disease types 1 and 2. J Am Soc Nephrol. 1996;7:2142–2151 8915974 10.1681/ASN.V7102142

[28] Kitagawa M Hojo M Imayoshi I Goto M Ando M Ohtsuka T Kageyama R Miyamoto S. Hes1 and Hes5 regulate vascular remodeling and arterial specification of endothelial cells in brain vascular development. Mech Dev. 2013;130:458–466. doi: 10.1016/j.mod.2013.07.001 23871867 10.1016/j.mod.2013.07.001

[29] Sörensen I Adams RH Gossler A. DLL1-mediated Notch activation regulates endothelial identity in mouse fetal arteries. Blood. 2009;113:5680–5688. doi: 10.1182/blood-2008-08-174508 19144989 10.1182/blood-2008-08-174508

[30] Giuliani L Di Toro A Disabella E Grasso M Serio A Urtis M Pilotto A Repetto A Valentini A Calliada F. P5539 Genetic heterogeneity of spontaneous coronary artery dissection (SCAD). Eur Heart J. 40 (Supplement_1).ehz746. 0485

[31] Guo DC Regalado ES Gong L Duan X Santos-Cortez RL Arnaud P Ren Z Cai B Hostetler EM Moran R; University of Washington Center for Mendelian Genomics. LOX mutations predispose to thoracic aortic aneurysms and dissections. Circ Res. 2016;118:928–934. doi: 10.1161/CIRCRESAHA.115.307130 26838787 10.1161/CIRCRESAHA.115.307130 PMC4839295

[32] Guo DC Regalado E Casteel DE Santos-Cortez RL Gong L Kim JJ Dyack S Horne SG Chang G Jondeau G; GenTAC Registry Consortium; National Heart, Lung, and Blood Institute Grand Opportunity Exome Sequencing Project. Recurrent gain-of-function mutation in PRKG1 causes thoracic aortic aneurysms and acute aortic dissections. Am J Hum Genet. 2013;93:398–404. doi: 10.1016/j.ajhg.2013.06.019 23910461 10.1016/j.ajhg.2013.06.019 PMC3738837

