# Supplementary material for: Spontaneous Coronary Artery Dissection: Insights on Rare Genetic Variation From Genome Sequencing
Source: Circ Genom Precis Med. 2020 Oct 29;13(6):e003030. doi: 10.1161/CIRCGEN.120.003030 (PMC7748045; doi:10.1161/CIRCGEN.120.003030)
Supplement: Supplementary file 1 [file hcg-13-e003030-s001.pdf]

SUPPLEMENTAL MATERIAL

Contents

Supplementary Methods 3
- Cohorts 3
  - UK SCAD cohort: recruitment and clinical characteristics 3
  - Victor Chang Cardiac Research Institute SCAD cohort: recruitment and clinical characteristics 3
  - UK Biobank cardiovascular-screened controls 3
- Sequencing, alignment, and variant calling 4
- SCAD tiered gene list 5
- Identification and interpretation of pathogenic variants 6
- Collapsing analysis 8
- Review of highly ranked non-significant collapsing analysis results 13
- Mantis-ml 13
- Gene-set enrichment analysis 14

Supplementary Figures 16

Supplementary Tables 24
- Supplementary Table 1 24
- Supplementary Table 2 71
- Supplementary Table 3 76
- Supplementary Table 4 81
- Supplementary Table 5 83
- Supplementary Table 6 85
- Supplementary Table 7 117
- Supplementary Table 8 120
- Supplementary Table 9 125

Supplementary references 142

Supplementary Appendix: Author Contributions 161

Supplementary Methods

Cohorts

UK SCAD cohort: recruitment and clinical characteristics

Patients were recruited to the UK SCAD Registry (ISRCTN42661582) by self-referral, primary care physician referral, or referral from the clinical team at the index presenting hospital, and all had an angiographically confirmed diagnosis of SCAD. Patients with atherosclerotic, traumatic or iatrogenic dissection (except where the latter complicates definite SCAD) were excluded. Angiographic analysis was conducted by two experienced SCAD clinicians (AA-H and DA), blinded to the results of the genetic analysis, and images were classified using a modified Yip and Saw classification. (1) Here we studied a cohort of 384 cases in the UK SCAD registry. Clinical characteristics are given in Table 1.

Victor Chang Cardiac Research Institute SCAD cohort: recruitment and clinical characteristics

Patients were recruited via a social media platform or through direct referral from cardiologists. The diagnosis of SCAD was confirmed by review of coronary angiogram images by an expert interventional cardiologist (DM) blinded to the results of the genetic analysis. Patient information was obtained by phone interviews, review of specialist letters and hospital records. The 92 SCAD patients reported here are from a broader cohort of 292 patients recruited to date and were selected for sequencing based on angiographically-confirmed SCAD, severity of SCAD and/or recurrence.

UK Biobank cardiovascular-screened controls

Controls for association analyses were selected from 502,543 UK Biobank participants. Of these, 151,027 had exome sequencing available at the time of this study. We further limited analyses to the subset of participants with no report of cardiovascular, endocrine, liver, renal, or connective tissue disorders (Supplementary Table 5).

Sequencing, alignment, and variant calling

For the University of Leicester SCAD cohort, genomic DNA from SCAD cases was extracted and underwent paired-end 150bp WGS at Human Longevity Inc using the NovaSeq6000 platform. For SCAD cases, >98% of consensus coding sequence release 22 (CCDS) has at least 10x coverage and average coverage of the CCDS achieved 42-fold read-depth. Genomic DNA from UK Biobank controls underwent paired-end 75bp whole exome sequencing (WES) at Regeneron Pharmaceuticals using the IDT xGen v1 capture kit on the NovaSeq6000 machines. For UK Biobank controls, >95% of CCDS has at least 10x coverage and average CCDS read-depth of 59X. All case and control sequences were processed through the same bioinformatics pipeline, this included re-processing all the UK Biobank controls from their unaligned FASTQ state. A custom-built Amazon Web Services (AWS) cloud compute platform running Illumina DRAGEN Bio-IT Platform Germline Pipeline v3.0.7 was adopted to align the reads to the GRCh38 genome reference and perform small variant SNV and indel calling. SNVs and indels were annotated using SnpEff v4.3 against Ensembl Build 38.92. (2)

Structural variants were called among the SCAD WGS data using the ensemble tool Parliament2, (3) comprising constituent tools BreakDancer, CNVnator, DELLY, LUMPY, and Manta. (4-8) Structural variants were annotated using AnnotSV. (9)

For the Victor Chang Cardiac Research Institute cohort, genomic DNA was extracted and WGS performed using the Illumina HiSeq X Ten platform with 30x coverage. Reads were aligned to the GRCh37 reference genome with Burrows-Wheeler Aligner (10) and variants called with the Genome Analysis Toolkit Best Practices (11). Principal component analysis using 17,453 ethnic-specific SNVs and projection to the 1000 Genomes principal components confirmed ethnicity. Non-relatedness of subjects in the cohort was confirmed with KING (12). Variants were annotated with ANNOVAR (13) against RefSeq (version 01-06-2017) using multiple pathogenicity prediction algorithms.

SCAD tiered gene list

We compiled a tiered list of genes of interest to SCAD based on publicly available gene lists and literature searches. The tiers indicate the current level of evidence for the association of each gene with SCAD. Tier 1 genes (n=6) comprise those that either harbour rare variants reported as pathogenic or likely pathogenic in multiple SCAD patients, or an enrichment of rare missense variants in cases compared to controls. Tier 2 genes (n=124) comprise those that harbour rare, presumed clinically relevant, variants that were found in a single SCAD patient or among patients with connective tissue or vascular disorders, or common variants associated with these disorders. Tier 3 genes (n=303) represent genes of interest contributing to relevant phenotypes in mice (Supplementary Table 1).

Publicly available gene lists and literature searches conducted March-August 2018. PubMed search terms:

"spontaneous coronary artery dissection" or "connective tissue disorder" and "gene"

"spontaneous coronary artery dissection"

"spontaneous coronary artery dissection" and "gene"

"SCAD" and "gene"

"coronary artery spasm" and "gene"

"coronary artery spasm" and "GWAS"

"aneurysm" and "gene"

"aneurysm" and "GWAS"

"Ehlers-Danlos Syndrome" and "vascular" and "gene"

"carotid dissection" and "gene"

"carotid dissection" and "GWAS"

"arterial tortuosity" and "gene"

"arterial tortuosity" and "GWAS"

"migraine" and "gene"

"migraine" and "GWAS"

"hypermobile syndorme" and "gene"

"hypermobile syndorme" and "GWAS"

"arteritis" and "gene"

"arteritis" and "GWAS"

"abnormal vascular smooth muscle" and "gene"

"abnormal vascular smooth muscle" and "GWAS"

"abnormal blood vessel endothelium" and "gene"

"abnormal blood vessel endothelium" and "GWAS"

“abnormal coronary artery” and “gene”

“abnormal coronary artery” and “GWAS”

“fibromuscular dysplasia” and “gene”

“fibromuscular dysplasia” and “GWAS”

“FMD” and “gene”

“FMD” and “GWAS”

“Connective tissue disorder” and “gene” and “vascular”

“Connective tissue disorder” and “GWAS” and “vascular”

“CTD” and “gene” and “vascular”

“CTD” and “GWAS” and “vascular”

“Connective tissue disorder” and “gene”

“Connective tissue disorder” and “GWAS”

“CTD” and “gene”

“CTD” and “GWAS”

Identification and interpretation of pathogenic variants

For the UK SCAD cohort, pathogenic and likely pathogenic SNVs, indels, and SVs were identified using automated filtering followed by manual review and classification according to ACMG guidelines (14).

In detail, first, automated filtering identified SNVs and indels in the 384 SCAD cases that fulfilled all the following criteria:

- DRAGEN Status = PASS
- Quality score (QUAL) >= 30 in all carriers
- Position is covered >=10X in >99% cases
- GnomAD minor allele frequency (popmax exomes) <= 0.001 (0.1%; in autosomes this is equivalent to 1 in 500 individuals sampled from a general population) (15)
- Minor allele frequency in 384 cases < 0.05 (5%)
- Affects a SCAD tier 1 or tier 2 gene
- Variant is ‘high impact’ in a gene for which loss of function is a known disease mechanism OR variant has previously been reported as DM in HGMD pro v2019.2 or pathogenic or likely pathogenic with no conflicts in ClinVar (accessed May 2019). (16, 17)

Next, we performed manual review and classification of SNVs and indels according to ACMG guidelines. (18) The following factors were considered:

- Affected transcript(s)
- Proximity of protein truncating variants to 3' end terminus of gene
- Consistency of variant consequence with previously reported pathogenic variants in the gene
- Consistency of variant genotype with previously reported mode of inheritance of the gene
- Consistency of SCAD phenotype with previously reported phenotype associated with the gene
- If variant has been previously reported as pathogenic, original literature was reviewed wherever possible to ensure the classification within HGMD/ClinVar was consistent with the original report

Potentially pathogenic and likely pathogenic structural variants were also investigated. First, automated filtering identified the SVs in the 384 SCAD cases that fulfilled the following criteria:

- Deletion, because deletions generally have higher probability than other SV types of negatively impacting the function of a gene (19, 20)
- Overlaps with CCDS region of SCAD tier 1 and 2 genes
- Frequency within SCAD cohort < 0.05 (5%) and frequency in external datasets (DGV and 1000 genomes (19, 20)) < 0.01 (1%)
- Supported by more than one caller among Parliament2's ensemble of callers
- Not flagged as low quality by Parliament2

Next, we performed manual review of SVs that passed automated filtering, considering the following factors *in addition to* all the factors already described for SNVs and indels:

- < 100 high quality heterozygous SNV calls within boundaries of deletion
- Supported by both drop in coverage AND insert size/split read data

- Looks high-confidence upon manual inspection of the reads using Integrative Genomics Viewer (21)

Assessment of statistical differences between clinical endpoints and individuals with pathogenic/likely pathogenic variants compared to those without was performed using Fisher's Exact Tests.

Automated filtering of variants in genes identified in the University of Leicester SCAD cohort in the Victor Chang Cardiac Research Institute SCAD cohort was performed using VPOT (22) with the following parameters:

- gnomAD genomes (popmax) minor allele frequency < 0.01
- Missense, nonsense, frameshift, or splicing variants

Loss of function variants in these genes were considered as well as missense variants reported as pathogenic or likely pathogenic in either ClinVar or HGMD. Variants thus identified were confirmed with manual inspection using IGV. (21)

Collapsing analysis

We selected the subset of 357 SCAD cases in the University of Leicester cohort and 13,722 UK Biobank controls who had high quality sequencing data, are unrelated, and of European ancestry. This aimed to minimise the risk of confounding technical artefacts. In detail, the following sample-level criteria for inclusion in the exome-wide collapsing analyses were applied:

- No evidence of contamination (VerifyBamID FREEMIX < 0.04) (23)

- Good coverage (for cases average coverage of CCDS > 1st percentile and < 99th percentile and for controls >= 95% of CCDS with read depth >= 10X and average read depth >=37X and <=130X)
- Percentage of reads that map to reference genome > 1st percentile (cases only)
- Unrelated (i.e. exclude one of each related pair, up to 3rd degree, calculated using PLINK http://pngu.mgh.harvard.edu/purcell/plink/) (24)
- Predicted to be of European ancestry by Peddy (25)
- Concordance between self-declared and genetic prediction of sex
- Ancestry of controls PC1 or PC2 <= 3SDs from mean of those of cases
- Down-sampled controls to harmonise sex and menopause status of cohort with that of cases

On average, at least 10-fold coverage was achieved for 96.7% and 96.6% of the 34.07 megabase pairs (Mbp) of the Consensus Coding Sequence (CCDS; release 22) for case and control subjects respectively. To alleviate confounding effects attributable to differential coverage, we only considered qualifying variants (QVs) affecting a pruned set of 33.13 Mbp (97.2%) of CCDS sites equally represented in HLI WGS and UK Biobank WES data.

Qualifying variants (QVs) are the subset of rare, high-quality, coding SNVs/indels that are considered during collapsing analysis. We used eleven distinct QV models. Selection of QVs was achieved by imposing a series of variant-level filters. Some of these filters were applied to all QV models and some were specific to the eleven distinct QV models. These filters are detailed below.

For all QV models:

- Minimum coverage 10X
- Has annotations in CCDS transcripts (CCDS release 22; ~34Mb)
- Percent alternate reads in homozygous variants >= 0.8
- Percent alternate reads in heterozygous variants >= 0.3 and <= 0.8
- Binomial test of alternate reads proportion p > 0.000001

- Genotype quality score (GQ) >= 30
- Fisher's strand bias score (FS) <= 200 (indels) <= 60 (SNVs)
- Mapping quality score (MQ) >= 40
- Quality score (QUAL) >= 30
- Read position rank sum score (RPRS) >= -2
- Mapping quality rank sum score (MQRS) >= -8
- DRAGEN variant status = PASS
- Binomial test of difference in coverage between cases and controls p 0.000001
- Variant site achieved 10-fold coverage in >=25% of GnomAD samples, and if variant was observed in GnomAD the variant calls in GnomAD achieved exome z score >=2.0, exome MQ >= 30 and exome allele count raw percent >= 50
- Not in a list of 951 observed sequencing artefacts or problematic variants

Additionally, the following requirements are QV model specific.

PTV model:

- Variant consequence impact = high (exon_loss_variant, frameshift_variant, start_lost, stop_gained, stop_lost, splice_acceptor_variant, splice_donor_variant, gene_fusion, bidirectional_gene_fusion, rare_amino_acid_variant, or transcript_ablation)
- LOO (leave one out) MAF <= 0.001
- GnomAD exome global MAF <= 0.001
- GnomAD exome PopMax MAF <= 0.001
- Variant fails any QC requirement (except coverage) in <=4 samples in cohort
- Hardy-Weinberg Equilibrium exact test p-value >= 0.001
- GnomAD exome random forest p >= 0.01 (SNVs) or >= 0.02 (indels)

PTV or rare damaging model

- Same as PTV model
- OR same as rare damaging model

Synonymous model:

- Synonymous_variant
- LOO MAF <= 0.0005
- GnomAD exome global MAF <= 0.00005
- Variant fails any QC requirement (except coverage) in <=2 samples in cohort
- Hardy-Weinberg Equilibrium exact test p-value >= 0.001
- GnomAD exome random forest p >= 0.01 (SNVs) or >= 0.02 (indels)

Ultra-rare damaging model:

- Variant consequence impact = high or moderate (conservative_inframe_deletion, conservative_inframe_insertion, disruptive_inframe_insertion, disruptive_inframe_deletion, missense_variant_splice_region_variant, missense_variant, or protein_altering_variant)
- LOO MAF <= 0.00025
- Not in GnomAD
- Variant fails any QC requirement (except coverage) in <=1 sample in cohort
- Hardy-Weinberg Equilibrium exact test p-value >= 0.001
- REVEL score >= 0.25 (26)

Ultra-rare damaging (MTR) model:

- Same as ultra-rare damaging model
- Missense tolerance ratio (MTR) score <= 0.78 (equivalent to the exome-wide 25% most missense intolerant protein-coding regions) OR intragenic percentile <= 0.5 (equivalent to the transcript's 50% most missense intolerant protein-coding regions)

Rare damaging model:

- Variant consequence impact = high or moderate
- LOO MAF <= 0.0005
- GnomAD exome global MAF <= 0.00005
- Variant fails any QC requirement (except coverage) in <=2 samples in cohort
- Hardy-Weinberg Equilibrium exact test p-value >= 0.001

- GnomAD exome random forest p >= 0.01 (SNVs) or >= 0.02 (indels)
- REVEL score >= 0.25

Rare damaging (MTR) model:

- Same as rare damaging model
- Missense tolerance ratio (MTR) score <= 0.78 OR centile <= 0.5

Flexible non-synonymous model:

- Variant consequence impact = high or moderate
- LOO MAF <= 0.001
- GnomAD exome global MAF <= 0.0005
- GnomAD exome PopMax MAF <= 0.001
- Variant fails any QC requirement (except coverage) in <=4 samples in cohort
- Hardy-Weinberg Equilibrium exact test p-value >= 0.001
- GnomAD exome random forest p >= 0.01 (SNVs) or >= 0.02 (indels)

Flexible non-synonymous (MTR) model:

- Same as flexible non-synonymous model
- Missense tolerance ratio (MTR) score <= 0.78 OR centile <= 0.5

Flexible damaging model:

- Same as flexible non-synonymous model
- REVEL score >= 0.25

Recessive model:

- Variant consequence impact = high or moderate
- Variant homozygous, OR two heterozygous variants in same gene
- LOO MAF <= 0.005
- GnomAD exome global MAF <= 0.005
- GnomAD exome PopMax MAF <= 0.005
- GnomAD exome homozygous raw >= 2
- Variant fails any QC requirement (except coverage) in <=50 samples in cohort

- Hardy-Weinberg Equilibrium exact test p-value >= 0.00001
- GnomAD exome random forest p >= 0.01 (SNVs) or >= 0.02 (indels)

After QVs had been selected, counts of cases that have at least one QV vs those that have no QVs were compared to controls using the two-tailed Fisher's exact test. Our study-wide significance threshold, after Bonferroni correction for the number of genes and models tested was $\alpha = (0.05/[10 \times 18{,}659]) = 2.7{\times}10^{-7}$. Although we retain genome-wide Bonferroni correction as our official significance cut-off ($p<2.7{\times}10^{-7}$), we also assigned a tissue-specific adjusted alpha of $p<4.1x10^{-6}$ considering only 12,069 genes with expression levels above 1.5 TPM based on mean TPM value for the "Artery - Coronary" tissue subtype in the GTEx database (accessed 19/11/2019) and not correcting for 10 differing models. With a goal of identifying a more refined subset of most highly expressed coronary artery tissue genes we further focused on top decile (10%) (n=1,928 genes; adjusted p<2.6x10-5).

Review of highly ranked non-significant collapsing analysis results

Genes that were highly ranked in the collapsing analysis, but not yet achieving study-wide statistical significance were manually evaluated by AAB and TRW. Genes were assessed for reported function, involvement in human disease, human tissue expression, and mouse phenotype using GeneCards, OMIM, Human Protein Atlas, MGI, as well as a broader literature review. (27-30)

Mantis-ml

We also employed mantis-ml v1.5.4 (31), an automated gene prioritisation tool that considers a wealth of publicly available resources to objectively assign probabilities to genes of unknown relevance given an input set of seed genes; here 130 SCAD tier 1 and tier 2 genes (Supplementary Table 1). Automatic feature compilation was performed by providing the following disease/phenotype terms in the input configuration file: heart, cardio, aortic, aorta, coronary, vascular, artery, dissection, fibromuscular, kidney, vessel and connective tissue. Mantis-ml was trained using six different

classifiers: Extra Trees, XGBoost, Random Forest, Gradient Boosting, Support Vector Classifier and feed-forward Deep Neural Net.

Once the mantis-ml genome-wide probabilities of being a SCAD gene were generated, we performed a hypergeometric test to determine whether the top-ranked collapsing analysis genes (i.e. genes achieving a $p<0.05$ in the collapsing analyses) were significantly enriched for the top 5% of mantis-ml SCAD-predicted genes. A statistically significant result from the hypergeometric test would highlight that there are disease-ascertained genes among the top of the collapsing results and the specific genes most likely to be contributing to that enrichment. In parallel, and in addition to generating a permutation-based null, we also performed the hypergeometric enrichment test using the synonymous genetic model to define our empirical null controlling for the underlying case-control configurations.

Gene-set enrichment analysis

We assessed potential enrichment in gene-sets using Megagene (https://github.com/QuanliWang/MegaCollapsing). (32) Briefly, we applied a logistic regression model in which the tally of genes containing QVs in cases and controls are compared, correcting for sex, number of synonymous QVs each individual has in the gene-set, and the exome-wide tally of QVs each individual has in the QV model. We tested a total of 9,339 gene-sets for each QV model.
Apart from the four SCAD gene-sets, these gene-sets are standardised and designed to be disease-agnostic Gene-sets containing two different genes with overlapping CCDS regions were excluded. Gene-sets comprise the following:

1. 8390 gene-sets from Gene-Set Enrichment Analysis (33)
2. 912 gene families from HUGO Gene Nomenclature Committee (34)
3. 37 gene-sets associated with various diseases: cancer (https://www.cancer.gov/tcga), chronic kidney disease (35), epilepsy (32), and SCAD (tier 1, tier 2, tier 3, and the union of the three tiers)

Supplementary Figures

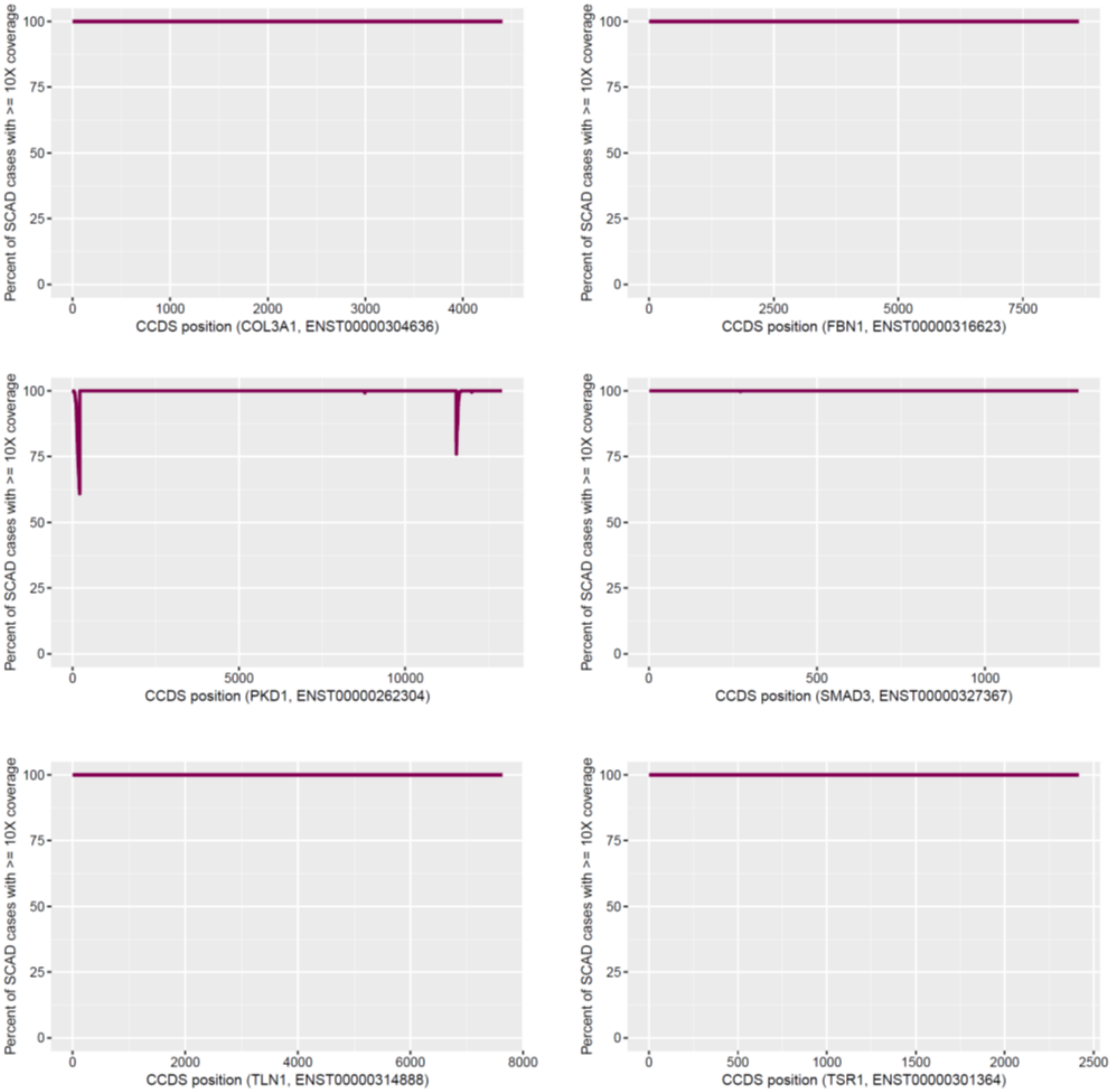

Supplementary Figure 1: Coverage plots for six SCAD tier 1 genes. For each gene the longest coding transcript is shown.

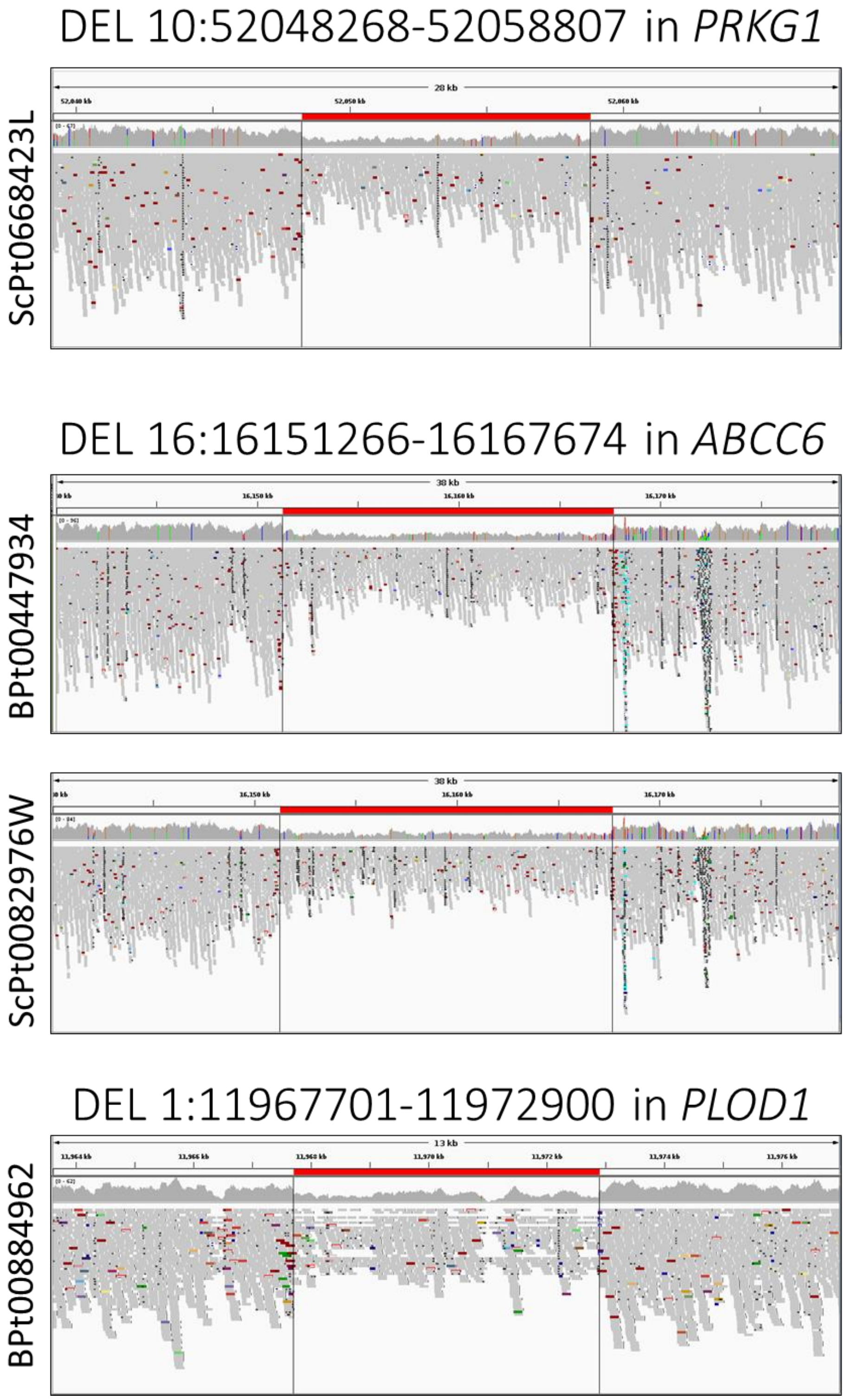

Supplementary Figure 2: Screenshots from Integrative Genomics Viewer showing read alignments of four interesting structural variants identified in SCAD cases.

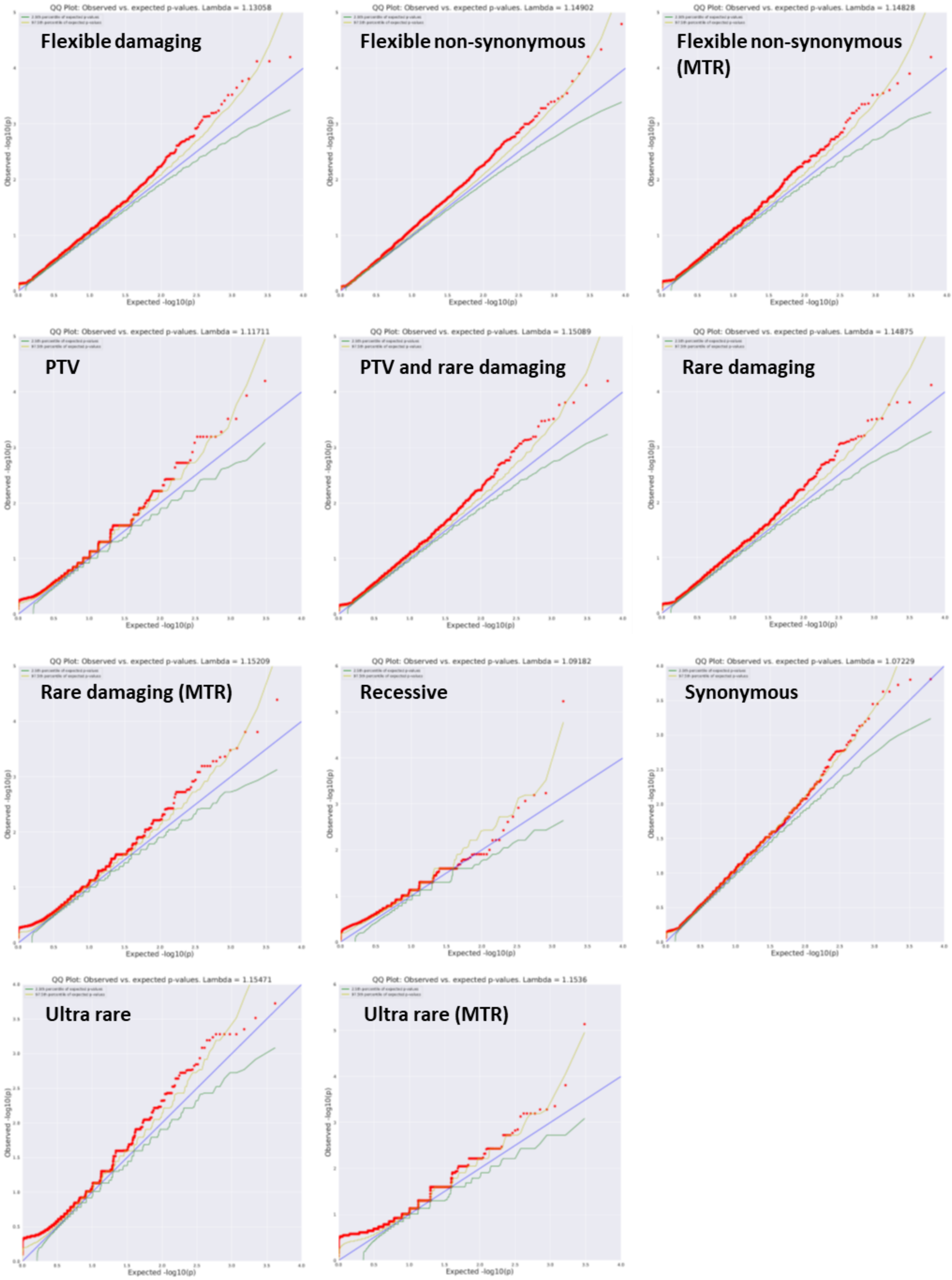

Supplementary Figure 3: Quantile-quantile plots show signals for collapsing analysis; eleven different genetic models tested. MTR = missense tolerance ratio. PTV = protein truncating variant.

Synonymous model included as negative control. No association reached the Bonferroni-corrected study-wide significance threshold of $p < 2.7e-7$.

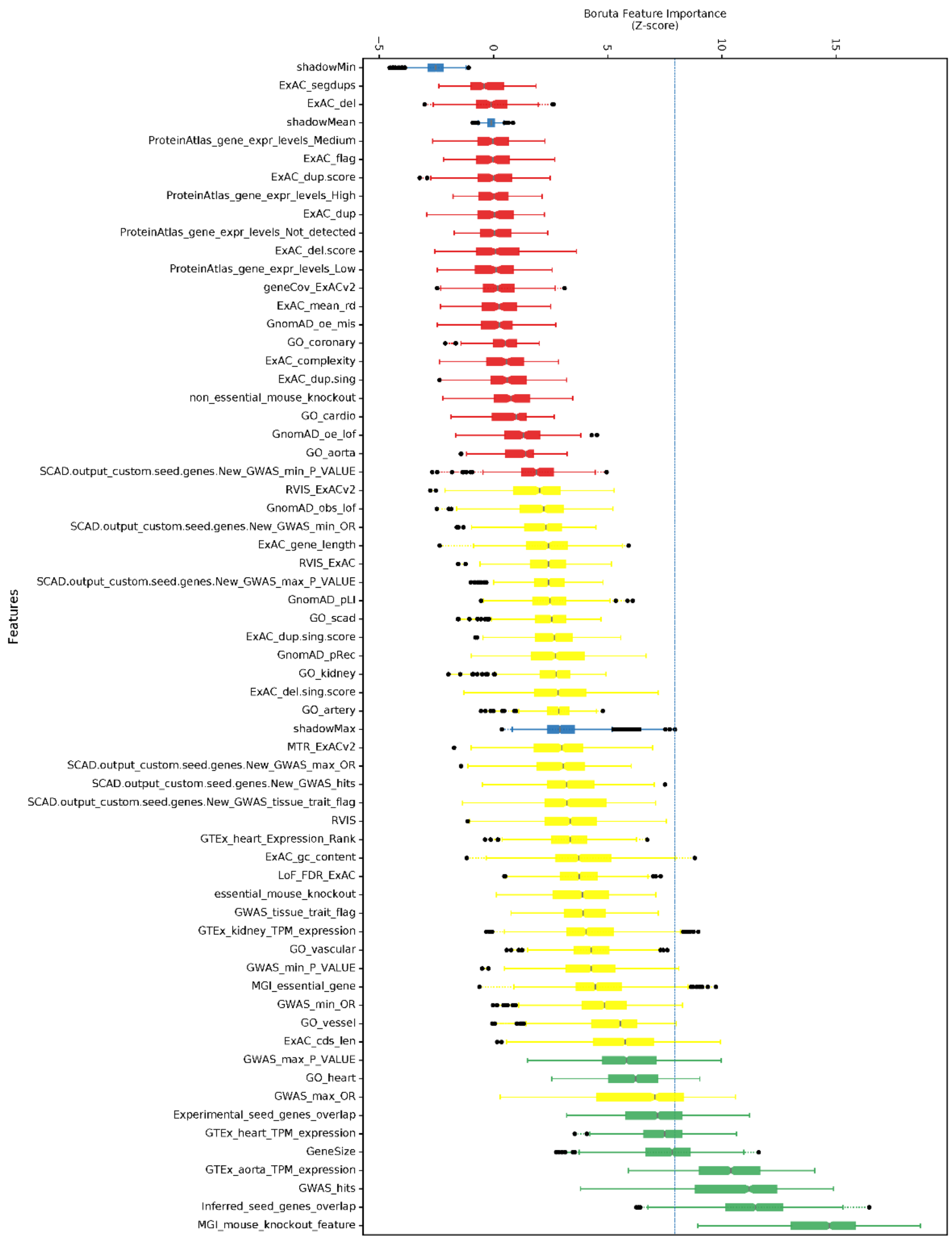

Supplementary Figure 4: Feature importance scores during mantis-ml training. Distribution of feature importance scores extracted by a Random Forest classifier with the Boruta algorithm. Predictions are extracted across ten balanced gene subsets with 10-fold cross-validation for the SCAD-specific case. Confirmed (important) features that are calculated as references by Boruta are shown in in blue ('shadow' features).

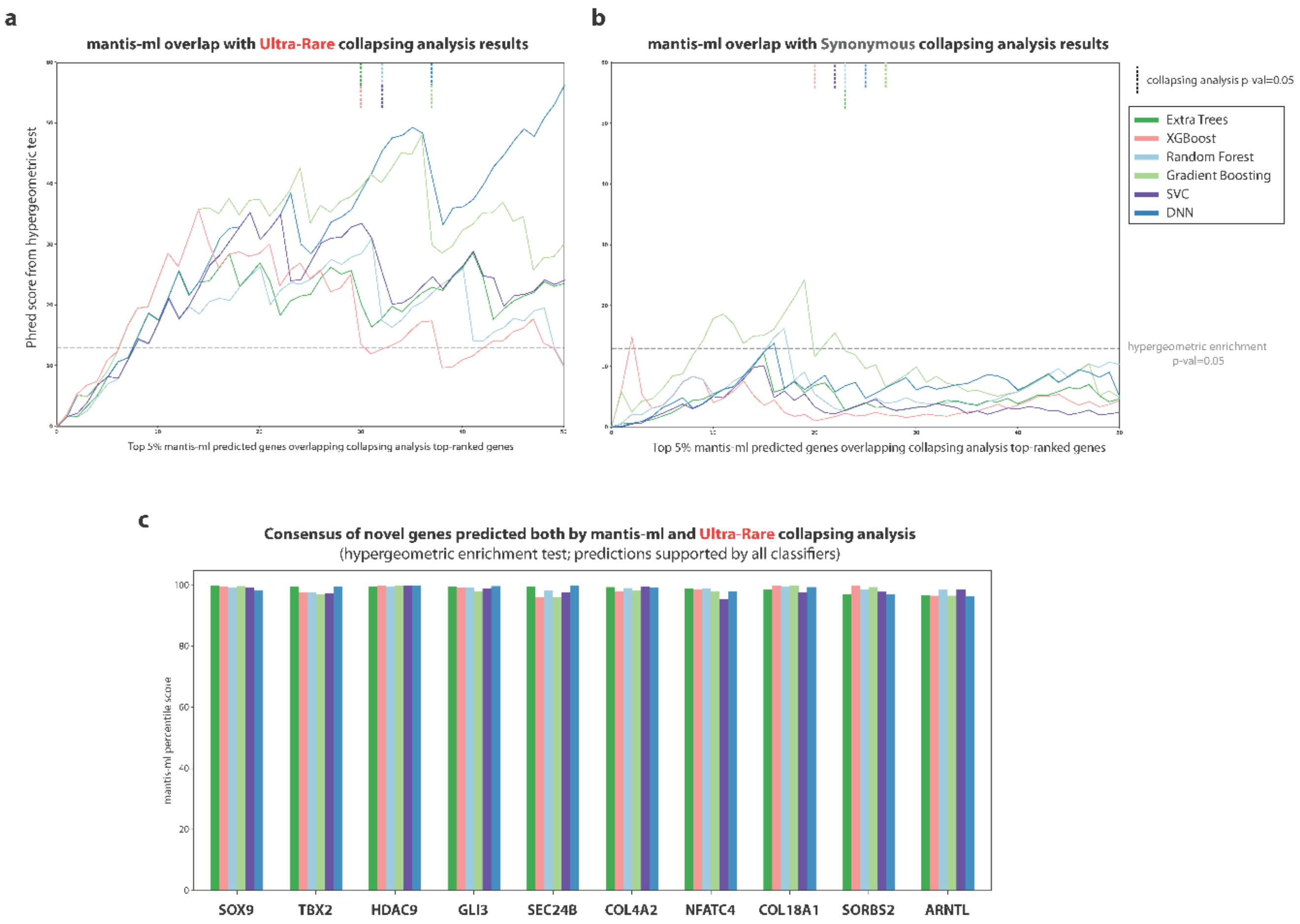

Supplementary Figure 5: Cross-validation of mantis-ml predictions with cohort-level rare-variant association studies. a,b) Hypergeometric test enrichment of SCAD-specific mantis-ml predictions with “ultra-rare variant” and “synonymous variant” collapsing analysis results, respectively. The horizontal dashed grey line corresponds to the significance threshold of p=0.05 for the hypergeometric tests. Where the signal goes above this line it indicates significant enrichment of mantis-ml top gene predictions among the population genomic collapsing analyses. The vertical dashed lines, coloured based on the different classifier ran as part of mantis-ml, indicate the last index of top ranked genes from the collapsing analyses achieving a p-value < 0.05. c) Consensus of genes-of-highest-interest (novel) in SCAD, satisfying the significance threshold criteria in both the collapsing analysis results and the hypergeometric and supported by all six classifiers used by mantis-ml.

Flexible damaging

(est lambda = 1.038)

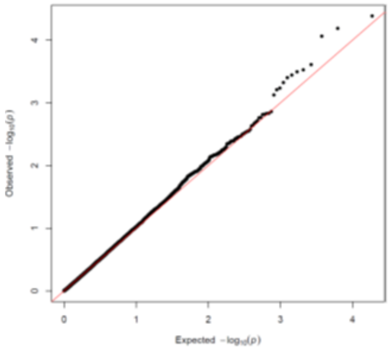

Flexible non-synonymous

(est lambda = 1.019)

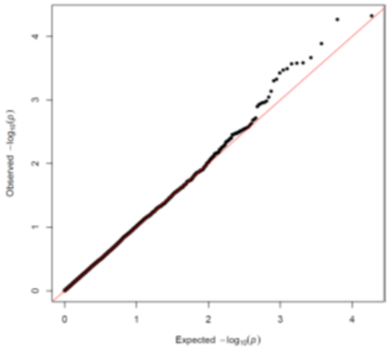

Flexible non-synonymous (MTR)

(est lambda = 0.961)

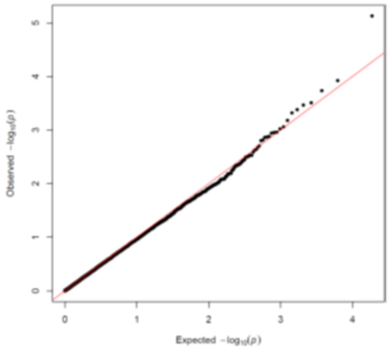

PTV

(est lambda = 0.857)

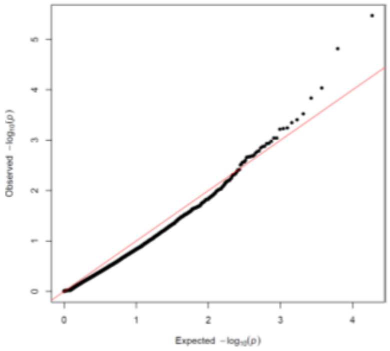

PTV and rare damaging

(est lambda = 1.01)

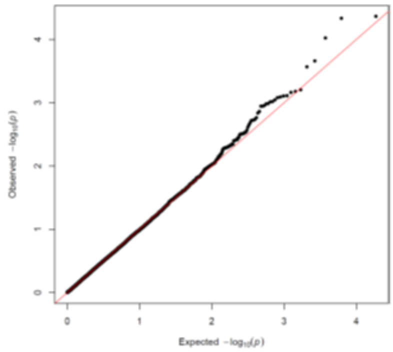

Rare damaging

(est lambda = 1.005)

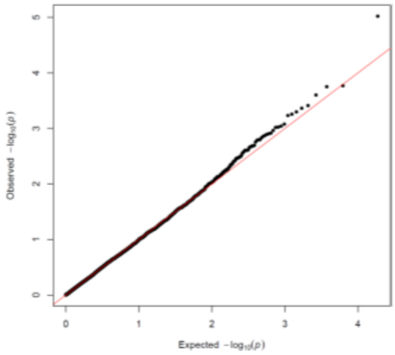

Rare damaging (MTR)

(est lambda = 0.914)

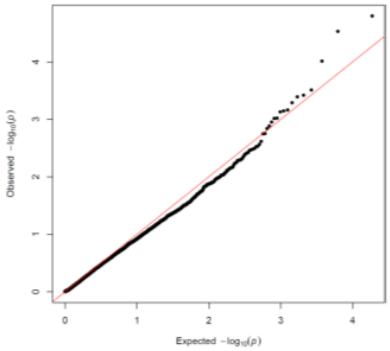

Recessive

(est lambda = 0.827)

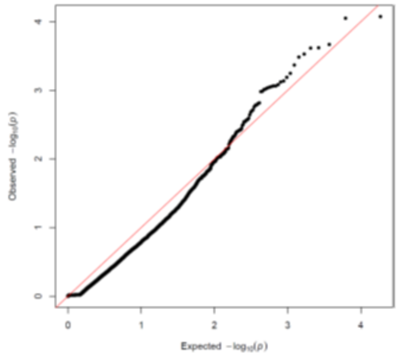

Ultra rare

(est lambda = 1.01)

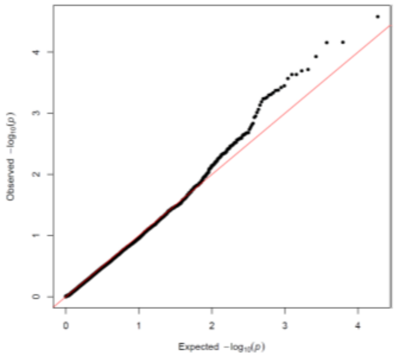

Ultra rare (MTR)

(est lambda = 0.94)

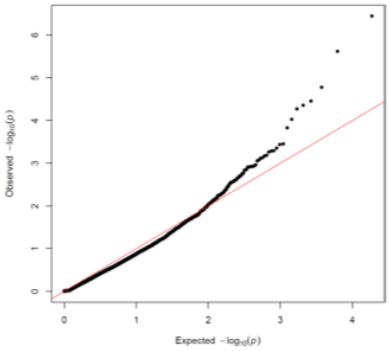

Supplementary Figure 6: Quantile-quantile plots show signals for gene-set enrichment analysis; ten different genetic models tested. MTR = missense tolerance ratio. PTV = protein truncating variant.

Supplementary Tables

Supplementary Table 1

Gene	Gene ID	Tier	OMIM phenotype(s) (accessed 20191105-07) tiers 1-2 only	Other relevant human phenotype(s) from literature search, tiers 1-2 only	Relevant phenotype in mouse model, tier 3	Selected supporting reference(s)
COL3A1	ENSG00000168542	1	Ehlers-Danlos syndrome, vascular type; Polymicrogyria with or without vascular-type EDS	NA	NA	(36-40)
FBN1	ENSG00000166147	1	Acromicric dysplasia; Ectopia lentis, familial; Geleophysic dysplasia 2; Marfan lipodystrophy syndrome; Marfan syndrome; MASS syndrome; Stiff skin syndrome; Weill-Marchesani syndrome 2, dominant	NA	NA	(39, 41, 42)
PKD1	ENSG00000008710	1	Polycystic kidney disease 1	NA	NA	(36, 43, 44)

SMAD3	ENSG00000166949	1	Loeys-Dietz syndrome 3	NA	NA	(36, 45, 46)
TLN1	ENSG00000137076	1	NA	NA	NA	(47)
TSR1	ENSG00000167721	1	NA	NA	NA	(48)
ABCC6	ENSG00000091262	2	Arterial calcification, generalized, of infancy, 2; Pseudoxanthoma elasticum; Pseudoxanthoma elasticum, forme fruste	NA	NA	(49)
ABL1	ENSG00000097007	2	Congenital heart defects and skeletal malformations syndrome; Leukemia, Philadelphia chromosome-positive, resistant to imatinib	NA	NA	(50)
ACTA2	ENSG00000107796	2	Aortic aneurysm, familial thoracic 6; Moyamoya disease 5; Multisystemic smooth muscle dysfunction syndrome	NA	NA	(51, 52)
ACTN4	ENSG00000130402	2	Glomerulosclerosis, focal segmental, 1	Retinal venular tortuosity	NA	(53, 54)
ACVRL1	ENSG00000139567	2	Telangiectasia, hereditary hemorrhagic, type 2	NA	NA	(55)

ADAMTS2	ENSG00000087116	2	Ehlers-Danlos syndrome, dermatosparaxis type	NA	NA	(56)
AEBP1	ENSG00000106624	2	Ehlers-Danlos syndrome, classic-like, 2	NA	NA	(57)
ALDH18A1	ENSG00000059573	2	Cutis laxa, autosomal dominant 3; Cutis laxa, autosomal recessive, type IIIA; Spastic paraplegia 9A, autosomal dominant; Spastic paraplegia 9B, autosomal recessive	NA	NA	(58)
APP	ENSG00000142192	2	Cerebral amyloid angiopathy, Dutch, Italian, Iowa, Flemish, Arctic variants	NA	NA	(59)
ARHGAP31	ENSG00000031081	2	Adams-Oliver syndrome 1	NA	NA	(60)
ARMS2	ENSG00000254636	2	{Macular degeneration, age-related, 8}	Migraine risk loci	NA	(61, 62)
ASTN2	ENSG00000148219	2	NA	Migraine risk loci	NA	(62)
ATP6V0A2	ENSG00000185344	2	Cutis laxa, autosomal recessive, type IIA; Wrinkly skin syndrome	NA	NA	(63)
ATP6V1A	ENSG00000114573	2	Cutis laxa, autosomal recessive, type IID; Epileptic encephalopathy, infantile or	NA	NA	(64)

			early childhood, 3			
B3GALT6	ENSG00000176022	2	Ehlers-Danlos syndrome, spondylodysplastic type, 2; Spondyloepimetaphyseal dysplasia with joint laxity, type 1, with or without fractures	NA	NA	(65)
B4GALT7	ENSG00000027847	2	Ehlers-Danlos syndrome, spondylodysplastic type, 1	NA	NA	(66)
BGN	ENSG00000182492	2	Meester-Loeys syndrome; Spondyloepimetaphyseal dysplasia, X-linked	NA	NA	(67)
C1R	ENSG00000159403	2	Ehlers-Danlos syndrome, periodontal type, 1	NA	NA	(68)
C1S	ENSG00000182326	2	C1s deficiency; Ehlers-Danlos syndrome, periodontal type, 2	NA	NA	(68)
CARF	ENSG00000138380	2	NA	Migraine risk loci	NA	(62)
CD2AP	ENSG00000198087	2	Glomerulosclerosis, focal segmental, 3	Fibromuscular	NA	(69)

				dysplasia risk loci		
CFDP1	ENSG00000153774	2	NA	Migraine risk loci	NA	(62)
CHST14	ENSG00000169105	2	Ehlers-Danlos syndrome, musculocontractural type 1	NA	NA	(70)
COL12A1	ENSG00000111799	2	?Ullrich congenital muscular dystrophy 2; Bethlem myopathy 2	NA	NA	(71)
COL1A1	ENSG00000108821	2	Caffey disease; Ehlers-Danlos syndrome, arthrochalasia type, 1; Osteogenesis imperfecta, types I-IV; {Bone mineral density variation QTL, osteoporosis}	NA	NA	(72-74)
COL1A2	ENSG00000164692	2	Ehlers-Danlos syndrome, arthrochalasia type, 2; Ehlers-Danlos syndrome, cardiac valvular type; Osteogenesis imperfecta, type II-IV; {Osteoporosis, postmenopausal}	NA	NA	(72, 75)
COL27A1	ENSG00000196739	2	Steel syndrome	NA	NA	(72, 76)
COL2A1	ENSG00000139219	2	Achondrogenesis, type II or	NA	NA	(77)

			hypochondrogenesis; Avascular necrosis of the femoral head; Czech dysplasia; Epiphyseal dysplasia, multiple, with myopia and deafness; Kniest dysplasia; Legg-Calve-Perthes disease; Osteoarthritis with mild chondrodysplasia; Platyspondylic skeletal dysplasia, Torrance type; SED congenita; SMED Strudwick type; Spondyloperipheral dysplasia; Stickler syndrome, type I; Vitreoretinopathy with phalangeal epiphyseal dysplasia			
COL5A1	ENSG00000130635	2	Ehlers-Danlos syndrome, classic type, 1	NA	NA	(72, 78)
COL5A2	ENSG00000204262	2	Ehlers-Danlos syndrome, classic type, 2	NA	NA	(36, 39, 72, 79)
COL6A1	ENSG00000142156	2	Bethlem myopathy 1; Ullrich congenital muscular dystrophy 1	NA	NA	(80)

COL6A2	ENSG00000142173	2	?Myosclerosis, congenital; Bethlem myopathy 1; Ullrich congenital muscular dystrophy 1	NA	NA	(80)
COL6A3	ENSG00000163359	2	Bethlem myopathy 1; Dystonia 27; Ullrich congenital muscular dystrophy 1	NA	NA	(81)
COMP	ENSG00000105664	2	Epiphyseal dysplasia, multiple, 1; Pseudoachondroplasia	NA	NA	(82)
CYBA	ENSG00000051523	2	Chronic granulomatous disease, autosomal, due to deficiency of CYBA	Coronary artery spasm risk loci	NA	(83, 84)
DCHS1	ENSG00000166341	2	Mitral valve prolapse 2; Van Maldergem syndrome 1	NA	NA	(85)
DSE	ENSG00000111817	2	Ehlers-Danlos syndrome, musculocontractural type 2	NA	NA	(86)
EDN1	ENSG00000078401	2	Auriculocondylar syndrome 3; Question mark ears, isolated; {High density lipoprotein cholesterol level QTL 7}	NA	NA	(87, 88)
EFEMP2	ENSG00000172638	2	Cutis laxa, autosomal recessive, type IB	Aneurysm; arterial	NA	(89, 90)

				tortuosity risk loci		
ELN	ENSG00000049540	2	Cutis laxa, autosomal dominant; Supravalvar aortic stenosis	NA	NA	(91)
EMILIN1	ENSG00000138080	2	Autosomal dominant connective tissue disorder with peripheral neuropathy (not confirmed)	NA	NA	(92)
ERG	ENSG00000157554	2	NA	Abdominal aortic aneurysm risk loci	NA	(93)
F5	ENSG00000198734	2	Factor V deficiency; Thrombophilia due to activated protein C resistance; {Budd-Chiari syndrome}; {Pregnancy loss, recurrent, susceptibility to, 1}; {Stroke, ischemic, susceptibility to}; {Thrombophilia, susceptibility to, due to factor V Leiden}	NA	NA	(94, 95)
FBLN5	ENSG00000140092	2	?Cutis laxa, autosomal dominant 2; Cutis laxa, autosomal recessive, type IA;	NA	NA	(96)

			Macular degeneration, age-related, 3; Neuropathy, hereditary, with or without age-related macular degeneration			
FBN2	ENSG00000138829	2	Contractural arachnodactyly, congenital; Macular degeneration, early-onset	NA	NA	(97)
FGF6	ENSG00000111241	2	NA	Migraine risk loci	NA	(62)
FHL5	ENSG00000112214	2	NA	Migraine risk loci	NA	(62)
FKBP14	ENSG00000106080	2	Ehlers-Danlos syndrome, kyphoscoliotic type, 2	NA	NA	(98)
FLCN	ENSG00000154803	2	Birt-Hogg-Dube syndrome; Colorectal cancer, somatic; Pneumothorax, primary spontaneous; Renal carcinoma, chromophobe, somatic	NA	NA	(99)
FLNA	ENSG00000196924	2	?FG syndrome 2; Cardiac valvular dysplasia, X-linked; Congenital short bowel syndrome; Frontometaphyseal dysplasia 1; Heterotopia, periventricular,	NA	NA	(100)

			1; Intestinal pseudoobstruction, neuronal; Melnick-Needles syndrome; Otopalatodigital syndrome, type I-II; Terminal osseous dysplasia			
FLNB	ENSG00000136068	2	Atelosteogenesis, types I & III; Boomerang dysplasia; Larsen syndrome; Spondylocarpotarsal synostosis syndrome	NA	NA	(101)
FOXE3	ENSG00000186790	2	Anterior segment dysgenesis 2, multiple subtypes; Cataract 34, multiple types; {Aortic aneurysm, familial thoracic 11, susceptibility to}	NA	NA	(102)
GJA1	ENSG00000152661	2	Atrioventricular septal defect 3; Craniometaphyseal dysplasia, autosomal recessive; Erythrokeratodermia variabilis et progressiva 3; Hypoplastic left heart syndrome 1; Oculodentodigital dysplasia; Palmoplantar keratoderma with congenital	Coronary artery aneurysm (mice); migraine risk loci	NA	(62, 103, 104)

			alopecia; Syndactyly, type III			
GORAB	ENSG00000120370	2	Geroderma osteodysplasticum	NA	NA	(105)
GPR149	ENSG00000174948	2	NA	Migraine risk loci	NA	(62)
GRIN2A	ENSG00000183454	2	Epilepsy, focal, with speech disorder and with or without mental retardation	Arteritis	NA	(106, 107)
HEY2	ENSG00000135547	2	NA	Migraine risk loci	NA	(62)
HJURP	ENSG00000123485	2	NA	Migraine risk loci	NA	(62)
HPSE2	ENSG00000172987	2	Urofacial syndrome 1	Migraine risk loci	NA	(62, 108)
ICAM1	ENSG00000090339	2	{Malaria, cerebral, susceptibility to}	Carotid dissection (unconfirmed)	NA	(109)
IGSF9B	ENSG00000080854	2	NA	Migraine risk loci	NA	(62)
IL12B	ENSG00000113302	2	Immunodeficiency 29, mycobacteriosis	Arteritis	NA	(107, 110)
IL6	ENSG00000136244	2	{Crohn disease-associated growth failure}; {Diabetes, susceptibility to}; {Intracranial hemorrhage in brain cerebrovascular malformations, susceptibility to}; {Kaposi sarcoma, susceptibility to}; {Rheumatoid	Arteritis, coronary artery spasm	NA	(84, 111, 112)

			arthritis, systemic juvenile}			
ITPK1	ENSG00000100605	2	NA	Migraine risk loci	NA	(62)
JAG1	ENSG00000101384	2	?Deafness, congenital heart defects, and posterior embryotoxon; Alagille syndrome 1; Tetralogy of Fallot	Migraine risk loci	NA	(62, 113)
KCNJ2	ENSG00000123700	2	Andersen syndrome; Atrial fibrillation, familial, 9; Short QT syndrome 3	NA	NA	(114)
KCNK5	ENSG00000164626	2	NA	Migraine risk loci	NA	(62)
LILRB3	ENSG00000204577	2	NA	Arteritis	NA	(112)
LMX1B	ENSG00000136944	2	Nail-patella syndrome	NA	NA	(36, 115)
LOX	ENSG00000113083	2	Aortic aneurysm, familial thoracic 10	NA	NA	(116)
LTBP3	ENSG00000168056	2	Dental anomalies and short stature; Geleophysic dysplasia 3	NA	NA	(117)
LTBP4	ENSG00000090006	2	Cutis laxa, autosomal recessive, type IC	NA	NA	(118)
MAT2A	ENSG00000168906	2	Thoracic aortic aneurysm (unconfirmed)	NA	NA	(119)
MED12	ENSG00000184634	2	Lujan–Fryns syndrome; Ohdo syndrome, X-linked; Opitz–Kaveggia syndrome	NA	NA	(120)

MEF2D	ENSG00000116604	2	NA	Migraine risk loci	NA	(62)
MFAP5	ENSG00000197614	2	Aortic aneurysm, familial thoracic 9	NA	NA	(121)
MMP3	ENSG00000149968	2	{Coronary heart disease, susceptibility to, 6}	NA	NA	(84, 122)
MPPED2	ENSG00000066382	2	NA	Migraine risk loci	NA	(62)
MRVI1	ENSG00000072952	2	NA	Migraine risk loci	NA	(62)
MTHFR	ENSG00000177000	2	Homocystinuria due to MTHFR deficiency; {Neural tube defects, susceptibility to}; {Schizophrenia, susceptibility to}; {Thromboembolism, susceptibility to}; {Vascular disease, susceptibility to}	NA	NA	(39, 123)
MYH11	ENSG00000133392	2	Aortic aneurysm, familial thoracic 4	NA	NA	(39, 124)
MYLK	ENSG00000065534	2	Aortic aneurysm, familial thoracic 7	NA	NA	(125)
NCOA7	ENSG00000111912	2	NA	Migraine risk loci	NA	(62)
NOS3	ENSG00000164867	2	{Alzheimer disease, late-onset, susceptibility to}; {Coronary artery spasm	NA	NA	(126)

			1, susceptibility to}; {Hypertension, pregnancy-induced}; {Hypertension, susceptibility to}; {Ischemic stroke, susceptibility to}; {Placental abruption}			
NOTCH1	ENSG00000148400	2	Adams-Oliver syndrome 5; Aortic valve disease 1	NA	NA	(127)
NOTCH4	ENSG00000204301	2	NA	Migraine risk loci	NA	(62)
NRP1	ENSG00000099250	2	NA	Migraine risk loci	NA	(62)
P4HA2	ENSG00000072682	2	Myopia 25, autosomal dominant	Arteritis	NA	(128)
PDIA2	ENSG00000185615	2	NA	aortic valve disease	NA	(129)
PHACTR1	ENSG00000112137	2	Epileptic encephalopathy, early infantile, 70	SCAD risk loci; Migraine risk loci	NA	(62, 87)
PLCE1	ENSG00000138193	2	Nephrotic syndrome, type 3	Migraine risk loci	NA	(62, 130)
PLG	ENSG00000122194	2	Dysplasminogenemia; Plasminogen deficiency, type I	Arteritis	NA	(128, 131)
PLOD1	ENSG00000083444	2	Ehlers-Danlos syndrome, kyphoscoliotic type, 1	NA	NA	(132)

PRDM16	ENSG00000142611	2	Cardiomyopathy, dilated, 1LL; Left ventricular noncompaction 8	Migraine risk loci	NA	(62, 133)
PRDM5	ENSG00000138738	2	Brittle cornea syndrome 2	NA	NA	(134)
PRKG1	ENSG00000185532	2	Aortic aneurysm, familial thoracic 8	NA	NA	(135)
PTGIR	ENSG00000160013	2	NA	Fibromuscular dysplasia	NA	(136)
PYCR1	ENSG00000183010	2	Cutis laxa, autosomal recessive, types IIB & IIIB	NA	NA	(137, 138)
RIN2	ENSG00000132669	2	Macrocephaly, alopecia, cutis laxa, and scoliosis	NA	NA	(139)
ROBO3	ENSG00000154134	2	Gaze palsy, familial horizontal, with progressive scoliosis, 1	NA	NA	(140)
ROBO4	ENSG00000154133	2	Aortic valve disease 8	NA	NA	(141)
RPS9	ENSG00000170889	2	NA	Arteritis	NA	(111)
SDR9C7	ENSG00000170426	2	Ichthyosis, congenital, autosomal recessive 13	Migraine risk loci	NA	(62, 142)
SKI	ENSG00000157933	2	Shprintzen-Goldberg syndrome	NA	NA	(143)

SLC24A3	ENSG00000185052	2	NA	Migraine risk loci	NA	(62)
SLC2A10	ENSG00000197496	2	Arterial tortuosity syndrome	NA	NA	(144)
SLC39A13	ENSG00000165915	2	Ehlers-Danlos syndrome, spondylodysplastic type, 3	NA	NA	(145)
SMAD4	ENSG00000141646	2	Juvenile polyposis/hereditary hemorrhagic telangiectasia syndrome; Myhre syndrome; Pancreatic cancer, somatic; Polyposis, juvenile intestinal	NA	NA	(146)
SMAD6	ENSG00000137834	2	Aortic valve disease 2; {Craniosynostosis 7, susceptibility to}	NA	NA	(147)
SMYD2	ENSG00000143499	2	NA	Aneurysm	NA	(93)
STAT6	ENSG00000166888	2	NA	Migraine risk loci	NA	(62)
SUGCT	ENSG00000175600	2	Glutaric aciduria III	Migraine risk loci	NA	(62, 148)
TBX20	ENSG00000164532	2	Atrial septal defect 4	Aortic valve disease, aneurysm	NA	(149)
TGFB2	ENSG00000092969	2	Loeys-Dietz syndrome 4	NA	NA	(150)
TGFB3	ENSG00000119699	2	Arrhythmogenic right ventricular	NA	NA	(151)

			dysplasia 1; Loeys-Dietz syndrome 5			
TGFBR1	ENSG00000106799	2	Loeys-Dietz syndrome 1; {Multiple self-healing squamous epithelioma, susceptibility to}	NA	NA	(39, 152)
TGFBR2	ENSG00000163513	2	Colorectal cancer, hereditary nonpolyposis, type 6; Esophageal cancer, somatic; Loeys-Dietz syndrome 2	SCAD	NA	(153, 154)
TNXB	ENSG00000168477	2	Ehlers-Danlos syndrome, classic-like, 1; Vesicoureteral reflux 8	NA	NA	(155)
TRPM8	ENSG00000144481	2	NA	Migraine risk loci	NA	(62)
UFL1	ENSG00000014123	2	NA	Migraine risk loci	NA	(62)
YAP1	ENSG00000137693	2	Coloboma, ocular, with or without hearing impairment, cleft lip/palate, and/or mental retardation	Migraine risk loci	NA	(62, 156)
YY1AP1	ENSG00000163374	2	Grange syndrome	NA	NA	(157)
ZCCHC14	ENSG00000140948	2	NA	Migraine risk loci	NA	(62)
ZNF469	ENSG00000225614	2	Brittle cornea syndrome 1	NA	NA	(158)

ADA	ENSG00000196839	3	NA	NA	Abnormal blood vessel morphology	(30)
ADAM19	ENSG00000135074	3	NA	NA	Aneurysm	(30)
ADM	ENSG00000148926	3	NA	NA	Abnormal blood vessel morphology	(30)
ADORA2A	ENSG00000128271	3	NA	NA	Abnormal blood vessel morphology	(30)
AGT	ENSG00000135744	3	NA	NA	Abnormal blood vessel morphology	(30)
AGTR2	ENSG00000180772	3	NA	NA	Aneurysm	(30)
AHR	ENSG00000106546	3	NA	NA	Abnormal blood vessel morphology	(30)
AMACR	ENSG00000242110	3	NA	NA	Migraine	(30)
AMOTL2	ENSG00000114019	3	NA	NA	Abnormal blood vessel morphology	(30)
ANGPT1	ENSG00000154188	3	NA	NA	Abnormal blood vessel morphology	(30)

ANKRD17	ENSG00000132466	3	NA	NA	Abnormal blood vessel morphology	(30)
ANO1	ENSG00000131620	3	NA	NA	Abnormal blood vessel morphology	(30)
APELA	ENSG00000248329	3	NA	NA	Abnormal blood vessel morphology	(30)
APOB	ENSG00000084674	3	NA	NA	Arteritis	(30)
APOE	ENSG00000130203	3	NA	NA	Aneurysm	(30)
ARHGDIA	ENSG00000141522	3	NA	NA	Abnormal blood vessel morphology	(30)
ARHGEF26	ENSG00000277101	3	NA	NA	Abnormal blood vessel morphology	(30)
ARID2	ENSG00000189079	3	NA	NA	Abnormal blood vessel morphology	(30)
ARNTL	ENSG00000133794	3	NA	NA	Abnormal blood vessel morphology	(30)
ATP1A2	ENSG00000018625	3	NA	NA	Migraine	(30)

ATP7A	ENSG00000165240	3	NA	NA	Aneurysm	(30)
BACH1	ENSG00000156273	3	NA	NA	Abnormal blood vessel morphology	(30)
BCAS3	ENSG00000141376	3	NA	NA	Abnormal blood vessel morphology	(30)
BCL2	ENSG00000171791	3	NA	NA	Abnormal blood vessel morphology	(30)
BMP4	ENSG00000125378	3	NA	NA	Abnormal blood vessel morphology	(30)
BMPR1A	ENSG00000107779	3	NA	NA	Abnormal blood vessel morphology	(30)
BRAF	ENSG00000157764	3	NA	NA	Abnormal blood vessel morphology	(30)
C1GALT1	ENSG00000106392	3	NA	NA	Abnormal blood vessel morphology	(30)
CACNA1A	ENSG00000141837	3	NA	NA	Migraine	(30)
CACNB3	ENSG00000167535	3	NA	NA	Abnormal blood	(30)

					vessel morphology	
CALCRL	ENSG00000064989	3	NA	NA	Abnormal blood vessel morphology	(30)
CAV1	ENSG00000105974	3	NA	NA	Abnormal blood vessel morphology	(30)
CAVIN1	ENSG00000177469	3	NA	NA	Abnormal blood vessel morphology	(30)
CAVIN2	ENSG00000168497	3	NA	NA	Abnormal blood vessel morphology	(30)
CAVIN3	ENSG00000170955	3	NA	NA	Abnormal blood vessel morphology	(30)
CBS	ENSG00000160200	3	NA	NA	Aneurysm	(30)
CCM2	ENSG00000136280	3	NA	NA	Abnormal blood vessel morphology	(30)
CCR5	ENSG00000160791	3	NA	NA	Abnormal blood vessel morphology	(30)
CD151	ENSG00000177697	3	NA	NA	Abnormal blood	(30)

					vessel morphology	
CD19	ENSG00000177455	3	NA	NA	Arteritis	(30)
CD40LG	ENSG00000102245	3	NA	NA	Abnormal blood vessel morphology	(30)
CD44	ENSG00000026508	3	NA	NA	Abnormal blood vessel morphology	(30)
CD59	ENSG00000085063	3	NA	NA	Abnormal blood vessel morphology	(30)
CDH5	ENSG00000179776	3	NA	NA	Abnormal blood vessel morphology	(30)
CDKN1A	ENSG00000124762	3	NA	NA	Abnormal blood vessel morphology	(30)
CHD2	ENSG00000173575	3	NA	NA	Arteritis	(30)
CLIC4	ENSG00000169504	3	NA	NA	Abnormal blood vessel morphology	(30)
CLIC5	ENSG00000112782	3	NA	NA	Abnormal blood vessel morphology	(30)

COL15A1	ENSG00000204291	3	NA	NA	Abnormal blood vessel morphology	(30)
COL4A1	ENSG00000187498	3	NA	NA	Migraine	(30)
CREBBP	ENSG00000005339	3	NA	NA	Abnormal blood vessel morphology	(30)
CRIM1	ENSG00000150938	3	NA	NA	Abnormal blood vessel morphology	(30)
CRK	ENSG00000167193	3	NA	NA	Abnormal blood vessel morphology	(30)
CST3	ENSG00000101439	3	NA	NA	Abnormal blood vessel morphology	(30)
CTNNB1	ENSG00000168036	3	NA	NA	Abnormal blood vessel morphology	(30)
CTSS	ENSG00000163131	3	NA	NA	Abnormal blood vessel morphology	(30)
CXCL10	ENSG00000169245	3	NA	NA	Aneurysm	(30)
CXCL12	ENSG00000107562	3	NA	NA	Abnormal blood	(30)

					vessel morphology	
CXCR4	ENSG00000121966	3	NA	NA	Abnormal blood vessel morphology	(30)
DES	ENSG00000175084	3	NA	NA	Abnormal blood vessel morphology	(30)
DLL4	ENSG00000128917	3	NA	NA	Abnormal blood vessel morphology	(30)
DNAAF4	ENSG00000256061	3	NA	NA	Abnormal blood vessel morphology	(30)
DYNC2H1	ENSG00000187240	3	NA	NA	Abnormal blood vessel morphology	(30)
EDNRA	ENSG00000151617	3	NA	NA	Migraine	(30)
EHD3	ENSG00000013016	3	NA	NA	Abnormal blood vessel morphology	(30)
EHD4	ENSG00000103966	3	NA	NA	Abnormal blood vessel morphology	(30)
ELK3	ENSG00000111145	3	NA	NA	Abnormal blood	(30)

					vessel morphology	
ENG	ENSG00000106991	3	NA	NA	Migraine	(30)
ENPP1	ENSG00000197594	3	NA	NA	Abnormal blood vessel morphology	(30)
EP300	ENSG00000100393	3	NA	NA	Arteritis	(30)
ESR1	ENSG00000091831	3	NA	NA	Migraine	(30)
ESR2	ENSG00000140009	3	NA	NA	Abnormal blood vessel morphology	(30)
ETV2	ENSG00000105672	3	NA	NA	Abnormal blood vessel morphology	(30)
F11	ENSG00000088926	3	NA	NA	Abnormal blood vessel morphology	(30)
FAS	ENSG00000026103	3	NA	NA	Arteritis, abnormal blood vessel morphology	(30)
FAT1	ENSG00000083857	3	NA	NA	Aneurysm	(30)
FBLN1	ENSG00000077942	3	NA	NA	Abnormal blood	(30)

					vessel morphology	
FCER1G	ENSG00000158869	3	NA	NA	Arteritis	(30)
FGF8	ENSG00000107831	3	NA	NA	Abnormal blood vessel morphology	(30)
FGFR3	ENSG00000068078	3	NA	NA	Migraine	(30)
FLI1	ENSG00000151702	3	NA	NA	Aneurysm	(30)
FLT1	ENSG00000102755	3	NA	NA	Abnormal blood vessel morphology	(30)
FN1	ENSG00000115414	3	NA	NA	Abnormal blood vessel morphology	(30)
FOLH1	ENSG00000086205	3	NA	NA	Abnormal blood vessel morphology	(30)
FOXC2	ENSG00000176692	3	NA	NA	Abnormal blood vessel morphology	(30)
FOXF1	ENSG00000103241	3	NA	NA	Abnormal blood vessel morphology	(30)
FOXM1	ENSG00000111206	3	NA	NA	Abnormal blood	(30)

					vessel morphology	
GADD45A	ENSG00000116717	3	NA	NA	Abnormal blood vessel morphology	(30)
GATA2	ENSG00000179348	3	NA	NA	Abnormal blood vessel morphology	(30)
GATA4	ENSG00000136574	3	NA	NA	Abnormal blood vessel morphology	(30)
GATA6	ENSG00000141448	3	NA	NA	Abnormal blood vessel morphology	(30)
GDF2	ENSG00000263761	3	NA	NA	Migraine	(30)
GDNF	ENSG00000168621	3	NA	NA	Abnormal blood vessel morphology	(30)
GIPC1	ENSG00000123159	3	NA	NA	Aneurysm	(159)
GIT1	ENSG00000108262	3	NA	NA	Abnormal blood vessel morphology	(30)
GPC3	ENSG00000147257	3	NA	NA	Abnormal blood vessel morphology	(30)

GPR4	ENSG00000177464	3	NA	NA	Abnormal blood vessel morphology	(30)
GULOP	ENSG00000234770	3	NA	NA	Abnormal blood vessel morphology	(30)
H2AFV	ENSG00000105968	3	NA	NA	Abnormal blood vessel morphology	(30)
HDAC7	ENSG00000061343	3	NA	NA	Abnormal blood vessel morphology	(30)
HECTD1	ENSG00000092148	3	NA	NA	Abnormal blood vessel morphology	(30)
HEXIM1	ENSG00000186834	3	NA	NA	Abnormal blood vessel morphology	(30)
HHEX	ENSG00000152804	3	NA	NA	Abnormal blood vessel morphology	(30)
HIF1A	ENSG00000100644	3	NA	NA	Abnormal blood vessel morphology	(30)
HPRT1	ENSG00000165704	3	NA	NA	Abnormal blood	(30)

					vessel morphology	
HSPG2	ENSG00000143498	3	NA	NA	Aneurysm	(30)
HTR1A	ENSG00000178394	3	NA	NA	Migraine	(30)
HTRA1	ENSG00000166033	3	NA	NA	Aneurysm	(30)
IDUA	ENSG00000134415	3	NA	NA	Abnormal blood vessel morphology	(30)
IFNG	ENSG00000111537	3	NA	NA	Aneurysm	(30)
IFNGR1	ENSG00000034697	3	NA	NA	Arteritis	(30)
IL1RN	ENSG00000136689	3	NA	NA	Arteritis	(30)
INS	ENSG00000254647	3	NA	NA	Abnormal blood vessel morphology	(30)
ISL1	ENSG00000016082	3	NA	NA	Abnormal blood vessel morphology	(30)
ITGA7	ENSG00000135424	3	NA	NA	Abnormal blood vessel morphology	(30)
ITGB8	ENSG00000105855	3	NA	NA	Abnormal blood vessel morphology	(30)

JUP	ENSG00000173801	3	NA	NA	Aneurysm	(30)
KCNK18	ENSG00000186795	3	NA	NA	Migraine	(30)
KDR	ENSG00000128052	3	NA	NA	Abnormal blood vessel morphology	(30)
KIRREL1	ENSG00000183853	3	NA	NA	Abnormal blood vessel morphology	(30)
KLF15	ENSG00000163884	3	NA	NA	Abnormal blood vessel morphology	(30)
KLF2	ENSG00000134528	3	NA	NA	Aneurysm	(30)
KRT14	ENSG00000186847	3	NA	NA	Abnormal blood vessel morphology	(30)
LAMA5	ENSG00000130702	3	NA	NA	Abnormal blood vessel morphology	(30)
LAMC1	ENSG00000135862	3	NA	NA	Aneurysm	(30)
LCK	ENSG00000182866	3	NA	NA	Abnormal blood vessel morphology	(30)
LDB1	ENSG00000198728	3	NA	NA	Abnormal blood	(30)

					vessel morphology	
LDLR	ENSG00000130164	3	NA	NA	Aneurysm	(30)
LMNA	ENSG00000160789	3	NA	NA	Abnormal blood vessel morphology	(30)
LPAR4	ENSG00000147145	3	NA	NA	Abnormal blood vessel morphology	(30)
LRP1	ENSG00000123384	3	NA	NA	Aneurysm	(30)
LTBP1	ENSG00000049323	3	NA	NA	Abnormal blood vessel morphology	(30)
MAGI2	ENSG00000187391	3	NA	NA	Abnormal blood vessel morphology	(30)
MAP3K7	ENSG00000135341	3	NA	NA	Abnormal blood vessel morphology	(30)
MAPK7	ENSG00000166484	3	NA	NA	Abnormal blood vessel morphology	(30)
MDK	ENSG00000110492	3	NA	NA	Abnormal blood vessel morphology	(30)

MEGF8	ENSG00000105429	3	NA	NA	Abnormal blood vessel morphology	(30)
METAP2	ENSG00000111142	3	NA	NA	Abnormal blood vessel morphology	(30)
MGAT1	ENSG00000131446	3	NA	NA	Abnormal blood vessel morphology	(30)
MGP	ENSG00000111341	3	NA	NA	Aneurysm	(30)
MICU2	ENSG00000165487	3	NA	NA	Aneurysm	(30)
MMP2	ENSG00000087245	3	NA	NA	Arteritis	(30)
MMP9	ENSG00000100985	3	NA	NA	Aneurysm	(30)
MRTFB	ENSG00000186260	3	NA	NA	Aneurysm	(30)
MSX1	ENSG00000163132	3	NA	NA	Abnormal blood vessel morphology	(30)
MSX2	ENSG00000120149	3	NA	NA	Aneurysm	(30)
MTAP	ENSG00000099810	3	NA	NA	Arteritis	(30)
MT-ATP6	ENSG00000198899	3	NA	NA	Arterial tortuosity	(30)
MT-CO1	ENSG00000198804	3	NA	NA	Migraine	(30)

MT-CO2	ENSG00000198712	3	NA	NA	Migraine	(30)
MT-CO3	ENSG00000198938	3	NA	NA	Arterial tortuosity	(30)
MT-CYB	ENSG00000198727	3	NA	NA	Arterial tortuosity	(30)
MT-ND1	ENSG00000198888	3	NA	NA	Arterial tortuosity	(30)
MT-ND2	ENSG00000198763	3	NA	NA	Arterial tortuosity	(30)
MT-ND4	ENSG00000198886	3	NA	NA	Arterial tortuosity	(30)
MT-ND4L	ENSG00000212907	3	NA	NA	Arterial tortuosity	(30)
MT-ND5	ENSG00000198786	3	NA	NA	Arterial tortuosity	(30)
MT-ND6	ENSG00000198695	3	NA	NA	Arterial tortuosity	(30)
MT-TC	ENSG00000210140	3	NA	NA	Migraine	(30)
MT-TF	ENSG00000210049	3	NA	NA	Migraine	(30)
MT-TK	ENSG00000210156	3	NA	NA	Migraine	(30)
MT-TL1	ENSG00000209082	3	NA	NA	Migraine	(30)
MT-TQ	ENSG00000210107	3	NA	NA	Migraine	(30)
MT-TS1	ENSG00000210151	3	NA	NA	Migraine	(30)
MT-TS2	ENSG00000210184	3	NA	NA	Migraine	(30)
MT-TV	ENSG00000210077	3	NA	NA	Migraine	(30)

MT-TW	ENSG00000210117	3	NA	NA	Migraine	(30)
MUS81	ENSG00000172732	3	NA	NA	Aneurysm	(30)
MYH6	ENSG00000197616	3	NA	NA	Abnormal blood vessel morphology	(30)
MYOCD	ENSG00000141052	3	NA	NA	Abnormal blood vessel morphology	(30)
NCF1	ENSG00000158517	3	NA	NA	Aneurysm	(30)
NDP	ENSG00000124479	3	NA	NA	Migraine	(30)
NES	ENSG00000132688	3	NA	NA	Abnormal blood vessel morphology	(30)
NF1	ENSG00000196712	3	NA	NA	Migraine	(30)
NF2	ENSG00000186575	3	NA	NA	Migraine	(30)
NFATC1	ENSG00000131196	3	NA	NA	Abnormal blood vessel morphology	(30)
NGF	ENSG00000134259	3	NA	NA	Abnormal blood vessel morphology	(30)
NKX2-5	ENSG00000183072	3	NA	NA	Aneurysm	(30)

NLRP3	ENSG00000162711	3	NA	NA	Migraine	(30)
NOS1	ENSG00000089250	3	NA	NA	Abnormal blood vessel morphology	(30)
NOS2	ENSG00000007171	3	NA	NA	Abnormal blood vessel morphology	(30)
NOTCH2	ENSG00000134250	3	NA	NA	Aneurysm	(30)
NOTCH3	ENSG00000074181	3	NA	NA	Migraine	(30)
NPHP3	ENSG00000113971	3	NA	NA	Aneurysm	(30)
NPHS1	ENSG00000161270	3	NA	NA	Abnormal blood vessel morphology	(30)
NPHS2	ENSG00000116218	3	NA	NA	Abnormal blood vessel morphology	(30)
NPPA	ENSG00000175206	3	NA	NA	Abnormal blood vessel morphology	(30)
NTF3	ENSG00000185652	3	NA	NA	Aneurysm	(30)
PARVA	ENSG00000197702	3	NA	NA	Aneurysm	(30)
PAX3	ENSG00000135903	3	NA	NA	Abnormal blood	(30)

					vessel morphology	
PDC	ENSG00000116703	3	NA	NA	Abnormal blood vessel morphology	(30)
PDGFB	ENSG00000100311	3	NA	NA	Migraine	(30)
PDGFRA	ENSG00000134853	3	NA	NA	Abnormal blood vessel morphology	(30)
PDGFRB	ENSG00000113721	3	NA	NA	Aneurysm	(30)
PDPN	ENSG00000162493	3	NA	NA	Abnormal blood vessel morphology	(30)
PECAM1	ENSG00000261371	3	NA	NA	Abnormal blood vessel morphology	(30)
PGK1	ENSG00000102144	3	NA	NA	Abnormal blood vessel morphology	(30)
PGR	ENSG00000082175	3	NA	NA	Abnormal blood vessel morphology	(30)
PIEZO1	ENSG00000103335	3	NA	NA	Abnormal blood vessel morphology	(30)

PIP	ENSG00000159763	3	NA	NA	Arteritis	(30)
PLEKHA1	ENSG00000107679	3	NA	NA	Abnormal blood vessel morphology	(30)
PLEKHG5	ENSG00000171680	3	NA	NA	Abnormal blood vessel morphology	(30)
PLOD3	ENSG00000106397	3	NA	NA	Abnormal blood vessel morphology	(30)
PLVAP	ENSG00000130300	3	NA	NA	Abnormal blood vessel morphology	(30)
PLXND1	ENSG00000004399	3	NA	NA	Abnormal blood vessel morphology	(30)
POMGNT1	ENSG00000085998	3	NA	NA	Aneurysm	(30)
PPARA	ENSG00000186951	3	NA	NA	Abnormal blood vessel morphology	(30)
PRKCD	ENSG00000163932	3	NA	NA	Abnormal blood vessel morphology	(30)
PROC	ENSG00000115718	3	NA	NA	Abnormal blood	(30)

					vessel morphology	
PROS1	ENSG00000184500	3	NA	NA	Abnormal blood vessel morphology	(30)
PROX1	ENSG00000117707	3	NA	NA	Aneurysm	(30)
PRRT2	ENSG00000167371	3	NA	NA	Migraine	(30)
PSAP	ENSG00000197746	3	NA	NA	Abnormal blood vessel morphology	(30)
PSEN1	ENSG00000080815	3	NA	NA	Arteritis	(30)
PSEN2	ENSG00000143801	3	NA	NA	Arteritis	(30)
PTK2	ENSG00000169398	3	NA	NA	Abnormal blood vessel morphology	(30)
PTPN12	ENSG00000127947	3	NA	NA	Abnormal blood vessel morphology	(30)
PTPRJ	ENSG00000149177	3	NA	NA	Abnormal blood vessel morphology	(30)
RAMP2	ENSG00000131477	3	NA	NA	Abnormal blood vessel morphology	(30)

RAPGEF1	ENSG00000107263	3	NA	NA	Abnormal blood vessel morphology	(30)
RARB	ENSG00000077092	3	NA	NA	Abnormal blood vessel morphology	(30)
RASIP1	ENSG00000105538	3	NA	NA	Abnormal blood vessel morphology	(30)
RECK	ENSG00000122707	3	NA	NA	Abnormal blood vessel morphology	(30)
REN	ENSG00000143839	3	NA	NA	Abnormal blood vessel morphology	(30)
ROBO2	ENSG00000185008	3	NA	NA	Migraine	(30)
ROCK1	ENSG00000067900	3	NA	NA	Abnormal blood vessel morphology	(30)
RPL11	ENSG00000142676	3	NA	NA	Migraine	(30)
RPL15	ENSG00000174748	3	NA	NA	Migraine	(30)
RPL26	ENSG00000161970	3	NA	NA	Migraine	(30)
RPL35A	ENSG00000182899	3	NA	NA	Migraine	(30)

RPL5	ENSG00000122406	3	NA	NA	Migraine	(30)
RPS10	ENSG00000124614	3	NA	NA	Migraine	(30)
RPS17	ENSG00000182774	3	NA	NA	Migraine	(30)
RPS19	ENSG00000105372	3	NA	NA	Migraine	(30)
RPS24	ENSG00000138326	3	NA	NA	Migraine	(30)
RPS26	ENSG00000197728	3	NA	NA	Migraine	(30)
RPS29	ENSG00000213741	3	NA	NA	Migraine	(30)
RPS7	ENSG00000171863	3	NA	NA	Migraine	(30)
RRM2B	ENSG00000048392	3	NA	NA	Abnormal blood vessel morphology	(30)
RUNX1	ENSG00000159216	3	NA	NA	Aneurysm	(30)
RYR1	ENSG00000196218	3	NA	NA	Abnormal blood vessel morphology	(30)
S1PR1	ENSG00000170989	3	NA	NA	Abnormal blood vessel morphology	(30)
S1PR2	ENSG00000267534	3	NA	NA	Abnormal blood vessel morphology	(30)

S1PR3	ENSG00000213694	3	NA	NA	Abnormal blood vessel morphology	(30)
SCARB1	ENSG00000073060	3	NA	NA	Abnormal blood vessel morphology	(30)
SCHIP1	ENSG00000151967	3	NA	NA	Abnormal blood vessel morphology	(30)
SCN1A	ENSG00000144285	3	NA	NA	Migraine	(30)
SCN2A	ENSG00000136531	3	NA	NA	Migraine	(30)
SELP	ENSG00000174175	3	NA	NA	Abnormal blood vessel morphology	(30)
SERPIND1	ENSG00000099937	3	NA	NA	Abnormal blood vessel morphology	(30)
SFTPC	ENSG00000168484	3	NA	NA	Abnormal blood vessel morphology	(30)
SGCB	ENSG00000163069	3	NA	NA	Aneurysm	(30)
SGCD	ENSG00000170624	3	NA	NA	Abnormal blood vessel morphology	(30)

SGPL1	ENSG00000166224	3	NA	NA	Abnormal blood vessel morphology	(30)
SHC1	ENSG00000160691	3	NA	NA	Abnormal blood vessel morphology	(30)
SHH	ENSG00000164690	3	NA	NA	Abnormal blood vessel morphology	(30)
SLC1A3	ENSG00000079215	3	NA	NA	Migraine	(30)
SLC20A2	ENSG00000168575	3	NA	NA	Migraine	(30)
SLC2A1	ENSG00000117394	3	NA	NA	Migraine	(30)
SMAD2	ENSG00000175387	3	NA	NA	Abnormal blood vessel morphology	(30)
SMAD5	ENSG00000113658	3	NA	NA	Abnormal blood vessel morphology	(30)
SMAD7	ENSG00000101665	3	NA	NA	Abnormal blood vessel morphology	(30)
SMARCA4	ENSG00000127616	3	NA	NA	Abnormal blood vessel morphology	(30)

SOX17	ENSG00000164736	3	NA	NA	Aneurysm	(30)
SOX18	ENSG00000203883	3	NA	NA	Abnormal blood vessel morphology	(30)
SOX2	ENSG00000181449	3	NA	NA	Abnormal blood vessel morphology	(30)
SOX4	ENSG00000124766	3	NA	NA	Abnormal blood vessel morphology	(30)
SOX9	ENSG00000125398	3	NA	NA	Abnormal blood vessel morphology	(30)
SPHK1	ENSG00000176170	3	NA	NA	Abnormal blood vessel morphology	(30)
SPHK2	ENSG00000063176	3	NA	NA	Abnormal blood vessel morphology	(30)
SPP1	ENSG00000118785	3	NA	NA	Aneurysm	(30)
SPRR3	ENSG00000163209	3	NA	NA	Abnormal blood vessel morphology	(30)
SRF	ENSG00000112658	3	NA	NA	Abnormal blood	(30)

					vessel morphology	
SSBP2	ENSG00000145687	3	NA	NA	Arteritis	(30)
STIM1	ENSG00000167323	3	NA	NA	Migraine	(30)
STK11	ENSG00000118046	3	NA	NA	Abnormal blood vessel morphology	(30)
STK3	ENSG00000104375	3	NA	NA	Abnormal blood vessel morphology	(30)
STK4	ENSG00000101109	3	NA	NA	Abnormal blood vessel morphology	(30)
SUFU	ENSG00000107882	3	NA	NA	Aneurysm	(30)
TAGLN	ENSG00000149591	3	NA	NA	Aneurysm	(30)
TBX1	ENSG00000184058	3	NA	NA	Abnormal blood vessel morphology	(30)
TBX18	ENSG00000112837	3	NA	NA	Abnormal blood vessel morphology	(30)
TDG	ENSG00000139372	3	NA	NA	Abnormal blood vessel morphology	(30)

TEK	ENSG00000120156	3	NA	NA	Abnormal blood vessel morphology	(30)
TFAP2A	ENSG00000137203	3	NA	NA	Abnormal blood vessel morphology	(30)
THBS1	ENSG00000137801	3	NA	NA	Abnormal blood vessel morphology	(30)
THY1	ENSG00000154096	3	NA	NA	Abnormal blood vessel morphology	(30)
TIE1	ENSG00000066056	3	NA	NA	Abnormal blood vessel morphology	(30)
TIMP1	ENSG00000102265	3	NA	NA	Aneurysm	(30)
TIMP3	ENSG00000100234	3	NA	NA	Aneurysm	(30)
TK1	ENSG00000167900	3	NA	NA	Arteritis	(30)
TMSB4X	ENSG00000205542	3	NA	NA	Abnormal blood vessel morphology	(30)
TNF	ENSG00000232810	3	NA	NA	Aneurysm	(30)
TNFRSF1A	ENSG00000067182	3	NA	NA	Migraine	(30)

TNNT2	ENSG00000118194	3	NA	NA	Abnormal blood vessel morphology	(30)
TSPAN12	ENSG00000106025	3	NA	NA	Aneurysm	(30)
TUSC2	ENSG00000114383	3	NA	NA	Arteritis	(30)
VAV2	ENSG00000160293	3	NA	NA	Abnormal blood vessel morphology	(30)
VEGFA	ENSG00000112715	3	NA	NA	Abnormal blood vessel morphology	(30)
VEZF1	ENSG00000136451	3	NA	NA	Abnormal blood vessel morphology	(30)
VHL	ENSG00000134086	3	NA	NA	Abnormal blood vessel morphology	(30)
WNT1	ENSG00000125084	3	NA	NA	Abnormal blood vessel morphology	(30)
ZFAND5	ENSG00000107372	3	NA	NA	Abnormal blood vessel morphology	(30)

Supplementary Table 1: Tiered list of genes of interest to SCAD. Tier 1 genes harbour rare variants previously reported as pathogenic in multiple SCAD patients. Tier 2 genes harbour rare variants previously reported as being pathogenic in a single SCAD patient or patients with connective tissue disorders or vascular disorders or common variants associated with these disorders. Tier 3 genes are of interest based on relevant phenotypes in mouse models.

Supplementary Table 2

Gene	Variant (GRCh38)	Transcript	Transcript codon change	Transcript AA change	Variant Type	Sample(s)	N case carriers	GnomAD global AF	GnomAD popmax AF	Reference (if previously reported)
ABCC6	16-16177610-G-A	ENST00000205557	c.2432C>T	p.Thr811Met	Missense variant	ScPt0231641G	1	2.8E-5	9.7E-5	(160)
ABCC6	g.16:16151266_16167674del	ENST00000205557	c.2996-1741_4209-494del (exons 23-29)	p.Ala999fs	Structural variant	BPt00447934, ScPt0082976W	2	1.8E-4	3.9E-4	(161)
ADAMTS2	g.5:179124880_179125089del	ENST00000251582	c.2842_2958+93del	p.Asp948_Gln986del	Splice donor variant	BPt00937431, ScPt0850606K	2	0	0	NA
ATP6V0A2	12-	ENST00000330342	c.1246G>A	p.Gly416Arg	Missense	ScPt0250750V	1	8.0E-6	3.2E-5	(162)

	123744257-G-A				variant					
B3GALT6	1-1232791-C-CGCCCGCGA	ENST00000379198	c.521_528dupAGCCCGCG	p.Arg177fs	Frameshift variant	ScPt0761346G	1	0	0	NA
B4GALT7	5-177607462-CCT-C	ENST00000029410	c.579_580delCT	p.Tyr194fs	Frameshift variant	ScPt0666722L	1	4.0E-6	9.0E-8	NA
FLNA	X-154364582-G-A	ENST00000360319	c.1966C>T	p.Leu656Phe	Missense variant	ScPt0047580S	1	6.0E-6	7.8E-5	(163)
GORAB	1-170552151-T-TTGGAGGAGTTGATG	ENST00000367763	c.877_901dupGAGGAGTTGATGCAACAACTAGATG	p.Val301fs	Frameshift variant	BPt00447934	1	0	0	NA

	CAACAAC TAGA									
LTBP3	11-65540485-C-G	ENST00000301873	c.3106+1G>C	NA	Splice donor variant	ScPt0197004C	1	0	0	NA
LTBP3	11-65540034-G-A	ENST00000301873	c.3364C>T	p.Gln1122*	Stop gained	ScPt0773459E	1	9.0E-6	2.4E-5	(17)
MTHFR	1-11802980-C-T	ENST00000376486	c.137G>A	p.Arg46Gln	Missense variant	ScPt0090027W	1	2.0E-5	3.3E-5	(164)
MTHFR	1-11795125-C-T	ENST00000376583	c.1127G>A	p.Arg376His	Missense variant	ScPt0159952X	1	4.9E-5	1.3E-4	(164)
PLCE1	10-94316621-TAAAGA-T	ENST00000371375	c.5290_5294 delAAGAA	p.Lys1764fs	Frameshift variant	ScPt0027347Z	1	0	0	NA
PLOD1	g.1:11967701_11972900	ENST00000196061	c.1755+610_1931del	p.Asp586fs	Structural variant	BPt00884962	1	0	0	(165)

	del		(exon 17 and part of 18)							
PYCR1	17-81934326-C-T	ENST00000329875	c.797G>A	p.Arg266Gln	Missense variant & splice region variant	ScPt0985939L	1	7.4E-5	1.5E-4	(138)
RIN2	20-19974941-T-TGC	ENST00000255006	c.1063_1064 insGC	p.Ser355fs	Frameshift variant	ScPt0658102Y	1	0	0	NA
TNXB	6-32072272-CTG-C	ENST00000375244	c.4706_4707 delCA	p.Thr1569fs	Frameshift variant	ScPt0049874S	1	2.5E-5	6.7E-5	NA

Supplementary Table 2: Single heterozygous variants in SCAD patients in tier 1 or 2 genes associated with recessive connective tissue disorders or vascular disorders. These variants would be considered pathogenic or likely pathogenic if they were biallelic, but none are confidently causative of SCAD in this study because they are all monoallelic (heterozygous). For SNVs and indels GnomAD exomes allele frequencies are shown and for SVs GnomAD genomes allele frequencies are shown.

Supplementary Table 3

Variant (GRCh38)	Gene	Clinical characteristics														Family history
		P	IE	ES	RA	Aortic root Dimensions	MV	SA	Skin	CT	HM	HT	Stroke	CAD	Description	
1-155660577-T-A	*YY1AP1*	0	NA	No	No*	STJ 20mm, AA 20mm	No	Yes	No	No	No	Yes	Yes	No	FMD; renal artery stenosis;, brachydactyly; migraines	NR
1-218434118-C-T	*TGFB2*	4	No	Yes	Yes	SoV 33mm	No	Yes	Yes	No	No	Yes	No	No	Left carotid artery dissection; right internal carotid aneurysm; left vertebral pseudo	NR

															aneurysm and dissection	
1-218436110-C-T	*TGFB2*	2	Yes	No	No*	SoV 33mm, STJ 24mm, AA 28mm	No	Yes	Yes	No	Yes	Yes	No	Yes	Chiari malformation, easy bruising; hypothyroidism	Chiari malformation in sister and son
15-67066155-A-T	*SMAD3*	4	NA	NA	No*	SoV 28mm, AA 30mm	No	Yes	Yes	No	No	No	No	No	Easy bruising; pectus carinatum; march fractures	Dissecting aortic aneurysm in father
15-67190432-A-AC	*SMAD3*	2	No	Yes	No*	SoV 27mm, AA 28mm	No	No	No	No	No	No	No	No	NR	Aortic aneurysm in maternal grandfather
16-2090692-G-C	*PKD1*	3	Yes	Yes	No	SoV 30mm, STJ 33mm, AA 30mm	Yes	No	Yes	No	No	Yes	No	Yes	PCKD; mildly ectactic aorta (3.2 cm); mitral	PCKD in mother/maternal

															valve prolapse; psoriasis	grandmother; ruptured berry aneurysm in maternal grandmother
16-2106443-CCA-C	*PKD1*	1	Yes	Yes	No*	SoV 29mm, AA 30mm	No	No	Yes	Yes	Yes	Yes	No	No	PCKD; easy bruising	NR
16-2106665-G-A	*PKD1*	1	No	Yes	No	SoV 27mm, SJ 20mm, AA 27mm	No	No	No	No	No	No	No	No	NR	NR
16-2109337-C-T	*PKD1*	1	No	No	No	SoV 34mm, STJ 27mm AA 27mm	No	No	Yes	No	Yes	No	No	No	EDS-like phenotype; easy bruising	HM in son; SCAD in 1st cousin
16-2118102-G-C	*PKD1*	2	No	No	No*	SoV 34mm, STJ 35mm AA 32mm	No	No	No	No	No	No	No	No	Aortic root dimension at upper limit of normal	NR

2-188990117-C-T	*COL3A1*	1	Yes	Yes	No	SoV 23mm, STJ 20mm, AA 24mm	No	Yes	No	No	No	No	No	Yes	Scoliosis; subconjunctival haemorrhage	Scoliosis in mother
2-189011668-G-T	*COL3A1*	2	Yes	Yes	No	SoV 31mm, STJ 24mm, AA 26mm	No	Yes	No	No	No	No	No	No	High palate; pes planus	Patella dislocation in brother; Recurrent pneumothorax in brother
3-123708719-G-A	*MYLK*	0	No	No	No	SoV 32mm, STJ 24mm, AA 29mm	No	No	No	No	No	No	No	No	Dyslipidemia; systemic inflammatory disease	NR
5-122074155-A-C	*LOX*	1	Yes	Yes	Yes	SoV 35mm, STJ 26mm, AA 30mm	No	No	No	No	No	No	No	Yes	FMD; right internal carotid dissection; mildly dilated	Intracerebral bleed secondary to aneurysm in

															aorta; dislocation of ankles	mother

Supplementary Table 3: Clinical information regarding patients affected by pathogenic and likely pathogenic variants. All the subjects with pathogenic variants are white females and none had pregnancy-related SCAD or recurrence. P= number of pregnancies defined as gestation ending with delivery. IE = intense exercise. ES = emotional stress. RA = remote arteriopathy as defined as any arterial abnormality and may include dilations or narrowing's of arteries outside normal limits, dissections, aneurysms and fibromuscular dysplasia but does not include arterial tortuosity. * = incomplete screening for remote arteriopathies. MV = mitral valve prolapse. SA = skeletal abnormalities. Skin = skin abnormalities. HTN = hypertension. CAD = family history of CAD. CTD = Connective Tissue disorder (includes PCKD). HM = Hypermobility. SoV = sinus of Valsalva. STJ = sinotubular junction. AA = ascending aorta. NA = not available. NR = no relevant data.

Supplementary Table 4

Trait	Case definition (n)	Ctrl definition (n)	n cases solved	% cases solved	n ctrl solved	% ctrl solved	P value	OR	OR LCI	OR UCI
Age	First SCAD event < 47 YO (median) (188)	First SCAD event >= 47 YO (median) (196)	10	5.3	4	2	0.1	3	1	12
Arteriopathies	Have remote arteriopathy (112)	Do not have remote arteriopathy (272)	3	2.7	11	4	0.8	1	0	2.5
Hormones (females only)	Taken exogenous hormones (64)	Not taken exogenous hormones (295)	2	3.1	12	4.1	1	1	0	3.6
Hypermobility	Has hypermobility (27)	Does not have hypermobility (355)	3	11.1	11	3.1	0.1	4	1	16
Gravidy (females only)	Had >=1 pregnancy (312)	Had 0 pregnancy (48)	14	4.5	0	0	0.2	Inf	1	Inf
P-SCAD (females only)	Pregnancy associated SCAD (32)	Non-pregnancy associated SCAD (330)	0	0	14	4.2	0.6	0	0	3.1
Recurrence	>=2 SCAD events (40)	1 SCAD event (340)	0	0	14	4.1	0.4	0	0	2.6
Revascularisation	Revascularisation (128)	No Revascularisation (256)	3	2.3	11	4.3	0.4	1	0	2.1

Supplementary Table 4: There are no significant differences in clinical endpoints between SCAD survivors with a pathogenic or likely pathogenic variants and those without. Arteriopathy is defined as any arterial abnormality and may include dilations of arteries outside normal limits, dissections, aneurysms and fibromuscular dysplasia but does not include arterial tortuosity. Pregnancy-associated SCAD (P-SCAD) was defined as SCAD occurring during gestation or within 12 months of delivery. YO = years old. OR = Odds Ratio, LCI = Lower confidence interval (95%), UCI = Upper confidence interval (95%). Solved = has pathogenic or likely pathogenic variant. P values calculated using two-sided Fisher's Exact tests. P value threshold of significance for this analysis = 0.006 (0.05/8).

Supplementary Table 5

UK Biobank Field ID	Phenotypic category excluded from UK Biobank controls
20002	Self-declared cardiovascular
20002	Self-declared endocrine/diabetes
20002	Self-declared liver/biliary/pancreas problem
20002	Self-declared renal/urology
20002	Self-declared connective tissue disorder
41270, 40001, or 40002	ICD10 IV Endocrine, nutritional and metabolic diseases
41270, 40001, or 40002	ICD10 IX Diseases of the circulatory system
41270, 40001, or 40002	ICD10 XI Diseases of the digestive system > K70-K77 and K80-K87
41270, 40001, or 40002	ICD10 XII Diseases of the skin and subcutaneous tissue > L94
41270, 40001, or 40002	ICD10 XIII Diseases of the musculoskeletal system and connective tissue
41270, 40001, or 40002	ICD10 XIV Diseases of the genitourinary system > N00- N39
41270, 40001, or 40002	ICD10 XV Pregnancy, childbirth and the puerperium > O10-O16
41270, 40001, or 40002	ICD10 XVII Congenital malformations, deformations and chromosomal abnormalities > Q20-Q28, Q44- Q45, Q60-Q64, Q796, Q874, Q828
41270, 40001, or 40002	ICD10 XVIII Symptoms, signs and abnormal clinical and laboratory findings, not elsewhere classified > R00- R03, R30-R39, R80-R82, R943- R947, R96
41270, 40001, or 40002	ICD10 XXI Factors influencing health status and contact with health services > Z49, Z500, Z905, Z823, Z824, Z826, Z873
41271	ICD9 Complications Of Pregnancy, Childbirth, And The Puerperium > 642
41271	ICD9 Congenital Anomalies > 745-747,751,753, 75683, 75689,

	75982
41271	ICD9 Diseases Of The Circulatory System 390-459
41271	ICD9 Diseases Of The Digestive System > 570-579
41271	ICD9 Diseases Of The Genitourinary System > 580-599
41271	ICD9 Endocrine, Nutritional And Metabolic Diseases, And Immunity Disorders 240-278
41271	ICD9 Supplementary Classification Of Factors Influencing Health Status And Contact With Health Services > V56, V171, V173, V174, V1741, V1749, V177, V178, V1781, V1789
41271	ICD9 Symptoms, Signs, And Ill-Defined Conditions > 785,788,791,7943-7948
41271	ICD9 Diseases Of The Musculoskeletal System And Connective Tissue 710-739
Q2443	Medical conditions, Diabetes diagnosed by doctor = “Yes”
Q2966	Medical conditions, Age high blood pressure diagnosed, Age provided
Q2976	Medical conditions, Age diabetes diagnosed, Age provided
Q2986	Medical conditions, Started insulin within one year diagnosis of diabetes = “Yes”
Q3627	Medical conditions, Age angina diagnosed, Age provided
Q3894	Medical conditions, Age heart attack diagnosed, Age provided
Q4056	Medical conditions, Age stroke diagnosed, Age provided
Q6150_0	Medical conditions, Vascular/heart problems diagnosed by doctor = “Heart attack”, “Angina”, “Stroke”, or “High blood pressure”

Supplementary Table 5: Details of phenotypes excluded from UK Biobank controls for SCAD collapsing analysis.

Supplementary Table 6

**Gene Name**	**Model**	**Qual Cases**	**Qual Case Freq**	**Qual Ctrls**	**Qual Ctrl Freq**	**P value**	**Odds Ratio**	**Odds Ratio LCI**	**Odds Ratio UCI**	"Artery - Coronary" tissue >1.5 TPM in GTEx	"Artery - Coronary" tissue top decile expression in GTEx	**Gene flagged**
MUC21	Recessive	4	0.0112	2	1.46E-04	5.86E-06	77.7	14.2	425.8	.	.	TRUE
PKD1	Ultra-rare damaging (MTR)	6	0.0168	14	0.001	7.31E-06	16.7	6.4	43.8	Yes	Yes	FALSE
CGB5	Flexible non-syn	3	0.0084	0	0	1.62E-05	NA	NA	NA	.	.	TRUE
TBC1D9	Rare damaging (MTR)	7	0.0196	31	0.0023	4.07E-05	8.8	3.9	20.2	Yes	.	FALSE
TCEAL7	Flexible non-syn	4	0.0112	5	3.64E-04	4.63E-05	31.1	8.3	116.3	Yes	.	FALSE
DENND5A	Flexible non-syn	15	0.042	167	0.0122	6.24E-05	3.6	2.1	6.1	Yes	.	FALSE
KRTAP5-7	Flexible damaging	3	0.0084	1	7.29E-05	6.35E-05	116.3	12.1	1120.7	.	.	TRUE

ERC1	Flexible damaging	10	0.028	78	0.0057	7.55E-05	5	2.6	9.8	Yes	.	FALSE
CHRNA7	Flexible damaging	4	0.0112	6	4.37E-04	7.56E-05	25.9	7.3	92.2	.	.	FALSE
DUSP13	PTV	4	0.0112	7	5.10E-04	1.17E-04	22.2	6.5	76.2	.	.	FALSE
PRAMEF11	Flexible non-syn	7	0.0196	38	0.0028	1.26E-04	7.2	3.2	16.2	.	.	TRUE
CCL3	Flexible damaging	3	0.0084	2	1.46E-04	1.56E-04	58.1	9.7	349	Yes	.	TRUE
PRB4	PTV or rare damaging	3	0.0084	2	1.46E-04	1.56E-04	58.1	9.7	349	.	.	FALSE
IPO11	Ultra-rare damaging (MTR)	3	0.0084	2	1.46E-04	1.56E-04	58.1	9.7	349	Yes	.	FALSE
CLRN3	Flexible damaging	4	0.0112	8	5.83E-04	1.71E-04	19.4	5.8	64.8	.	.	FALSE
KRTAP5-1	Flexible non-syn (MTR)	5	0.014	17	0.0012	1.88E-04	11.5	4.2	31.2	.	.	TRUE
MBTPS1	Ultra-rare damaging	5	0.014	17	0.0012	1.88E-04	11.5	4.2	31.2	Yes	.	FALSE
PAM	Flexible damaging	9	0.0252	73	0.0053	2.25E-04	4.8	2.4	9.7	Yes	Yes	FALSE
COL3A1	Flexible non-syn (MTR)	7	0.0196	44	0.0032	2.82E-04	6.2	2.8	13.9	Yes	Yes	FALSE
HRCT1	Flexible damaging	0	0	337	0.0246	3.00E-04	0	NA	NA	Yes	.	FALSE
NBPF9	Flexible damaging	3	0.0084	3	2.19E-04	3.06E-04	38.8	7.8	192.7	Yes	.	TRUE
PHPT1	PTV	3	0.0084	3	2.19E-04	3.06E-04	38.8	7.8	192.7	Yes	Yes	FALSE

RABEP2	Ultra-rare damaging	3	0.0084	3	2.19E-04	3.06E-04	38.8	7.8	192.7	Yes	.	FALSE
GLB1L3	Rare damaging	8	0.0224	60	0.0044	3.10E-04	5.2	2.5	11	.	.	FALSE
UBE4B	Flexible non-syn	9	0.0252	77	0.0056	3.23E-04	4.6	2.3	9.2	Yes	.	FALSE
PGLYRP3	Flexible non-syn	9	0.0252	78	0.0057	3.52E-04	4.5	2.3	9.1	.	.	FALSE
EXTL1	Flexible damaging	9	0.0252	79	0.0058	3.84E-04	4.5	2.2	9	.	.	FALSE
OR2F2	Flexible non-syn	7	0.0196	47	0.0034	4.04E-04	5.8	2.6	13	.	.	TRUE
GOLGA6L2	Rare damaging (MTR)	5	0.014	21	0.0015	4.32E-04	9.3	3.5	24.7	.	.	TRUE
MNDA	Flexible non-syn (MTR)	4	0.0112	11	8.02E-04	4.45E-04	14.1	4.5	44.6	Yes	.	FALSE
RHOBTB3	Flexible non-syn (MTR)	4	0.0112	11	8.02E-04	4.45E-04	14.1	4.5	44.6	Yes	.	FALSE
PLEKHG3	Rare damaging (MTR)	4	0.0112	11	8.02E-04	4.45E-04	14.1	4.5	44.6	Yes	.	FALSE
ESX1	Rare damaging (MTR)	3	0.0084	4	2.92E-04	5.25E-04	29.1	6.5	130.3	.	.	FALSE
HARBI1	Rare damaging (MTR)	3	0.0084	4	2.92E-04	5.25E-04	29.1	6.5	130.3	.	.	FALSE
MAGEC1	Ultra-rare damaging	3	0.0084	4	2.92E-04	5.25E-04	29.1	6.5	130.3	.	.	FALSE
SERPINB12	Ultra-rare damaging	3	0.0084	4	2.92E-04	5.25E-04	29.1	6.5	130.3	.	.	FALSE
TLX3	Ultra-rare damaging	3	0.0084	4	2.92E-04	5.25E-04	29.1	6.5	130.3	.	.	FALSE
USP47	Ultra-rare damaging	3	0.0084	4	2.92E-04	5.25E-04	29.1	6.5	130.3	Yes	.	FALSE

	(MTR)											
EXOC2	Flexible non-syn	12	0.0336	141	0.0103	5.26E-04	3.4	1.8	6.1	Yes	.	FALSE
PCDHB14	Flexible non-syn	12	0.0336	141	0.0103	5.26E-04	3.4	1.8	6.1	Yes	.	FALSE
CLEC11A	Flexible damaging	4	0.0112	12	8.75E-04	5.81E-04	12.9	4.2	40.3	Yes	.	FALSE
RANBP2	Recessive	4	0.0112	12	8.75E-04	5.81E-04	12.9	4.2	40.3	Yes	.	FALSE
NPC1L1	Ultra-rare damaging	4	0.0112	12	8.75E-04	5.81E-04	12.9	4.2	40.3	.	.	FALSE
PXMP2	Rare damaging	5	0.014	23	0.0017	6.19E-04	8.5	3.2	22.4	Yes	.	FALSE
NBPF20	Flexible damaging	2	0.0056	0	0	6.41E-04	NA	NA	NA	.	.	TRUE
RPS13	Flexible damaging	2	0.0056	0	0	6.41E-04	NA	NA	NA	Yes	Yes	FALSE
GLI3	PTV	2	0.0056	0	0	6.41E-04	NA	NA	NA	Yes	.	FALSE
NFATC4	PTV	2	0.0056	0	0	6.41E-04	NA	NA	NA	Yes	Yes	FALSE
OLR1	PTV	2	0.0056	0	0	6.41E-04	NA	NA	NA	Yes	.	FALSE
PRKD2	PTV	2	0.0056	0	0	6.41E-04	NA	NA	NA	Yes	.	FALSE
KRTAP4-7	Rare damaging	2	0.0056	0	0	6.41E-04	NA	NA	NA	.	.	TRUE
FTMT	Rare damaging (MTR)	2	0.0056	0	0	6.41E-04	NA	NA	NA	.	.	FALSE
OGFOD3	Recessive	2	0.0056	0	0	6.41E-04	NA	NA	NA	Yes	.	FALSE

AZI2	Ultra-rare damaging (MTR)	2	0.0056	0	0	6.41E-04	NA	NA	NA	Yes	.	FALSE
ZBTB8OS	Ultra-rare damaging (MTR)	2	0.0056	0	0	6.41E-04	NA	NA	NA	Yes	.	FALSE
WWP2	PTV or rare damaging	6	0.0168	37	0.0027	7.03E-04	6.3	2.7	15.1	Yes	Yes	FALSE
SLC35B3	Flexible non-syn	8	0.0224	69	0.005	7.24E-04	4.5	2.2	9.5	Yes	.	FALSE
NECAB2	Flexible damaging	9	0.0252	87	0.0063	7.30E-04	4.1	2	8.1	.	.	FALSE
HOXC9	Flexible damaging	5	0.014	24	0.0017	7.33E-04	8.1	3.1	21.4	.	.	FALSE
ANXA5	Rare damaging	5	0.014	24	0.0017	7.33E-04	8.1	3.1	21.4	Yes	Yes	FALSE
CFAP221	Flexible non-syn	12	0.0336	147	0.0107	7.42E-04	3.2	1.8	5.8	.	.	FALSE
PLBD1	Flexible non-syn (MTR)	6	0.0168	38	0.0028	7.97E-04	6.2	2.6	14.7	Yes	.	FALSE
FAM186A	Rare damaging	6	0.0168	38	0.0028	7.97E-04	6.2	2.6	14.7	.	.	TRUE
METTL17	Ultra-rare damaging	3	0.0084	5	3.64E-04	8.24E-04	23.2	5.5	97.7	Yes	.	FALSE
SLC25A46	Ultra-rare damaging	3	0.0084	5	3.64E-04	8.24E-04	23.2	5.5	97.7	Yes	.	FALSE
PRPS1L1	Flexible non-syn	9	0.0252	89	0.0065	8.47E-04	4	2	7.9	.	.	TRUE
PLIN4	Flexible non-syn (MTR)	8	0.0224	71	0.0052	8.60E-04	4.4	2.1	9.2	Yes	Yes	FALSE

DOCK6	Recessive	5	0.014	25	0.0018	8.62E-04	7.8	3	20.4	Yes	.	FALSE
CFHR2	Flexible non-syn	6	0.0168	39	0.0028	9.01E-04	6	2.5	14.3	.	.	FALSE
KRT82	Rare damaging	6	0.0168	39	0.0028	9.01E-04	6	2.5	14.3	.	.	TRUE
LRRC37B	PTV or rare damaging	4	0.0112	14	0.001	9.38E-04	11.1	3.6	33.9	Yes	.	FALSE
FABP7	Flexible non-syn	5	0.014	26	0.0019	0.001	7.5	2.9	19.6	.	.	FALSE
SPI1	Flexible non-syn	5	0.014	26	0.0019	0.001	7.5	2.9	19.6	Yes	.	FALSE
SLC25A44	Flexible damaging	6	0.0168	41	0.003	0.0011	5.7	2.4	13.5	Yes	.	FALSE
TMCO5A	Flexible non-syn	5	0.014	27	0.002	0.0012	7.2	2.8	18.8	.	.	FALSE
FH	Ultra-rare damaging	4	0.0112	15	0.0011	0.0012	10.4	3.4	31.4	Yes	.	FALSE
NAT1	Flexible damaging	3	0.0084	6	4.37E-04	0.0012	19.4	4.8	77.8	.	.	FALSE
CCZ1B	Flexible non-syn	3	0.0084	6	4.37E-04	0.0012	19.4	4.8	77.8	Yes	.	TRUE
PLCH1	PTV	3	0.0084	6	4.37E-04	0.0012	19.4	4.8	77.8	.	.	FALSE
SERPINB9	Rare damaging (MTR)	3	0.0084	6	4.37E-04	0.0012	19.4	4.8	77.8	Yes	.	FALSE
SMC6	Rare damaging (MTR)	3	0.0084	6	4.37E-04	0.0012	19.4	4.8	77.8	Yes	.	FALSE
IQUB	Recessive	3	0.0084	6	4.37E-04	0.0012	19.4	4.8	77.8	.	.	FALSE
ZNF140	Flexible non-syn	6	0.0168	43	0.0031	0.0014	5.4	2.3	12.9	Yes	.	FALSE

MFSD10	Flexible non-syn (MTR)	6	0.0168	43	0.0031	0.0014	5.4	2.3	12.9	Yes	.	FALSE
KCNMB4	Flexible non-syn	4	0.0112	16	0.0012	0.0014	9.7	3.2	29.2	Yes	.	FALSE
CDKL1	Rare damaging	4	0.0112	16	0.0012	0.0014	9.7	3.2	29.2	.	.	FALSE
ZNRF3	Ultra-rare damaging	4	0.0112	16	0.0012	0.0014	9.7	3.2	29.2	Yes	.	FALSE
NDUFS1	Flexible non-syn	11	0.0308	138	0.0101	0.0015	3.1	1.7	5.8	Yes	.	FALSE
SENP3	Flexible non-syn	8	0.0224	78	0.0057	0.0015	4	1.9	8.4	Yes	.	FALSE
DNAH10	Ultra-rare damaging	8	0.0224	78	0.0057	0.0015	4	1.9	8.4	.	.	FALSE
POFUT2	Flexible non-syn (MTR)	5	0.014	29	0.0021	0.0015	6.7	2.6	17.4	Yes	.	FALSE
NLRP9	Flexible non-syn	11	0.0308	141	0.0103	0.0017	3.1	1.6	5.7	.	.	FALSE
GPD2	Flexible damaging	8	0.0224	80	0.0058	0.0017	3.9	1.9	8.1	Yes	.	FALSE
RIMS3	Flexible damaging	4	0.0112	17	0.0012	0.0017	9.1	3.1	27.3	Yes	.	FALSE
NT5C3B	PTV or rare damaging	4	0.0112	17	0.0012	0.0017	9.1	3.1	27.3	Yes	.	FALSE
AARS	Ultra-rare damaging	4	0.0112	17	0.0012	0.0017	9.1	3.1	27.3	Yes	Yes	FALSE
FHOD1	Ultra-rare damaging	4	0.0112	17	0.0012	0.0017	9.1	3.1	27.3	Yes	.	FALSE
SEC24B	Ultra-rare damaging	4	0.0112	17	0.0012	0.0017	9.1	3.1	27.3	Yes	.	FALSE
LYPD1	Flexible damaging	3	0.0084	7	5.10E-04	0.0017	16.6	4.3	64.5	.	.	FALSE

DNAJC24	Flexible non-syn	3	0.0084	7	5.10E-04	0.0017	16.6	4.3	64.5	Yes	.	FALSE
NUS1	Flexible non-syn (MTR)	3	0.0084	7	5.10E-04	0.0017	16.6	4.3	64.5	Yes	.	FALSE
G3BP2	PTV or rare damaging	3	0.0084	7	5.10E-04	0.0017	16.6	4.3	64.5	Yes	.	FALSE
CDADC1	Rare damaging (MTR)	3	0.0084	7	5.10E-04	0.0017	16.6	4.3	64.5	Yes	.	FALSE
SLC25A38	Rare damaging (MTR)	3	0.0084	7	5.10E-04	0.0017	16.6	4.3	64.5	Yes	.	FALSE
HUNK	Ultra-rare damaging	3	0.0084	7	5.10E-04	0.0017	16.6	4.3	64.5	.	.	FALSE
PRG4	Ultra-rare damaging (MTR)	3	0.0084	7	5.10E-04	0.0017	16.6	4.3	64.5	Yes	.	FALSE
HMGCS2	Flexible non-syn	9	0.0252	100	0.0073	0.0018	3.5	1.8	7	.	.	FALSE
NENF	Flexible non-syn	5	0.014	30	0.0022	0.0018	6.5	2.5	16.8	Yes	Yes	FALSE
THADA	Flexible damaging	9	0.0252	101	0.0074	0.0019	3.5	1.7	7	Yes	.	FALSE
ADAM2	Flexible non-syn	9	0.0252	101	0.0074	0.0019	3.5	1.7	7	.	.	FALSE
SMARCA1	Flexible non-syn	6	0.0168	46	0.0034	0.0019	5.1	2.2	12	Yes	.	FALSE
CCL17	Flexible damaging	2	0.0056	1	7.29E-05	0.0019	77.3	7	854.5	.	.	FALSE
DEDD	Flexible damaging	2	0.0056	1	7.29E-05	0.0019	77.3	7	854.5	Yes	.	FALSE
LSM2	Flexible damaging	2	0.0056	1	7.29E-05	0.0019	77.3	7	854.5	Yes	.	FALSE

PDHA2	Flexible non-syn (MTR)	2	0.0056	1	7.29E-05	0.0019	77.3	7	854.5	.	.	FALSE
ADGRB2	PTV	2	0.0056	1	7.29E-05	0.0019	77.3	7	854.5	.	.	FALSE
PCDHGC4	PTV	2	0.0056	1	7.29E-05	0.0019	77.3	7	854.5	.	.	FALSE
IL17A	Rare damaging (MTR)	2	0.0056	1	7.29E-05	0.0019	77.3	7	854.5	.	.	FALSE
NCK1	Rare damaging (MTR)	2	0.0056	1	7.29E-05	0.0019	77.3	7	854.5	Yes	.	FALSE
PTPRN2	Recessive	2	0.0056	1	7.29E-05	0.0019	77.3	7	854.5	.	.	FALSE
CDRT4	Ultra-rare damaging	2	0.0056	1	7.29E-05	0.0019	77.3	7	854.5	Yes	.	FALSE
TFB2M	Ultra-rare damaging	2	0.0056	1	7.29E-05	0.0019	77.3	7	854.5	Yes	.	FALSE
ZNF343	Ultra-rare damaging	2	0.0056	1	7.29E-05	0.0019	77.3	7	854.5	Yes	.	FALSE
B3GLCT	Ultra-rare damaging (MTR)	2	0.0056	1	7.29E-05	0.0019	77.3	7	854.5	Yes	.	FALSE
ZNF45	Ultra-rare damaging (MTR)	2	0.0056	1	7.29E-05	0.0019	77.3	7	854.5	Yes	.	FALSE
SRCAP	PTV or rare damaging	14	0.0392	213	0.0155	0.002	2.6	1.5	4.5	Yes	.	TRUE
ARRDC1	Flexible damaging	5	0.014	31	0.0023	0.002	6.3	2.4	16.2	Yes	.	FALSE
C16orf95	Flexible non-syn	5	0.014	31	0.0023	0.002	6.3	2.4	16.2	.	.	FALSE

AP4E1	Rare damaging	5	0.014	31	0.0023	0.002	6.3	2.4	16.2	Yes	.	FALSE
DENND3	Flexible non-syn	13	0.0364	191	0.0139	0.0021	2.7	1.5	4.7	Yes	.	FALSE
MPP7	Flexible damaging	6	0.0168	47	0.0034	0.0021	5	2.1	11.7	Yes	.	FALSE
PRX	Flexible damaging	4	0.0112	18	0.0013	0.0021	8.6	2.9	25.6	Yes	.	FALSE
USP17L7	Flexible damaging	4	0.0112	18	0.0013	0.0021	8.6	2.9	25.6	.	.	TRUE
IPO7	PTV or rare damaging	4	0.0112	18	0.0013	0.0021	8.6	2.9	25.6	Yes	.	FALSE
BCL9L	Flexible non-syn	13	0.0364	193	0.0141	0.0023	2.6	1.5	4.7	Yes	.	FALSE
ZNF154	Flexible non-syn	9	0.0252	104	0.0076	0.0023	3.4	1.7	6.7	Yes	.	FALSE
CFHR4	Flexible damaging	5	0.014	32	0.0023	0.0023	6.1	2.4	15.7	.	.	FALSE
RGPD3	Flexible non-syn	3	0.0084	8	5.83E-04	0.0023	14.5	3.8	55	.	.	TRUE
IDO2	Flexible non-syn (MTR)	3	0.0084	8	5.83E-04	0.0023	14.5	3.8	55	.	.	FALSE
SLC2A5	PTV	3	0.0084	8	5.83E-04	0.0023	14.5	3.8	55	Yes	.	FALSE
CCNB1	Ultra-rare damaging	3	0.0084	8	5.83E-04	0.0023	14.5	3.8	55	.	.	FALSE
TMEM209	Ultra-rare damaging	3	0.0084	8	5.83E-04	0.0023	14.5	3.8	55	Yes	.	FALSE
ZBTB48	Ultra-rare damaging	3	0.0084	8	5.83E-04	0.0023	14.5	3.8	55	Yes	.	FALSE
ZNF578	Flexible non-syn	11	0.0308	148	0.0108	0.0024	2.9	1.6	5.4	.	.	FALSE

ARSH	Flexible damaging	9	0.0252	105	0.0077	0.0025	3.4	1.7	6.7	.	.	FALSE
ACTL6B	Flexible non-syn (MTR)	4	0.0112	19	0.0014	0.0025	8.2	2.8	24.1	.	.	FALSE
SETD2	Recessive	4	0.0112	19	0.0014	0.0025	8.2	2.8	24.1	Yes	.	FALSE
ANKIB1	Flexible non-syn	8	0.0224	86	0.0063	0.0026	3.6	1.7	7.6	Yes	.	TRUE
OCM	Flexible non-syn	6	0.0168	49	0.0036	0.0026	4.8	2	11.2	.	.	TRUE
ALAS1	PTV or rare damaging	6	0.0168	49	0.0036	0.0026	4.8	2	11.2	Yes	.	FALSE
BACH2	Flexible non-syn (MTR)	5	0.014	33	0.0024	0.0026	5.9	2.3	15.2	.	.	FALSE
PUM1	Flexible non-syn (MTR)	5	0.014	33	0.0024	0.0026	5.9	2.3	15.2	Yes	.	FALSE
EHMT2	PTV or rare damaging	5	0.014	33	0.0024	0.0026	5.9	2.3	15.2	Yes	.	FALSE
NOP58	PTV or rare damaging	5	0.014	33	0.0024	0.0026	5.9	2.3	15.2	Yes	.	FALSE
SCFD2	Flexible non-syn	10	0.028	128	0.0093	0.0027	3.1	1.6	5.9	Yes	.	FALSE
BRD7	Flexible non-syn	8	0.0224	87	0.0063	0.0028	3.6	1.7	7.5	Yes	.	FALSE
YTHDC1	Flexible non-syn	8	0.0224	87	0.0063	0.0028	3.6	1.7	7.5	Yes	.	FALSE
DNAJB1	Flexible non-syn	7	0.0196	68	0.005	0.0029	4	1.8	8.8	Yes	Yes	FALSE
PGD	Flexible damaging	5	0.014	34	0.0025	0.0029	5.7	2.2	14.7	Yes	Yes	FALSE
GPR22	Flexible non-syn	5	0.014	34	0.0025	0.0029	5.7	2.2	14.7	.	.	FALSE

NDRG4	Flexible non-syn (MTR)	4	0.0112	20	0.0015	0.0029	7.8	2.6	22.8	Yes	.	FALSE
PCDHA5	Flexible non-syn (MTR)	4	0.0112	20	0.0015	0.0029	7.8	2.6	22.8	.	.	FALSE
SLC39A12	Flexible non-syn (MTR)	4	0.0112	20	0.0015	0.0029	7.8	2.6	22.8	.	.	FALSE
SRC	Flexible non-syn (MTR)	4	0.0112	20	0.0015	0.0029	7.8	2.6	22.8	Yes	.	FALSE
HS1BP3	Flexible non-syn (MTR)	3	0.0084	9	6.56E-04	0.003	12.9	3.5	47.9	Yes	.	FALSE
PSMB7	PTV or rare damaging	3	0.0084	9	6.56E-04	0.003	12.9	3.5	47.9	Yes	Yes	FALSE
SLC6A16	Ultra-rare damaging	3	0.0084	9	6.56E-04	0.003	12.9	3.5	47.9	Yes	.	FALSE
TRDMT1	Ultra-rare damaging	3	0.0084	9	6.56E-04	0.003	12.9	3.5	47.9	Yes	.	FALSE
XPO4	Ultra-rare damaging	3	0.0084	9	6.56E-04	0.003	12.9	3.5	47.9	Yes	.	FALSE
OR51S1	Flexible non-syn	7	0.0196	69	0.005	0.0031	4	1.8	8.7	.	.	TRUE
AFG3L2	Flexible damaging	6	0.0168	51	0.0037	0.0031	4.6	2	10.7	Yes	.	FALSE
UNC80	Flexible non-syn (MTR)	8	0.0224	89	0.0065	0.0032	3.5	1.7	7.3	.	.	FALSE
ACSS2	Flexible damaging	8	0.0224	90	0.0066	0.0034	3.5	1.7	7.2	Yes	.	FALSE
TEX11	Flexible non-syn	8	0.0224	90	0.0066	0.0034	3.5	1.7	7.2	.	.	FALSE
ARHGAP22	Flexible damaging	6	0.0168	52	0.0038	0.0034	4.5	1.9	10.5	Yes	.	FALSE
BRICD5	Flexible damaging	6	0.0168	52	0.0038	0.0034	4.5	1.9	10.5	Yes	.	FALSE

AP3D1	PTV or rare damaging	6	0.0168	52	0.0038	0.0034	4.5	1.9	10.5	Yes	.	FALSE
METTL2B	Flexible non-syn	4	0.0112	21	0.0015	0.0034	7.4	2.5	21.6	Yes	.	TRUE
GRHL1	PTV or rare damaging	4	0.0112	21	0.0015	0.0034	7.4	2.5	21.6	.	.	FALSE
DNAH2	Ultra-rare damaging (MTR)	4	0.0112	21	0.0015	0.0034	7.4	2.5	21.6	.	.	FALSE
SYT8	Flexible non-syn	10	0.028	133	0.0097	0.0035	2.9	1.5	5.6	.	.	FALSE
SEMG1	Flexible non-syn	8	0.0224	91	0.0066	0.0036	3.4	1.7	7.1	.	.	FALSE
SGO1	Flexible non-syn	8	0.0224	91	0.0066	0.0036	3.4	1.7	7.1	.	.	FALSE
ACOXL	PTV or rare damaging	5	0.014	36	0.0026	0.0036	5.4	2.1	13.8	.	.	FALSE
DGUOK	Flexible damaging	6	0.0168	53	0.0039	0.0037	4.4	1.9	10.3	Yes	.	FALSE
COMMD10	Flexible damaging	2	0.0056	2	1.46E-04	0.0037	38.6	5.4	275.2	Yes	.	FALSE
CBX3	Flexible non-syn (MTR)	2	0.0056	2	1.46E-04	0.0037	38.6	5.4	275.2	Yes	Yes	FALSE
HEPN1	Flexible non-syn (MTR)	2	0.0056	2	1.46E-04	0.0037	38.6	5.4	275.2	Yes	.	FALSE
TRAPPC2	Flexible non-syn (MTR)	2	0.0056	2	1.46E-04	0.0037	38.6	5.4	275.2	Yes	.	FALSE
C6orf163	PTV	2	0.0056	2	1.46E-04	0.0037	38.6	5.4	275.2	.	.	FALSE
SH3BP4	PTV	2	0.0056	2	1.46E-04	0.0037	38.6	5.4	275.2	Yes	.	FALSE

SYNJ1	PTV	2	0.0056	2	1.46E-04	0.0037	38.6	5.4	275.2	Yes	.	FALSE
TMEM8B	PTV	2	0.0056	2	1.46E-04	0.0037	38.6	5.4	275.2	Yes	.	FALSE
TTC32	PTV	2	0.0056	2	1.46E-04	0.0037	38.6	5.4	275.2	Yes	.	FALSE
ZNF606	PTV or rare damaging	2	0.0056	2	1.46E-04	0.0037	38.6	5.4	275.2	Yes	.	FALSE
ARHGAP25	Rare damaging (MTR)	2	0.0056	2	1.46E-04	0.0037	38.6	5.4	275.2	Yes	.	FALSE
CTDSP2	Rare damaging (MTR)	2	0.0056	2	1.46E-04	0.0037	38.6	5.4	275.2	Yes	Yes	FALSE
HES5	Rare damaging (MTR)	2	0.0056	2	1.46E-04	0.0037	38.6	5.4	275.2	.	.	FALSE
MLLT3	Rare damaging (MTR)	2	0.0056	2	1.46E-04	0.0037	38.6	5.4	275.2	.	.	FALSE
SLC17A3	Rare damaging (MTR)	2	0.0056	2	1.46E-04	0.0037	38.6	5.4	275.2	.	.	FALSE
ZNF880	Recessive	2	0.0056	2	1.46E-04	0.0037	38.6	5.4	275.2	Yes	.	FALSE
MYL9	Ultra-rare damaging	2	0.0056	2	1.46E-04	0.0037	38.6	5.4	275.2	Yes	Yes	FALSE
PCGF2	Ultra-rare damaging	2	0.0056	2	1.46E-04	0.0037	38.6	5.4	275.2	Yes	.	FALSE
TRMO	Ultra-rare damaging	2	0.0056	2	1.46E-04	0.0037	38.6	5.4	275.2	Yes	.	FALSE
RRP8	Ultra-rare damaging (MTR)	2	0.0056	2	1.46E-04	0.0037	38.6	5.4	275.2	Yes	.	FALSE
TMEM165	Ultra-rare damaging	2	0.0056	2	1.46E-04	0.0037	38.6	5.4	275.2	Yes	Yes	FALSE

	(MTR)											
UTP4	Ultra-rare damaging (MTR)	2	0.0056	2	1.46E-04	0.0037	38.6	5.4	275.2	Yes	.	FALSE
VPS28	Ultra-rare damaging (MTR)	2	0.0056	2	1.46E-04	0.0037	38.6	5.4	275.2	Yes	Yes	FALSE
DNAH6	Flexible non-syn	30	0.084	657	0.0479	0.0038	1.8	1.2	2.7	.	.	FALSE
KIAA1210	Flexible damaging	3	0.0084	10	7.29E-04	0.0038	11.6	3.2	42.4	.	.	FALSE
TSLP	Flexible damaging	3	0.0084	10	7.29E-04	0.0038	11.6	3.2	42.4	.	.	FALSE
KRT77	PTV	3	0.0084	10	7.29E-04	0.0038	11.6	3.2	42.4	.	.	TRUE
NKX1-2	Rare damaging	3	0.0084	10	7.29E-04	0.0038	11.6	3.2	42.4	.	.	FALSE
FECH	Rare damaging (MTR)	3	0.0084	10	7.29E-04	0.0038	11.6	3.2	42.4	Yes	.	FALSE
F2	Ultra-rare damaging	3	0.0084	10	7.29E-04	0.0038	11.6	3.2	42.4	.	.	FALSE
RALGAPA1	PTV or rare damaging	8	0.0224	92	0.0067	0.0039	3.4	1.6	7	Yes	.	FALSE
HDAC9	Flexible damaging	4	0.0112	22	0.0016	0.0039	7.1	2.4	20.6	Yes	.	FALSE
PLEKHA1	Rare damaging	4	0.0112	22	0.0016	0.0039	7.1	2.4	20.6	Yes	.	FALSE
IRF3	Rare damaging (MTR)	4	0.0112	22	0.0016	0.0039	7.1	2.4	20.6	Yes	.	FALSE

GLRB	Flexible damaging	6	0.0168	54	0.0039	0.004	4.3	1.8	10.1	Yes	.	FALSE
ZSCAN16	Flexible non-syn	5	0.014	37	0.0027	0.004	5.3	2.1	13.4	Yes	.	FALSE
AURKB	Flexible non-syn	8	0.0224	93	0.0068	0.0041	3.4	1.6	7	.	.	FALSE
SH3KBP1	Flexible non-syn	7	0.0196	73	0.0053	0.0041	3.7	1.7	8.2	Yes	.	FALSE
KRT10	PTV or rare damaging	7	0.0196	73	0.0053	0.0041	3.7	1.7	8.2	Yes	Yes	TRUE
CDHR4	Flexible non-syn	11	0.0308	160	0.0117	0.0043	2.7	1.4	5	.	.	FALSE
ZNF142	Flexible damaging	7	0.0196	74	0.0054	0.0044	3.7	1.7	8.1	Yes	.	FALSE
SEC23A	Flexible non-syn	7	0.0196	74	0.0054	0.0044	3.7	1.7	8.1	Yes	.	FALSE
CLCA4	PTV or rare damaging	7	0.0196	74	0.0054	0.0044	3.7	1.7	8.1	.	.	FALSE
PLXNB1	Flexible non-syn	15	0.042	253	0.0184	0.0045	2.3	1.4	4	Yes	Yes	FALSE
KRTAP21-3	Flexible non-syn	4	0.0112	23	0.0017	0.0045	6.7	2.3	19.6	.	.	TRUE
SNN	Flexible non-syn	4	0.0112	23	0.0017	0.0045	6.7	2.3	19.6	Yes	.	FALSE
ASB16	Flexible non-syn (MTR)	4	0.0112	23	0.0017	0.0045	6.7	2.3	19.6	.	.	FALSE
ASPHD2	Flexible non-syn (MTR)	4	0.0112	23	0.0017	0.0045	6.7	2.3	19.6	.	.	FALSE
SNX31	Flexible non-syn (MTR)	4	0.0112	23	0.0017	0.0045	6.7	2.3	19.6	.	.	FALSE
RASGRP3	Flexible non-syn	8	0.0224	95	0.0069	0.0046	3.3	1.6	6.8	Yes	.	FALSE

KIF21A	PTV or rare damaging	8	0.0224	95	0.0069	0.0046	3.3	1.6	6.8	Yes	.	FALSE
PCNT	Flexible non-syn	33	0.0924	753	0.0549	0.0047	1.8	1.2	2.5	Yes	.	FALSE
SCG3	Flexible non-syn	6	0.0168	56	0.0041	0.0047	4.2	1.8	9.7	.	.	FALSE
KCNS3	PTV or rare damaging	6	0.0168	56	0.0041	0.0047	4.2	1.8	9.7	Yes	.	FALSE
PIK3C2G	Rare damaging	6	0.0168	56	0.0041	0.0047	4.2	1.8	9.7	.	.	FALSE
COL18A1	Ultra-rare damaging	6	0.0168	56	0.0041	0.0047	4.2	1.8	9.7	Yes	Yes	FALSE
TRIB3	Flexible non-syn	9	0.0252	117	0.0085	0.0048	3	1.5	6	Yes	.	TRUE
GAS2L1	Flexible damaging	3	0.0084	11	8.02E-04	0.0048	10.6	2.9	38	Yes	.	FALSE
LEO1	Flexible damaging	3	0.0084	11	8.02E-04	0.0048	10.6	2.9	38	Yes	.	FALSE
ADSL	Flexible non-syn (MTR)	3	0.0084	11	8.02E-04	0.0048	10.6	2.9	38	Yes	.	FALSE
CTSG	Flexible non-syn (MTR)	3	0.0084	11	8.02E-04	0.0048	10.6	2.9	38	Yes	.	FALSE
GSG1	Flexible non-syn (MTR)	3	0.0084	11	8.02E-04	0.0048	10.6	2.9	38	.	.	FALSE
MKRN1	Flexible non-syn (MTR)	3	0.0084	11	8.02E-04	0.0048	10.6	2.9	38	Yes	.	FALSE
ONECUT3	Flexible non-syn (MTR)	3	0.0084	11	8.02E-04	0.0048	10.6	2.9	38	.	.	FALSE
PRELID3A	Flexible non-syn (MTR)	3	0.0084	11	8.02E-04	0.0048	10.6	2.9	38	Yes	.	FALSE
SSMEM1	Flexible non-syn (MTR)	3	0.0084	11	8.02E-04	0.0048	10.6	2.9	38	.	.	FALSE

ZC3H15	Flexible non-syn (MTR)	3	0.0084	11	8.02E-04	0.0048	10.6	2.9	38	Yes	.	FALSE
KARS	Rare damaging (MTR)	3	0.0084	11	8.02E-04	0.0048	10.6	2.9	38	Yes	Yes	FALSE
RNF19A	Rare damaging (MTR)	3	0.0084	11	8.02E-04	0.0048	10.6	2.9	38	Yes	.	FALSE
SLC1A3	Rare damaging (MTR)	3	0.0084	11	8.02E-04	0.0048	10.6	2.9	38	Yes	.	FALSE
AP3M2	Ultra-rare damaging	3	0.0084	11	8.02E-04	0.0048	10.6	2.9	38	Yes	.	FALSE
SLC7A6	Ultra-rare damaging	3	0.0084	11	8.02E-04	0.0048	10.6	2.9	38	Yes	.	FALSE
CNKSR2	Flexible non-syn	5	0.014	39	0.0028	0.0049	5	2	12.7	.	.	FALSE
ZNF224	Flexible non-syn (MTR)	5	0.014	39	0.0028	0.0049	5	2	12.7	Yes	.	FALSE
GRXCR2	Flexible non-syn	7	0.0196	76	0.0055	0.0051	3.6	1.6	7.8	.	.	FALSE
TMEM74	Flexible non-syn	6	0.0168	57	0.0042	0.0051	4.1	1.8	9.6	.	.	FALSE
GPX2	Flexible damaging	4	0.0112	24	0.0017	0.0051	6.5	2.2	18.7	.	.	FALSE
CDK5RAP3	Flexible non-syn (MTR)	4	0.0112	24	0.0017	0.0051	6.5	2.2	18.7	Yes	Yes	FALSE
DCLK3	Flexible non-syn (MTR)	4	0.0112	24	0.0017	0.0051	6.5	2.2	18.7	.	.	FALSE
FBXO11	Flexible non-syn (MTR)	4	0.0112	24	0.0017	0.0051	6.5	2.2	18.7	Yes	.	FALSE
LITAF	Flexible non-syn	5	0.014	40	0.0029	0.0054	4.9	1.9	12.4	Yes	Yes	FALSE
STPG1	Flexible non-syn	5	0.014	40	0.0029	0.0054	4.9	1.9	12.4	Yes	.	FALSE

CALHM6	Flexible non-syn	6	0.0168	58	0.0042	0.0055	4	1.7	9.4	Yes	.	FALSE
KIAA1217	Flexible non-syn	18	0.0504	340	0.0248	0.0056	2.1	1.3	3.4	Yes	.	FALSE
SIGLEC1	Flexible damaging	10	0.028	143	0.0104	0.0056	2.7	1.4	5.2	Yes	.	FALSE
HIF3A	Flexible non-syn	10	0.028	143	0.0104	0.0056	2.7	1.4	5.2	Yes	.	FALSE
ANKRD27	Flexible non-syn	14	0.0392	232	0.0169	0.0058	2.4	1.4	4.1	Yes	.	TRUE
BAIAP3	Rare damaging	8	0.0224	99	0.0072	0.0058	3.2	1.5	6.5	.	.	FALSE
LRRC40	Flexible non-syn	7	0.0196	78	0.0057	0.0058	3.5	1.6	7.6	Yes	.	FALSE
FBXW10	Flexible non-syn	4	0.0112	25	0.0018	0.0058	6.2	2.1	17.9	.	.	FALSE
ANKS1B	Flexible non-syn (MTR)	4	0.0112	25	0.0018	0.0058	6.2	2.1	17.9	.	.	TRUE
PLEKHG2	Flexible non-syn	17	0.0476	306	0.0223	0.0059	2.2	1.3	3.6	Yes	.	FALSE
AKAP11	Flexible non-syn	12	0.0336	192	0.014	0.0059	2.5	1.4	4.4	Yes	.	FALSE
CYP1B1	Flexible non-syn	9	0.0252	121	0.0088	0.0059	2.9	1.5	5.8	Yes	.	FALSE
IFNE	Flexible damaging	3	0.0084	12	8.75E-04	0.0059	9.7	2.7	34.5	.	.	FALSE
ZNF408	Flexible damaging	3	0.0084	12	8.75E-04	0.0059	9.7	2.7	34.5	Yes	.	FALSE
AKAP10	Flexible non-syn (MTR)	3	0.0084	12	8.75E-04	0.0059	9.7	2.7	34.5	Yes	.	FALSE
NAP1L2	Flexible non-syn (MTR)	3	0.0084	12	8.75E-04	0.0059	9.7	2.7	34.5	Yes	.	FALSE

ARRDC4	PTV or rare damaging	3	0.0084	12	8.75E-04	0.0059	9.7	2.7	34.5	Yes	.	FALSE
NUDT22	Rare damaging	3	0.0084	12	8.75E-04	0.0059	9.7	2.7	34.5	Yes	.	FALSE
ALDOA	Ultra-rare damaging	3	0.0084	12	8.75E-04	0.0059	9.7	2.7	34.5	Yes	Yes	FALSE
C8A	Ultra-rare damaging	3	0.0084	12	8.75E-04	0.0059	9.7	2.7	34.5	.	.	FALSE
NUDT12	Flexible non-syn	6	0.0168	59	0.0043	0.006	4	1.7	9.2	Yes	.	FALSE
STAC	Flexible non-syn	6	0.0168	59	0.0043	0.006	4	1.7	9.2	Yes	.	FALSE
RNF175	Flexible damaging	5	0.014	41	0.003	0.006	4.7	1.9	12.1	.	.	FALSE
ABTB1	PTV or rare damaging	5	0.014	41	0.003	0.006	4.7	1.9	12.1	Yes	.	FALSE
NOL3	PTV or rare damaging	5	0.014	41	0.003	0.006	4.7	1.9	12.1	Yes	.	FALSE
IGFN1	Rare damaging (MTR)	5	0.014	41	0.003	0.006	4.7	1.9	12.1	.	.	FALSE
ITPR1	Flexible non-syn	12	0.0336	193	0.0141	0.0061	2.4	1.3	4.4	Yes	.	FALSE
MCM7	Flexible non-syn	12	0.0336	193	0.0141	0.0061	2.4	1.3	4.4	Yes	.	FALSE
MTHFR	Recessive	7	0.0196	79	0.0058	0.0061	3.5	1.6	7.5	Yes	.	FALSE
FAM25A	Flexible damaging	2	0.0056	3	2.19E-04	0.0061	25.8	4.3	154.7	.	.	TRUE
MUC7	Flexible damaging	2	0.0056	3	2.19E-04	0.0061	25.8	4.3	154.7	.	.	TRUE
SMIM9	Flexible damaging	2	0.0056	3	2.19E-04	0.0061	25.8	4.3	154.7	.	.	FALSE

GTPBP10	Flexible non-syn (MTR)	2	0.0056	3	2.19E-04	0.0061	25.8	4.3	154.7	Yes	.	FALSE
PCDHB8	Flexible non-syn (MTR)	2	0.0056	3	2.19E-04	0.0061	25.8	4.3	154.7	.	.	FALSE
STYX	Flexible non-syn (MTR)	2	0.0056	3	2.19E-04	0.0061	25.8	4.3	154.7	Yes	.	FALSE
BTLA	PTV	2	0.0056	3	2.19E-04	0.0061	25.8	4.3	154.7	.	.	FALSE
LYZ	PTV	2	0.0056	3	2.19E-04	0.0061	25.8	4.3	154.7	Yes	Yes	FALSE
MCMBP	PTV	2	0.0056	3	2.19E-04	0.0061	25.8	4.3	154.7	Yes	.	FALSE
PPOX	PTV	2	0.0056	3	2.19E-04	0.0061	25.8	4.3	154.7	Yes	.	FALSE
RCAN3	PTV	2	0.0056	3	2.19E-04	0.0061	25.8	4.3	154.7	.	.	FALSE
ZBTB10	PTV	2	0.0056	3	2.19E-04	0.0061	25.8	4.3	154.7	Yes	.	FALSE
ZNF789	Rare damaging	2	0.0056	3	2.19E-04	0.0061	25.8	4.3	154.7	Yes	.	FALSE
ACSM2B	Rare damaging (MTR)	2	0.0056	3	2.19E-04	0.0061	25.8	4.3	154.7	.	.	TRUE
C3orf20	Rare damaging (MTR)	2	0.0056	3	2.19E-04	0.0061	25.8	4.3	154.7	.	.	FALSE
LRRC1	Rare damaging (MTR)	2	0.0056	3	2.19E-04	0.0061	25.8	4.3	154.7	Yes	.	FALSE
PSD3	Rare damaging (MTR)	2	0.0056	3	2.19E-04	0.0061	25.8	4.3	154.7	Yes	.	FALSE
PCDHA4	Recessive	2	0.0056	3	2.19E-04	0.0061	25.8	4.3	154.7	.	.	FALSE
SOX30	Recessive	2	0.0056	3	2.19E-04	0.0061	25.8	4.3	154.7	.	.	FALSE

BUD13	Ultra-rare damaging	2	0.0056	3	2.19E-04	0.0061	25.8	4.3	154.7	Yes	.	FALSE
FAF2	Ultra-rare damaging	2	0.0056	3	2.19E-04	0.0061	25.8	4.3	154.7	Yes	.	FALSE
FAM83A	Ultra-rare damaging	2	0.0056	3	2.19E-04	0.0061	25.8	4.3	154.7	.	.	TRUE
GAB2	Ultra-rare damaging	2	0.0056	3	2.19E-04	0.0061	25.8	4.3	154.7	Yes	.	FALSE
RNFT1	Ultra-rare damaging	2	0.0056	3	2.19E-04	0.0061	25.8	4.3	154.7	Yes	.	FALSE
SLC25A26	Ultra-rare damaging	2	0.0056	3	2.19E-04	0.0061	25.8	4.3	154.7	Yes	.	FALSE
ARNTL	Ultra-rare damaging (MTR)	2	0.0056	3	2.19E-04	0.0061	25.8	4.3	154.7	Yes	.	FALSE
B3GAT1	Ultra-rare damaging (MTR)	2	0.0056	3	2.19E-04	0.0061	25.8	4.3	154.7	.	.	FALSE
CACNA2D1	Ultra-rare damaging (MTR)	2	0.0056	3	2.19E-04	0.0061	25.8	4.3	154.7	Yes	.	FALSE
FCGRT	Ultra-rare damaging (MTR)	2	0.0056	3	2.19E-04	0.0061	25.8	4.3	154.7	Yes	Yes	FALSE
ITGAE	Ultra-rare damaging (MTR)	2	0.0056	3	2.19E-04	0.0061	25.8	4.3	154.7	Yes	.	FALSE
PLEKHA5	Ultra-rare damaging	2	0.0056	3	2.19E-04	0.0061	25.8	4.3	154.7	Yes	.	FALSE

	(MTR)											
PPP3CC	Ultra-rare damaging (MTR)	2	0.0056	3	2.19E-04	0.0061	25.8	4.3	154.7	Yes	.	FALSE
SLC39A13	Ultra-rare damaging (MTR)	2	0.0056	3	2.19E-04	0.0061	25.8	4.3	154.7	Yes	.	FALSE
IGSF1	Flexible non-syn	11	0.0308	169	0.0123	0.0062	2.5	1.4	4.7	.	.	FALSE
TTI1	Flexible non-syn	10	0.028	145	0.0106	0.0062	2.7	1.4	5.2	Yes	.	FALSE
WDR18	Flexible non-syn	6	0.0168	60	0.0044	0.0064	3.9	1.7	9.1	Yes	.	FALSE
PLD2	PTV or rare damaging	6	0.0168	60	0.0044	0.0064	3.9	1.7	9.1	Yes	.	FALSE
GALC	Flexible non-syn	7	0.0196	80	0.0058	0.0065	3.4	1.6	7.4	Yes	.	FALSE
TXNRD1	Flexible non-syn	7	0.0196	80	0.0058	0.0065	3.4	1.6	7.4	Yes	.	FALSE
TRNAU1AP	Flexible non-syn	5	0.014	42	0.0031	0.0065	4.6	1.8	11.8	Yes	.	FALSE
VPS26A	Flexible non-syn	5	0.014	42	0.0031	0.0065	4.6	1.8	11.8	Yes	.	FALSE
MCM10	Flexible damaging	4	0.0112	26	0.0019	0.0066	6	2.1	17.2	.	.	FALSE
CTSB	Flexible non-syn (MTR)	4	0.0112	26	0.0019	0.0066	6	2.1	17.2	Yes	Yes	FALSE
LIMK2	Flexible non-syn (MTR)	4	0.0112	26	0.0019	0.0066	6	2.1	17.2	Yes	.	FALSE

VN1R2	PTV	4	0.0112	26	0.0019	0.0066	6	2.1	17.2	.	.	FALSE
RUSC2	PTV or rare damaging	4	0.0112	26	0.0019	0.0066	6	2.1	17.2	Yes	.	FALSE
PFKL	Rare damaging (MTR)	4	0.0112	26	0.0019	0.0066	6	2.1	17.2	Yes	Yes	FALSE
WDR33	Ultra-rare damaging	4	0.0112	26	0.0019	0.0066	6	2.1	17.2	Yes	.	FALSE
IGSF3	Flexible non-syn	10	0.028	147	0.0107	0.0067	2.7	1.4	5.1	.	.	FALSE
FRMD8	Flexible non-syn	8	0.0224	102	0.0074	0.0069	3.1	1.5	6.3	Yes	.	FALSE
FRY	Flexible non-syn	14	0.0392	241	0.0176	0.007	2.3	1.3	4	Yes	.	FALSE
CFAP53	Flexible non-syn	9	0.0252	125	0.0091	0.0071	2.8	1.4	5.6	.	.	FALSE
ATP5MF-PTCD1	Flexible non-syn (MTR)	5	0.014	43	0.0031	0.0071	4.5	1.8	11.5	Yes	.	TRUE
PTCD1	Flexible non-syn (MTR)	5	0.014	43	0.0031	0.0071	4.5	1.8	11.5	Yes	.	TRUE
ZFAT	Flexible non-syn (MTR)	5	0.014	43	0.0031	0.0071	4.5	1.8	11.5	Yes	.	FALSE
TBC1D1	PTV or rare damaging	5	0.014	43	0.0031	0.0071	4.5	1.8	11.5	Yes	.	FALSE
LSM14B	Flexible damaging	3	0.0084	13	9.47E-04	0.0071	8.9	2.5	31.5	Yes	.	FALSE
ABRAXAS1	Flexible non-syn (MTR)	3	0.0084	13	9.47E-04	0.0071	8.9	2.5	31.5	Yes	.	FALSE
RSPH9	Flexible non-syn (MTR)	3	0.0084	13	9.47E-04	0.0071	8.9	2.5	31.5	.	.	FALSE

TGFB2	Flexible non-syn (MTR)	3	0.0084	13	9.47E-04	0.0071	8.9	2.5	31.5	Yes	.	FALSE
CPEB4	PTV or rare damaging	3	0.0084	13	9.47E-04	0.0071	8.9	2.5	31.5	Yes	.	FALSE
CATSPERE	Rare damaging	3	0.0084	13	9.47E-04	0.0071	8.9	2.5	31.5	.	.	FALSE
CDH7	Rare damaging (MTR)	3	0.0084	13	9.47E-04	0.0071	8.9	2.5	31.5	.	.	FALSE
SMAD2	Rare damaging (MTR)	3	0.0084	13	9.47E-04	0.0071	8.9	2.5	31.5	Yes	.	FALSE
MEP1A	Ultra-rare damaging	3	0.0084	13	9.47E-04	0.0071	8.9	2.5	31.5	.	.	FALSE
TH	Ultra-rare damaging	3	0.0084	13	9.47E-04	0.0071	8.9	2.5	31.5	.	.	FALSE
KIAA0556	Flexible non-syn	17	0.0476	321	0.0234	0.0073	2.1	1.3	3.4	Yes	.	FALSE
KCNA5	Flexible damaging	8	0.0224	103	0.0075	0.0073	3	1.5	6.3	Yes	Yes	FALSE
PHYKPL	Flexible non-syn	8	0.0224	103	0.0075	0.0073	3	1.5	6.3	Yes	.	FALSE
TBX2	Flexible damaging	7	0.0196	82	0.006	0.0074	3.3	1.5	7.3	Yes	Yes	FALSE
SMTNL2	Flexible non-syn	7	0.0196	82	0.006	0.0074	3.3	1.5	7.3	.	.	FALSE
SLC6A8	Flexible non-syn (MTR)	4	0.0112	27	0.002	0.0074	5.7	2	16.5	Yes	.	TRUE
FAM187B	Flexible non-syn	7	0.0196	83	0.006	0.0078	3.3	1.5	7.2	.	.	TRUE
SEMA3F	Flexible damaging	5	0.014	44	0.0032	0.0078	4.4	1.7	11.2	Yes	.	FALSE
ZSCAN2	Flexible non-syn (MTR)	5	0.014	44	0.0032	0.0078	4.4	1.7	11.2	.	.	FALSE

IGSF10	PTV	5	0.014	44	0.0032	0.0078	4.4	1.7	11.2	Yes	.	FALSE
GCDH	PTV or rare damaging	5	0.014	44	0.0032	0.0078	4.4	1.7	11.2	Yes	.	FALSE
KLKB1	Flexible damaging	8	0.0224	105	0.0077	0.008	3	1.4	6.1	.	.	FALSE
F11	Flexible non-syn	8	0.0224	105	0.0077	0.008	3	1.4	6.1	.	.	FALSE
AZGP1	Flexible non-syn	6	0.0168	63	0.0046	0.008	3.7	1.6	8.6	Yes	.	FALSE
ZNF358	Flexible non-syn (MTR)	4	0.0112	28	0.002	0.0083	5.5	1.9	15.9	Yes	Yes	FALSE
CCDC171	Rare damaging (MTR)	4	0.0112	28	0.002	0.0083	5.5	1.9	15.9	.	.	FALSE
SEC31B	Rare damaging (MTR)	4	0.0112	28	0.002	0.0083	5.5	1.9	15.9	Yes	.	FALSE
GCN1	Ultra-rare damaging	4	0.0112	28	0.002	0.0083	5.5	1.9	15.9	Yes	.	FALSE
REEP1	Flexible damaging	3	0.0084	14	0.001	0.0084	8.3	2.4	29	Yes	.	FALSE
DLG2	Flexible non-syn (MTR)	3	0.0084	14	0.001	0.0084	8.3	2.4	29	.	.	FALSE
MGARP	Flexible non-syn (MTR)	3	0.0084	14	0.001	0.0084	8.3	2.4	29	Yes	.	FALSE
LHPP	PTV or rare damaging	3	0.0084	14	0.001	0.0084	8.3	2.4	29	Yes	.	FALSE
NCSTN	PTV or rare damaging	3	0.0084	14	0.001	0.0084	8.3	2.4	29	Yes	.	FALSE
B4GALNT2	Rare damaging	3	0.0084	14	0.001	0.0084	8.3	2.4	29	.	.	FALSE
IVNS1ABP	Ultra-rare damaging	3	0.0084	14	0.001	0.0084	8.3	2.4	29	Yes	.	FALSE

SOX9	Ultra-rare damaging	3	0.0084	14	0.001	0.0084	8.3	2.4	29	Yes	.	FALSE
ABCB11	Ultra-rare damaging (MTR)	3	0.0084	14	0.001	0.0084	8.3	2.4	29	.	.	FALSE
KRT86	Flexible non-syn	8	0.0224	106	0.0077	0.0085	2.9	1.4	6.1	Yes	.	TRUE
CD70	Flexible non-syn	5	0.014	45	0.0033	0.0085	4.3	1.7	10.9	.	.	FALSE
AVIL	Rare damaging	5	0.014	45	0.0033	0.0085	4.3	1.7	10.9	Yes	.	FALSE
CFAP46	Flexible non-syn (MTR)	11	0.0308	177	0.0129	0.0086	2.4	1.3	4.5	.	.	FALSE
NASP	Flexible non-syn	9	0.0252	129	0.0094	0.0086	2.7	1.4	5.4	Yes	.	FALSE
CAMTA1	Flexible damaging	6	0.0168	64	0.0047	0.0086	3.6	1.6	8.5	Yes	Yes	FALSE
TMEM140	Flexible non-syn	6	0.0168	64	0.0047	0.0086	3.6	1.6	8.5	Yes	.	FALSE
SAP130	Flexible non-syn	7	0.0196	85	0.0062	0.0088	3.2	1.5	7	Yes	.	FALSE
PTGES3	Flexible damaging	2	0.0056	4	2.92E-04	0.009	19.3	3.5	105.8	Yes	Yes	FALSE
SMLR1	Flexible damaging	2	0.0056	4	2.92E-04	0.009	19.3	3.5	105.8	.	.	FALSE
C12orf60	Flexible non-syn (MTR)	2	0.0056	4	2.92E-04	0.009	19.3	3.5	105.8	Yes	.	FALSE
NPPA	Flexible non-syn (MTR)	2	0.0056	4	2.92E-04	0.009	19.3	3.5	105.8	Yes	Yes	FALSE
SIGLECL1	Flexible non-syn (MTR)	2	0.0056	4	2.92E-04	0.009	19.3	3.5	105.8	.	.	FALSE

TFEC	Flexible non-syn (MTR)	2	0.0056	4	2.92E-04	0.009	19.3	3.5	105.8	.	.	FALSE
ZNF417	Flexible non-syn (MTR)	2	0.0056	4	2.92E-04	0.009	19.3	3.5	105.8	Yes	.	TRUE
KCNA10	PTV	2	0.0056	4	2.92E-04	0.009	19.3	3.5	105.8	.	.	FALSE
ZNF561	PTV	2	0.0056	4	2.92E-04	0.009	19.3	3.5	105.8	Yes	.	FALSE
ATP6V1D	PTV or rare damaging	2	0.0056	4	2.92E-04	0.009	19.3	3.5	105.8	Yes	.	FALSE
CDC23	PTV or rare damaging	2	0.0056	4	2.92E-04	0.009	19.3	3.5	105.8	Yes	.	FALSE
RAB6A	Rare damaging	2	0.0056	4	2.92E-04	0.009	19.3	3.5	105.8	Yes	Yes	FALSE
CLTB	Rare damaging (MTR)	2	0.0056	4	2.92E-04	0.009	19.3	3.5	105.8	Yes	Yes	FALSE
MTX3	Rare damaging (MTR)	2	0.0056	4	2.92E-04	0.009	19.3	3.5	105.8	Yes	.	FALSE
PI4K2A	Rare damaging (MTR)	2	0.0056	4	2.92E-04	0.009	19.3	3.5	105.8	Yes	.	FALSE
AHCYL1	Ultra-rare damaging	2	0.0056	4	2.92E-04	0.009	19.3	3.5	105.8	Yes	Yes	FALSE
CELA1	Ultra-rare damaging	2	0.0056	4	2.92E-04	0.009	19.3	3.5	105.8	.	.	FALSE
ENDOD1	Ultra-rare damaging	2	0.0056	4	2.92E-04	0.009	19.3	3.5	105.8	Yes	Yes	FALSE
FAM3B	Ultra-rare damaging	2	0.0056	4	2.92E-04	0.009	19.3	3.5	105.8	.	.	TRUE
RILP	Ultra-rare damaging	2	0.0056	4	2.92E-04	0.009	19.3	3.5	105.8	Yes	.	FALSE
RSPH1	Ultra-rare damaging	2	0.0056	4	2.92E-04	0.009	19.3	3.5	105.8	.	.	FALSE

STK31	Ultra-rare damaging	2	0.0056	4	2.92E-04	0.009	19.3	3.5	105.8	.	.	FALSE
ZNF765	Ultra-rare damaging	2	0.0056	4	2.92E-04	0.009	19.3	3.5	105.8	.	.	FALSE
AGAP1	Ultra-rare damaging (MTR)	2	0.0056	4	2.92E-04	0.009	19.3	3.5	105.8	Yes	.	FALSE
AHCY	Ultra-rare damaging (MTR)	2	0.0056	4	2.92E-04	0.009	19.3	3.5	105.8	Yes	.	FALSE
ITCH	Ultra-rare damaging (MTR)	2	0.0056	4	2.92E-04	0.009	19.3	3.5	105.8	Yes	.	FALSE
MAP3K20	Ultra-rare damaging (MTR)	2	0.0056	4	2.92E-04	0.009	19.3	3.5	105.8	Yes	.	FALSE
MDC1	Ultra-rare damaging (MTR)	2	0.0056	4	2.92E-04	0.009	19.3	3.5	105.8	Yes	.	FALSE
PPP2R2D	Ultra-rare damaging (MTR)	2	0.0056	4	2.92E-04	0.009	19.3	3.5	105.8	Yes	.	FALSE
RGL3	Ultra-rare damaging (MTR)	2	0.0056	4	2.92E-04	0.009	19.3	3.5	105.8	Yes	.	FALSE
RGS19	Ultra-rare damaging	2	0.0056	4	2.92E-04	0.009	19.3	3.5	105.8	Yes	.	FALSE

	(MTR)											
RHOBTB2	Ultra-rare damaging (MTR)	2	0.0056	4	2.92E-04	0.009	19.3	3.5	105.8	Yes	.	FALSE
CFAP73	Flexible non-syn	6	0.0168	65	0.0047	0.0091	3.6	1.5	8.3	.	.	FALSE
ERCC4	Rare damaging	6	0.0168	65	0.0047	0.0091	3.6	1.5	8.3	Yes	.	FALSE
RHNO1	Flexible non-syn	5	0.014	46	0.0034	0.0092	4.2	1.7	10.7	Yes	.	FALSE
CCDC38	Flexible non-syn (MTR)	5	0.014	46	0.0034	0.0092	4.2	1.7	10.7	.	.	FALSE
TRIM71	PTV or rare damaging	5	0.014	46	0.0034	0.0092	4.2	1.7	10.7	.	.	TRUE
SLC22A13	Rare damaging	5	0.014	46	0.0034	0.0092	4.2	1.7	10.7	.	.	FALSE
SOGA1	Flexible damaging	4	0.0112	29	0.0021	0.0093	5.4	1.9	15.3	Yes	.	FALSE
THBS1	Flexible non-syn (MTR)	4	0.0112	29	0.0021	0.0093	5.4	1.9	15.3	Yes	Yes	FALSE
ANKRD6	Flexible damaging	8	0.0224	108	0.0079	0.0094	2.9	1.4	6	Yes	.	TRUE
IL12RB2	Flexible non-syn	8	0.0224	108	0.0079	0.0094	2.9	1.4	6	.	.	FALSE
SLC12A7	Flexible non-syn (MTR)	9	0.0252	132	0.0096	0.0098	2.7	1.3	5.3	Yes	.	FALSE
CHRNB2	Flexible non-syn	6	0.0168	66	0.0048	0.0098	3.5	1.5	8.2	.	.	FALSE
HEATR5B	Flexible damaging	7	0.0196	87	0.0063	0.0099	3.1	1.4	6.8	Yes	.	FALSE

OR6K2	Flexible non-syn	7	0.0196	87	0.0063	0.0099	3.1	1.4	6.8	.	.	TRUE
CORT	Flexible non-syn	3	0.0084	15	0.0011	0.0099	7.7	2.2	26.9	.	.	FALSE
CD320	Flexible non-syn (MTR)	3	0.0084	15	0.0011	0.0099	7.7	2.2	26.9	Yes	Yes	FALSE
CDYL2	Flexible non-syn (MTR)	3	0.0084	15	0.0011	0.0099	7.7	2.2	26.9	.	.	FALSE
CENPU	Flexible non-syn (MTR)	3	0.0084	15	0.0011	0.0099	7.7	2.2	26.9	.	.	FALSE
FAM155B	Flexible non-syn (MTR)	3	0.0084	15	0.0011	0.0099	7.7	2.2	26.9	.	.	TRUE
GALNT13	Flexible non-syn (MTR)	3	0.0084	15	0.0011	0.0099	7.7	2.2	26.9	.	.	FALSE
PAQR8	Flexible non-syn (MTR)	3	0.0084	15	0.0011	0.0099	7.7	2.2	26.9	Yes	.	FALSE
STAT6	Flexible non-syn (MTR)	3	0.0084	15	0.0011	0.0099	7.7	2.2	26.9	Yes	Yes	FALSE
TCERG1L	Flexible non-syn (MTR)	3	0.0084	15	0.0011	0.0099	7.7	2.2	26.9	.	.	FALSE
APPL1	PTV	3	0.0084	15	0.0011	0.0099	7.7	2.2	26.9	Yes	.	FALSE
NMI	PTV	3	0.0084	15	0.0011	0.0099	7.7	2.2	26.9	Yes	.	FALSE
SORBS2	Ultra-rare damaging	3	0.0084	15	0.0011	0.0099	7.7	2.2	26.9	Yes	Yes	FALSE

Supplementary Table 6: collapsing analysis results. Capped at $p < 0.01$, does not include synonymous model, for each gene, only the model with the lowest p value is shown. LCI = Lower confidence interval (95%), UCI = Upper confidence interval (95%). All associations are enriched in cases. Gene flagged indicates whether the gene is on a list of genes in which variants are reported at a higher frequency than expected across multiple studies (i.e. they

have a higher than expected mutation rate or are prone to false positive variant calls), or they have another human paralogue that is at least 90% reciprocally identical and therefore also prone to false positive variant calls due to mismapped reads. These associations should be interpreted with caution.

Supplementary Table 7

Gene	Variant (GRCh38)	Function	Transcript	Transcript codon change	Transcript AA change	Sample(s)	Case freq	Ctrl freq	GnomAD exome global AF	GnomAD exome popmax AF
PKD1	16-2090692-G-C	Stop gained	ENST00000262304	c.12120C>G	p.Tyr4040*	ScPt0395467Z	1/357	0/13722	0	0
PKD1	16-2091576-C-A	Missense variant	ENST00000262304	c.11559G>T	p.Glu3853Asp	ScPt0857743D	1/357	0/13722	0	0
PKD1	16-2100038-A-G	Missense variant	ENST00000262304	c.9746T>C	p.Leu3249Pro	ScPt0410594V	1/357	0/13722	0	0
PKD1	16-2107960-G-C	Missense variant	ENST00000262304	c.6988C>G	p.Leu2330Val	ScPt0436380H	1/357	0/13722	0	0
PKD1	16-2112914-G-A	Missense variant	ENST00000262304	c.3035C>T	p.Thr1012Ile	ScPt0795045V	1/357	0/13722	0	0
PKD1	16-2106443-CCA-C	Frameshift variant	ENST00000262304	c.7442_7443delTG	p.Leu2481fs	ScPt0150875X	1/357	0/13722	0	0

COL3A1	2-188990117-C-T	Stop gained	ENST00000304636	c.712C>T	p.Arg238*	ScPt0162409J	1/357	0/13722	0	0
COL3A1	2-189001440-C-T	Missense variant	ENST00000304636	c.2327C>T	p.Pro776Leu	ScPt0792166L	1/357	0/13722	0	0
COL3A1	2-189001446-A-G	Missense variant	ENST00000304636	c.2333A>G	p.Asp778Gly	ScPt0992534F	1/357	0/13722	0	0
SMAD3	15-67170589-G-A	Missense variant	ENST00000327367	c.643G>A	p.Ala215Thr	ScPt0347607Q	1/357	0/13722	0	0
SMAD3	15-67190432-A-AC	Frameshift variant	ENST00000327367	c.1179dupC	p.Cys394fs	ScPt0475518M	1/357	0/13722	0	0
HES5	1-2530113-G-C	Missense variant & splice region variant	ENST00000378453	c.52C>G	p.Arg18Gly	ScPt0580659V BPt00065625	2/357	0/13722	0	0
DLL1	6-170283013-T-C	Missense variant	ENST00000366756	c.2141A>G	p.Lys714Arg	ScPt0409625H	1/357	0/13722	0	0

UMOD	16-20341325-ATGTTTAGAGCAC-A	Frameshift variant	ENST00000302509	c.1332-1_1342delGTGCTCTAAACA	p.Ala445fs	ScPt0649148D	1/357	0/13722	0	0
UMOD	16-20349011-CCTTCGGGGCAGA-CAGGAGGCGG	Protein altering variant	ENST00000302509	c.278_289delinsCCGCCTCCT	p.Val93_Gly97delinsAlaAlaSerCys	ScPt0300070B	1/357	0/13722	0	0

Supplementary Table 7: Details of qualifying variants in cases driving selected associations of interest in collapsing analysis and gene-set enrichment analysis. Qualifying variants in *PKD1* drive signal in collapsing analysis (ultra-rare damaging (MTR) model). Qualifying variants in *PKD1*, *COL3A1*, and *SMAD3* drive signal in SCAD tier 1 genes in gene-set enrichment analysis (ultra-rare damaging (MTR) model). Qualifying variants in *PKD1, HES5*, *DLL1*, and *UMOD* drive signal in Loop of Henle development genes in gene-set enrichment analysis (ultra-rare damaging (MTR) model).

Supplementary Table 8

Gene Name	Gene Function	Expression	Mouse phenotype	Relevant Literature
PKD1	Polycystin-1. Component of the calcium-permeable ion channel	RNA detected in variety of tissues, expression enhanced in the brain. Protein expression enhanced in the urinary system, gastrointestinal tract and reproductive systems.	Range of cardiovascular phenotypes including aortic aneurysm and dissection, abnormal blood vessel morphology and hemorrhage.	Previously implicated in SCAD (39, 46)
PAM	A multifunctional enzyme, which participates in the biosynthesis of neuroendocrine peptides	RNA and protein expression enhanced in heart tissue, detected in variety of tissues	Embryonic lethal with edema. Abnormal artery morphology - thin arterial walls, umbilical vein stenosis and abnormal vitelline vasculature.	Common variant associated with hypertension (166)

GLI3	Transcription factor component of hedgehog signalling pathway.	RNA and protein expression enhanced in the endometrium, but detected in variety of tissues	Abnormal anterior cardinal vein morphology, abnormal pericardium morphology, haemorrhage (166); wide range of musculoskeletal phenotype including polydactyly, abnormal bones morphology and mineralisation, embryonic lethality (167)	Drives angiogenesis during muscle repair (167, 168)
NFATC4	Transcription factor	Non-tissue-specific	Cardiac hypertrophy	Participates in ET-1 induced hypertrophy (169, 170)
SEC24B	Component of COPII vesicles	Non-tissue-specific	Abnormalities in cardiac outflow tract including transposition of great arteries, ventricular hypoplasia; lethality is observed by E17.5.	NA

HDAC9	Histone deacetylase	Non-tissue-specific	Cardiac hypertrophy.	Common variant associated with susceptibility to atherosclerotic disease (171, 172)
COL18A1	Multiplexin. C-terminal fragment is the angiogenesis inhibitor endostatin.	RNA expression enhanced in the liver, protein expression of low tissue specificity	Abnormal retinal blood vessel morphology, abnormal tricuspid valve morphology, intracranial haemorrhage.	Participates in the development of coronary collateral vessels in coronary heart disease (173)
ARNTL	Regulator of circadian rhythm	Non-tissue-specific	Vascular smooth muscle hyperplasia, increased vasoconstriction, decreased heart weight.	Regulates circadian recruitment of leucocytes in veins and arteries (174) Regulates blood pressure via angiotensinogen expression in perivascular adipose tissue (175)

TBX2	Transcription factor involved in cardiovascular development	Non-tissue-specific	Homozygous lethal by E14.5; cardiovascular phenotypes include pericardial oedema, increased heart ventricle size and abnormal morphology of atrioventricular canal.	Rare variants implicated in DiGeorge-like syndrome (176) and conotruncal heart defects (177). Common variants associated with glomerular filtration rate (178) and blood pressure (179).
SOX9	Transcription factor involved in male sexual development	RNA expression enhanced in the salivary gland, protein expression non-tissue-specific	Foetal embryos at E11.5 due to heart failure, thickening of heart valves leaflets and interventricular septum, abnormal organisation of the extracellular matrix at leaflets and pericardial oedema. Heart hypoplasia. Musculoskeletal phenotypes including bone absence and premature bone ossification.	Regulates the expression of transcription factors engaged in heart valve development (180). Reduction of binding to FBLN5 promoter contributes to abdominal aortic aneurysm development (181).
SORBS2	Possible role in muscle contraction and cytoskeletal remodelling	Enhanced RNA expression in heart muscle and urinary bladder	Postnatal lethality in the first week of 40-60% of mice, further development normal, increased heart rate.	Down-regulated in intracranial aneurysm (182); Implicated in congenital heart defects (183).

COL4A2	Component of basement membrane.	RNA expression enhanced in the placenta and heart muscle, protein expression non-tissue specific	Abnormalities in cardiovascular system development: including abnormal morphology of veins and arteries, hemorrhage and heart malformations.	rs4773144, associated with coronary artery disease (184, 185), results in lower expression of COL4A2 leading and susceptibility to plaque rupture (186). Mutations reported in brain small vessel disease (187) and susceptibility to intracerebral hemorrhage (188).

Supplementary Table 8. Annotated selected highly-ranked collapsing analysis results listed in Table 4. Expression pattern was annotated based on the Human Protein Atlas and Genotype-Tissue Expression databases. Mouse phenotypes were collected from MGI.

Supplementary Table 9

Gene-Set Name	Model	Qual Cases	Qual Cases PC	Qual ctrls	Qual Ctrls PC	P value	P value FDR adj	Genes harbouring QVs (n carriers in enriched group) capped at 10 genes	Total n genes in set
SCAD.tier1	Ultra-rare damaging (MTR)	11	3.1%	68	0.5%	3.6E-07	0.033	*PKD1(6); COL3A1(3); SMAD3(2)*	6
GO LOOP OF HENLE DEVELOPMENT	Ultra-rare damaging (MTR)	11	3.1%	76	0.6%	0.0000024	0.11	*PKD1(6); HES5(2); UMOD(2); DLL1(1)*	11
GO HEME BIOSYNTHETIC PROCESS	PTV	9	2.5%	56	0.4%	0.0000034	0.11	*PPOX(2); SLC25A38(2); COX15(1); FECH(1); HMBS(1); IBA57(1); SLC25A39(1)*	20
GO POSITIVE REGULATION OF LAMELLIPODIUM ORGANIZATION	Flexible non-syn (MTR)	43	12%	778	5.7%	0.0000075	0.18	*HDAC4(5); RREB1(5); VIL1(5); WASF2(5); DNM2(4); MTOR(4); SRC(4); FSCN1(3); NCKAP1(3); CORO1B(2)*	24

REACTOME MEMBRANE TRAFFICKING	Rare damaging	106	29.7%	2913	21.2%	0.0000096	0.18	*AP4E1(5); PUM1(5); SEC23A(5); SEC24B(5); DNAJC6(4); DNM2(4); IGF2R(4); AP1M2(3); COPB2(3); GAK(3)*	129
Genefam 703 Protein phosphatases	PTV	3	0.8%	5	0%	0.000015	0.19	*PHPT1(3)*	2
GO CYTOPLASMIC SEQUESTERING OF TRANSCRIPTION FACTOR	Ultra-rare damaging (MTR)	9	2.5%	67	0.5%	0.000017	0.19	*PKD1(6); IL10(1); KEAP1(1); TONSL(1)*	19
GO RAN GTPASE BINDING	Ultra-rare damaging	21	5.9%	286	2.1%	0.000026	0.27	*IPO11(3); IPO7(3); RANBP2(3); XPO4(3); XPO5(2); IPO13(1); IPO4(1); IPO8(1); NUTF2(1); RANBP17(1)*	31
GO TETRAPYRROLE BIOSYNTHETIC PROCESS	Rare damaging (MTR)	20	5.6%	244	1.8%	0.00003	0.28	*FECH(3); SLC25A38(3); ALAS1(2); MMAB(2); PPOX(2); SUCLA2(2); ABCB6(1); COX15(1); HMBS(1); IBA57(1)*	27
GO LYMPH VESSEL MORPHOGENESIS	Ultra-rare damaging (MTR)	9	2.5%	68	0.5%	0.000035	0.28	*PKD1(6); FLT4(2); ACVR2B(1)*	13
GO RESPONSE TO AMINO ACID	Flexible	133	37.3%	3928	28.6%	0.000042	0.28	*COL4A1(10); COL16A1(9); COL5A2(8); CPS1(8);*	112

	damaging							*AARS(7); COL1A1(7); COL6A1(7); EGFR(7); HMGCS2(7); COL1A2(6)*	
GO LIPASE INHIBITOR ACTIVITY	PTV or rare damaging	16	4.5%	198	1.4%	0.000043	0.28	*ANXA5(5); FAF2(3); ANGPTL3(2); APOA1(2); ANXA1(1); ANXA2(1); APOC2(1); APOC3(1)*	18
GO PROTEIN EXPORT FROM NUCLEUS	Ultra-rare damaging (MTR)	15	4.2%	157	1.1%	0.000044	0.28	*PKD1(6); STYX(2); XPO5(2); AHCYL1(1); DUSP16(1); NUP88(1); NUTF2(1); STRADB(1)*	30
GO CHONDROITIN SULFATE BIOSYNTHETIC PROCESS	Flexible non-syn	52	14.6%	3053	22.2%	0.000048	0.28	*VCAN(471); CSPG4(447); CHPF2(285); NCAN(241); XYLT1(193); CHSY1(175); CHPF(152); CSGALNACT1(144); BCAN(141); CHSY3(139)*	25
GO METANEPHRIC EPITHELIUM DEVELOPMENT	Ultra-rare damaging (MTR)	11	3.1%	92	0.7%	0.000054	0.28	*PKD1(6); HES5(2); UMOD(2); WNT9B(1)*	20
GO CELLULAR RESPONSE TO AMINO ACID STIMULUS	Flexible damaging	90	25.2%	2189	16%	0.000066	0.31	*COL4A1(10); COL16A1(9); COL5A2(8); COL1A1(7); COL6A1(7); EGFR(7); HMGCS2(7); COL1A2(6); COL3A1(6); CAPN2(5)*	53

Genefam 1473 Clarins	Ultra-rare damaging	5	1.4%	21	0.2%	0.000069	0.31	*CLRN3(3); CLRN1(1); CLRN2(1)*	3
MODULE 159	Recessive	5	1.4%	37	0.3%	0.000085	0.33	*AARS(1); ARF4(1); GTPBP6(1); KARS(1); NOLC1(1); RRAGA(1)*	81
Genefam 1174 Lysozymes c-type	Flexible damaging	5	1.4%	29	0.2%	0.000087	0.33	*LYZ(2); SPACA3(2); LYZL4(1)*	9
GO N METHYLTRANSFERASE ACTIVITY	Recessive	12	3.4%	131	1%	0.000089	0.33	*SETD2(4); ASH1L(1); DOT1L(1); FDXACB1(1); NSD1(1); PRDM16(1); PRDM2(1); SETD1A(1); WDR77(1)*	88
GO PLACENTA BLOOD VESSEL DEVELOPMENT	Ultra-rare damaging (MTR)	11	3.1%	108	0.8%	0.000094	0.33	*PKD1(6); ESX1(2); PLCD3(2); HEY2(1)*	28
GO REGULATION OF SPINDLE ORGANIZATION	PTV or rare damaging	25	7%	408	3%	0.000094	0.33	*PKD1(13); CHMP2A(2); BORA(1); CHMP1B(1); CHMP2B(1); CHMP4C(1); PDCD6IP(1); PLK1(1); RNF4(1); SENP6(1)*	20
BIOCARTA CALCINEURIN PATHWAY	Rare damaging	13	3.6%	138	1%	0.000098	0.33	*NFATC3(2); NFATC4(2); PLCG1(2); PPP3CC(2); CDKN1A(1); GNAQ(1); NFATC1(1); NFATC2(1);*	21

	(MTR)							*PRKCB(1)*	
REACTOME CHONDROITIN SULFATE BIOSYNTHESIS	Flexible non-syn	42	11.8%	2591	18.9%	0.00013	0.39	*VCAN(471); CSPG4(447); CHPF2(285); NCAN(241); CHSY1(175); CHPF(152); CSGALNACT1(144); BCAN(141); CHSY3(139); CHST15(131)*	21
GO IGG BINDING	Ultra-rare damaging (MTR)	5	1.4%	19	0.1%	0.00015	0.42	*FCGRT(2); UMOD(2); FCER1G(1)*	12
GO NEURON PROJECTION MEMBRANE	PTV	10	2.8%	102	0.7%	0.00015	0.42	*MYO1D(2); ANK1(1); CNTNAP2(1); DDN(1); GRIA1(1); ITGA8(1); NRG1(1); TACR3(1); TRPV1(1)*	36
MODULE 563	Rare damaging	28	7.8%	477	3.5%	0.00018	0.48	*BAIAP3(8); SYNJ1(4); CCL3(3); COPB2(3); SLC1A3(3); CADPS(2); GABRA2(2); ABAT(1); GAD1(1); SNAP25(1)*	15
Genefam 1487 CAP and C-type lectin domain containing	Flexible non-syn (MTR)	2	0.6%	1	0%	0.00019	0.48	*CLEC18A(1); CLEC18B(1)*	3
Genefam 747 Signal regulatory	Recessive	3	0.8%	10	0.1%	0.00021	0.49	*SIRPB1(2); SIRPB2(1)*	6

proteins									
GO CHONDROITIN SULFATE PROTEOGLYCAN BIOSYNTHETIC PROCESS	Flexible non-syn	58	16.2%	3208	23.4%	0.00022	0.49	*VCAN(471); CSPG4(447); CHPF2(285); NCAN(241); XYLT1(193); CHSY1(175); CHPF(152); CSGALNACT1(144); BCAN(141); CHSY3(139)*	30
GO FERROUS IRON BINDING	Ultra-rare damaging	13	3.6%	167	1.2%	0.00023	0.49	*TH(3); FECH(2); HEPH(2); TET2(2); ALKBH3(1); CDO1(1); DNAJC24(1); PLOD1(1)*	22
GO NUCLEAR INCLUSION BODY	Recessive	5	1.4%	31	0.2%	0.00024	0.49	*RANBP2(4); TPR(1)*	12
GO PROTEIN METHYLTRANSFERASE ACTIVITY	Recessive	11	3.1%	122	0.9%	0.00024	0.49	*SETD2(4); ASH1L(1); DOT1L(1); NSD1(1); PRDM16(1); PRDM2(1); SETD1A(1); WDR77(1)*	82
GO REGULATION OF CALCIUM ION TRANSPORT INTO CYTOSOL	Flexible damaging	68	19%	3579	26.1%	0.00025	0.49	*RYR2(508); HTT(252); CAPN3(214); ANK2(209); DMD(195); CACNA1C(159); PTK2B(135); PRKD1(115); NOS1(103); DIAPH1(102)*	92
Genefam 1468 Glutaredoxin domain containing	Flexible non-syn	23	6.4%	411	3%	0.00026	0.49	*GRXCR2(7); TXNRD1(7); GLRX2(4); GRXCR1(2); TXNRD3(2); GLRX3(1); PTGES2(1)*	9
GO CARBOHYDRATE BINDING	Flexible non-	318	89.1%	11257	82%	0.00026	0.49	*PKD1(39); SIGLEC1(16); SI(14); FREM1(13);*	277

	syn							*PKD1L3(13); MGAM(12); ACAN(11); PAM(11); PLA2R1(11); PRG4(11)*	
GO POSITIVE REGULATION OF CYCLIN DEPENDENT PROTEIN KINASE ACTIVITY	PTV or rare damaging	35	9.8%	671	4.9%	0.00027	0.49	*PKD1(13); EGFR(3); SRC(3); STOX1(3); SPDYA(2); ADAM17(1); CCND2(1); CCNH(1); CCNT1(1); CDC6(1)*	36
GO RESPONSE TO OXYGEN LEVELS	Flexible non-syn	317	88.8%	11535	84.1%	0.00027	0.49	*RYR1(21); ATM(16); ITPR1(12); NOS2(12); RYR2(12); PAM(11); COL1A1(10); HIF3A(10); PDGFRB(10); ALDH3A1(9)*	311
GO AXOLEMMA	PTV	5	1.4%	31	0.2%	0.0003	0.51	*MYO1D(2); ANK1(1); CNTNAP2(1); NRG1(1)*	14
MODULE 352	Recessive	5	1.4%	28	0.2%	0.0003	0.51	*RANBP2(4); TPR(1)*	17
GO LYSOZYME ACTIVITY	Flexible damaging	9	2.5%	96	0.7%	0.0003	0.51	*LYG2(3); LYZ(2); SPACA3(2); LYG1(1); LYZL4(1)*	11
Genefam 1453 Condensin II	Flexible non-syn (MTR)	11	3.1%	149	1.1%	0.00031	0.51	*NCAPD3(5); NCAPH2(4); SMC2(2); SMC4(1)*	5
ST GA12 PATHWAY	Flexible non-syn	73	20.4%	1850	13.5%	0.00032	0.51	*PLD2(10); PLD1(9); RASAL1(8); EPHB2(7); PLD3(6); VAV1(5); F2(4); F2RL1(4); F2RL3(4); PTK2(4)*	23

REACTOME SCFSKP2 MEDIATED DEGRADATION OF P27 P21	Recessive	4	1.1%	21	0.2%	0.00033	0.51	*CDKN1B(1); PSMB4(1); PSMF1(1); SKP2(1)*	56
GO RETINAL METABOLIC PROCESS	Flexible non-syn (MTR)	18	5%	256	1.9%	0.00034	0.51	*CYP1B1(9); AKR1C3(3); AKR1C1(2); ALDH1A3(1); ALDH8A1(1); RPE65(1); SDR16C5(1)*	12
CTCTGGA MIR520A MIR525	Flexible non-syn	260	72.8%	8745	63.7%	0.00034	0.51	*BSN(22); THADA(18); NCOR2(17); NUMA1(17); CGN(13); DCHS1(13); SMG1(13); TSHZ1(10); ATRN(9); RAB11FIP1(9)*	158
GO LYMPH VESSEL DEVELOPMENT	Ultra-rare damaging (MTR)	11	3.1%	113	0.8%	0.00036	0.51	*PKD1(6); FLT4(2); ACVR2B(1); FOXC1(1); HEG1(1)*	20
REACTOME INNATE IMMUNE SYSTEM	Flexible damaging	136	38.1%	5946	43.3%	0.00036	0.51	*CREBBP(262); EP300(219); NOD2(158); IFIH1(149); CR1(142); MASP1(136); NOD1(134); NLRX1(133); C3(123); CTSB(121)*	279
Genefam 1221 H/ACA ribonucleoprotein complex	Rare damaging (MTR)	4	1.1%	19	0.1%	0.00038	0.52	*DKC1(1); GAR1(1); NHP2(1); NOP10(1)*	4
GO DNA METHYLATION OR	Ultra-rare	26	7.3%	454	3.3%	0.00038	0.52	*EHMT2(3); MOV10L1(3); TRDMT1(3); ATRX(2);*	59

DEMETHYLATION	damaging							*BAZ2A(2); TET2(2); ALKBH3(1); ASZ1(1); DMAP1(1); DNMT3A(1)*	
GO CILIUM OR FLAGELLUM DEPENDENT CELL MOTILITY	Flexible non-syn	141	39.5%	4424	32.2%	0.00038	0.52	*DNAH6(30); DNAH7(29); DNAH2(28); DNAH3(28); DNAH1(25); DNAH8(23); SPAG16(6); CCDC39(5); DNAAF2(4); RSPH9(4)*	15
GO REGULATION OF CGMP BIOSYNTHETIC PROCESS	PTV	9	2.5%	92	0.7%	0.00039	0.52	*ADORA2B(2); MTNR1A(2); NOS2(2); GUCA1B(1); NOS3(1); PDZD3(1)*	22
Genefam 1311 Alpha arrestins	Rare damaging	10	2.8%	109	0.8%	0.00039	0.52	*ARRDC1(4); ARRDC4(3); ARRDC2(2); ARRDC3(1)*	6
MODULE 84	Flexible damaging	314	88%	10819	78.8%	0.0004	0.52	*PLEC(22); BAIAP3(11); COL6A2(11); COL6A3(11); ITPR3(11); COL4A1(10); ITPR1(10); PPL(10); VWF(10); LAMA3(9)*	549
GO POSITIVE REGULATION OF EXTRACELLULAR MATRIX ORGANIZATION	Ultra-rare damaging	10	2.8%	108	0.8%	0.00042	0.52	*SOX9(3); FSCN1(2); SMAD3(2); TGFB2(2); CPB2(1)*	17
GO NEGATIVE REGULATION OF LYMPHOCYTE	Ultra-rare damaging	17	4.8%	244	1.8%	0.00042	0.52	*GLI3(3); CYLD(2); JAK3(2); PGLYRP3(2); DTX1(1); ERBB2(1); FBXO7(1); HLX(1); IHH(1);*	40

DIFFERENTIATION								*INHBA(1)*	
GO POSITIVE REGULATION OF LAMELLIPODIUM ASSEMBLY	Flexible non-syn (MTR)	28	7.8%	488	3.6%	0.00042	0.52	*HDAC4(5); WASF2(5); DNM2(4); MTOR(4); FSCN1(3); NCKAP1(3); HSP90AA1(2); CLRN1(1); FRMD7(1)*	16
GO POSITIVE REGULATION OF VASOCONSTRICTION	Recessive	6	1.7%	45	0.3%	0.00043	0.52	*AVPR2(1); CD38(1); DBH(1); FGG(1); TBXA2R(1); TRPM4(1)*	35
REACTOME GOLGI ASSOCIATED VESICLE BIOGENESIS	Rare damaging	50	14%	1132	8.2%	0.00044	0.52	*AP4E1(5); PUM1(5); DNAJC6(4); DNM2(4); IGF2R(4); AP1M2(3); GAK(3); TFRC(3); AP1B1(2); ARRB1(2)*	53
REACTOME METABOLISM OF PORPHYRINS	PTV	5	1.4%	29	0.2%	0.00045	0.52	*PPOX(2); BLVRB(1); FECH(1); HMBS(1)*	14
MODULE 169	Ultra-rare damaging (MTR)	28	7.8%	443	3.2%	0.00045	0.52	*MICAL2(3); CCL3(2); RGL3(2); SLC2A5(2); ABCC1(1); CACNA1A(1); CCL4(1); CR2(1); CREG1(1); CYBB(1)*	97
GO REGULATION OF MRNA 3 END PROCESSING	Ultra-rare damaging	14	3.9%	183	1.3%	0.00047	0.53	*CCNB1(3); AHCYL1(2); CNOT1(2); BARD1(1); CCNT1(1); CDC73(1); NCBP2(1); PAF1(1); RNF20(1); SUPT5H(1)*	28

GO RNA SPLICING VIA ENDONUCLEOLYTIC CLEAVAGE AND LIGATION	Flexible non-syn	42	11.8%	850	6.2%	0.00047	0.53	*CPSF1(12); DDX1(4); ERN1(4); ZBTB8OS(4); CLP1(3); TRPT1(3); TSEN2(3); TSEN54(3); CSTF2(2); TSEN34(2)*	16
Genefam 857 Potassium calcium-activated channel subfamily M regulatory beta subunits	Flexible non-syn (MTR)	6	1.7%	48	0.3%	0.00048	0.53	*KCNMB4(3); KCNMB1(1); KCNMB2(1); KCNMB3(1)*	4
Genefam 663 N-terminal EF-hand calcium binding proteins	Flexible damaging	10	2.8%	109	0.8%	0.00048	0.53	*NECAB2(9); NECAB3(1)*	3
GO INTRA S DNA DAMAGE CHECKPOINT	Ultra-rare damaging	9	2.5%	86	0.6%	0.00049	0.53	*MSH2(4); HUS1(2); MDC1(2); XPC(1)*	12
GO REGULATION OF MUSCLE CONTRACTION	Flexible non-syn	212	59.4%	8814	64.2%	0.0005	0.53	*RYR2(568); ANK2(514); DMD(514); SCN10A(482); DSP(431); PLCE1(304); SCN5A(289); DOCK5(276); MYBPC3(272); ACTN3(261)*	147
GO RENAL SYSTEM PROCESS	Rare damaging (MTR)	17	4.8%	1306	9.5%	0.00051	0.53	*KCNMA1(60); WFS1(54); KCNQ1(41); PCSK5(37); ADCY4(36); SLC4A5(36); ADCY5(30); HNF1A(32); ADCY7(31); SULF2(30)*	102
KEGG CYSTEINE AND	Ultra-rare	18	5%	281	2%	0.00051	0.53	*TRDMT1(3); AHCY(2); AHCYL1(2); ADI1(1);*	34

METHIONINE METABOLISM	damaging							*CDO1(1); DNMT3A(1); DNMT3B(1); DNMT3L(1); GOT1(1); GOT2(1)*	
GO LYSINE N METHYLTRANSFERASE ACTIVITY	Rare damaging	53	14.8%	1179	8.6%	0.00051	0.53	*ASH1L(6); EHMT2(5); NSD1(5); SETD1A(5); IRF4(3); PRDM2(3); PRDM6(3); SETD2(3); EZH2(2); MEN1(2)*	57
MODULE 440	Ultra-rare damaging	14	3.9%	183	1.3%	0.00055	0.55	*CPS1(3); GAMT(2); PYCR1(2); ACY1(1); ARG1(1); ASS1(1); CKM(1); CKMT1B(1); DIO1(1); OAT(1)*	19
GO POSITIVE REGULATION OF CHROMOSOME SEGREGATION	Rare damaging	20	5.6%	318	2.3%	0.00055	0.55	*CCNB1(3); RB1(3); RCC2(3); SIRT2(3); SMC6(3); CDC6(1); ESPL1(1); GORASP1(1); PLK1(1); SMC5(1)*	25
REACTOME CYCLIN E ASSOCIATED EVENTS DURING G1 S TRANSITION	Recessive	4	1.1%	23	0.2%	0.00057	0.55	*CDKN1B(1); PSMB4(1); PSMF1(1); SKP2(1)*	65
GO REGULATION OF BONE DEVELOPMENT	PTV	8	2.2%	75	0.5%	0.00057	0.55	*GLI3(2); LTF(2); TJP2(2); POR(1); SLC9B2(1)*	19
GO COATED MEMBRANE	Ultra-rare damaging	43	12%	880	6.4%	0.00058	0.55	*SEC24B(4); AP3D1(3); AP3M2(3); AP4E1(3); AP1B1(2); AP1M2(2); EGFR(2); IGF2R(2);*	87

								SEC23A(2); SEC24C(2)	
WTGAAAT UNKNOWN	Ultra-rare damaging	160	44.8%	6532	47.6%	0.00058	0.55	*ZFHX3(127); SYNE1(106); DST(75); LAMA5(71); CSMD3(68); ITPR3(64); COL7A1(62); NBEAL1(60); ANK2(56); MYH3(53)*	924
GO BLOC 1 COMPLEX	Rare damaging	8	2.2%	79	0.6%	0.00059	0.55	*BCAS4(2); PI4K2A(2); BLOC1S1(1); DTNBP1(1); SNAP25(1); SNAP47(1)*	15
MODULE 539	PTV	7	2%	62	0.5%	0.00061	0.56	*PPOX(2); CP(1); FECH(1); GYPA(1); GYPC(1); HMBS(1)*	16
GO NERVE DEVELOPMENT	Flexible damaging	121	33.9%	3417	24.9%	0.00062	0.56	*UNC13B(8); ERBB3(7); AFG3L2(6); ATP8B1(6); NAV2(6); NRP1(6); SULF1(6); CHRNB2(5); GLI3(5); PLXNA3(5)*	68
GO POSITIVE REGULATION OF MRNA 3 END PROCESSING	Ultra-rare damaging (MTR)	7	2%	55	0.4%	0.00065	0.57	*CNOT1(2); CCNB1(1); CDC73(1); NCBP2(1); PAF1(1); TNRC6C(1)*	17
GO HISTONE LYSINE N METHYLTRANSFERASE ACTIVITY	Recessive	10	2.8%	113	0.8%	0.00065	0.57	*SETD2(4); ASH1L(1); DOT1L(1); NSD1(1); PRDM16(1); PRDM2(1); SETD1A(1)*	45

GO DNA ALKYLATION	Ultra-rare damaging	23	6.4%	399	2.9%	0.00065	0.57	*EHMT2(3); MOV10L1(3); TRDMT1(3); ATRX(2); BAZ2A(2); ASZ1(1); DMAP1(1); DNMT3A(1); DNMT3B(1); DNMT3L(1)*	45
GO TRANSCYTOSIS	Ultra-rare damaging (MTR)	7	2%	55	0.4%	0.00067	0.57	*FCGRT(2); TG(2); USO1(2); SRC(1)*	11
GO RETINOL METABOLIC PROCESS	Flexible non-syn (MTR)	34	9.5%	676	4.9%	0.00067	0.57	*CYP1B1(9); AKR1C3(3); DHRS3(3); PLB1(3); RDH12(3); RETSAT(3); AWAT2(2); DGAT1(2); DGAT2(2); RDH13(2)*	29
GO REGULATION OF PODOSOME ASSEMBLY	PTV or rare damaging	19	5.3%	328	2.4%	0.00069	0.57	*KIF9(3); MAPK9(3); SRC(3); BLK(2); FSCN1(2); HCK(2); LCP1(2); ARHGEF5(1); CAPG(1); GSN(1)*	14
GO CCR CHEMOKINE RECEPTOR BINDING	Rare damaging (MTR)	9	2.5%	92	0.7%	0.00071	0.59	*CCL3(3); CCL17(2); CCL4(1); CCR2(1); CX3CL1(1); JAK1(1)*	35
GO PEPTIDYL LYSINE DIMETHYLATION	Recessive	5	1.4%	31	0.2%	0.00073	0.59	*SETD2(4); ASH1L(1)*	12
GO PURINE NUCLEOTIDE	Flexible non-	38	10.6%	966	7%	0.00073	0.59	*ABCC11(11); SLC35B3(8); CALHM1(5);*	11

TRANSPORT	syn							*SLC25A23(4); SLC25A6(4); SLC25A24(3); SLC35B2(3); GJA1(2); GJB1(2); SLC25A17(2)*	
GO MEMBRANE HYPERPOLARIZATION	Ultra-rare damaging	12	3.4%	151	1.1%	0.00074	0.59	*CFTR(3); KCNQ3(3); KCNA5(2); PRKCZ(2); CACNG2(1); PARK7(1)*	11
REACTOME CELL SURFACE INTERACTIONS AT THE VASCULAR WALL	Rare damaging (MTR)	39	10.9%	771	5.6%	0.00075	0.59	*SLC16A3(3); SRC(3); CD48(2); FN1(2); ITGAM(2); OLR1(2); PLCG1(2); PPIL2(2); SLC7A5(2); COL1A1(1)*	91
Genefam 1377 Haloacid dehalogenase like hydrolase domain containing	PTV or rare damaging	9	2.5%	106	0.8%	0.00078	0.59	*LHPP(3); HDHD2(2); NANP(2); HDHD3(1); HDHD5(1)*	7
PID INTEGRIN5 PATHWAY	Recessive	5	1.4%	32	0.2%	0.00083	0.6	*FBN1(2); FN1(1); ITGA4(1); ITGB7(1)*	17
GO NEGATIVE REGULATION OF IMMUNE RESPONSE	PTV or rare damaging	78	21.8%	2042	14.9%	0.00084	0.6	*COL3A1(6); MASP1(4); CR1(3); FOXF1(3); IL1RL1(3); IRAK3(3); JAK3(3); NMI(3); PGLYRP3(3); SERPINB9(3)*	121
Genefam 662 NBPF members	Recessive	2	0.6%	2	0%	0.00087	0.6	*NBPF20(1); NBPF9(1)*	23
GO REGULATION OF HISTONE METHYLATION	Recessive	16	4.5%	239	1.7%	0.00087	0.6	*MTHFR(7); BCOR(2); TET1(2); BRCA1(1); JARID2(1); KDM4A(1); NSD1(1); RNF20(1)*	56
AAAGGAT MIR501	Recessive	12	3.4%	174	1.3%	0.00089	0.6	*CELSR2(3); WDFY3(2); ALS2(1); ATP6V1H(1);*	126

								CAMTA1(1); KIF1C(1); PHC1(1); SCN3A(1); SYNC(1); ZIC4(1)	
Genefam 89 Zinc fingers RANBP2-type	Recessive	6	1.7%	50	0.4%	0.00091	0.6	*RANBP2(4); ZRANB1(1); ZRANB2(1)*	21
SIRNA EIF4GI UP	PTV	24	6.7%	416	3%	0.00091	0.6	*THADA(3); LAP3(2); MAGEF1(2); NCAPD2(2); PYGL(2); ASAH1(1); ASS1(1); CORO1B(1); ECI1(1); FCAR(1)*	95
GO NEPHRON TUBULE FORMATION	Flexible non-syn	36	10.1%	846	6.2%	0.00091	0.6	*PAX8(5); WNT9B(5); SOX8(4); HNF1B(3); OSR1(3); PAX2(3); SIX1(3); SOX9(3); GREM1(2); HES5(2)*	18
REACTOME TRANS GOLGI NETWORK VESICLE BUDDING	Rare damaging	54	15.1%	1306	9.5%	0.00091	0.6	*AP4E1(5); PUM1(5); DNAJC6(4); DNM2(4); IGF2R(4); AP1M2(3); GAK(3); TFRC(3); AP1B1(2); ARRB1(2)*	60
NFKAPPAB 01	Flexible non-syn (MTR)	169	47.3%	4711	34.3%	0.00095	0.6	*ASH1L(5); NLK(5); UBE4B(5); BMP2K(4); ERN1(4); NFAT5(4); NIPBL(4); PRX(4); STON2(4); UACA(4)*	251
PID RANBP2 PATHWAY	Recessive	4	1.1%	24	0.2%	0.00096	0.6	*RANBP2(4)*	11

GO MRNA SPLICE SITE SELECTION	PTV or rare damaging	27	7.6%	493	3.6%	0.00097	0.6	*SETX(8); NOL3(5); RBMXL1(5); SF3A1(2); SFSWAP(2); ISY1(1); LUC7L2(1); LUC7L3(1); PRPF39(1); SF1(1)*	26
GO NEGATIVE REGULATION OF HEMOPOIESIS	Ultra-rare damaging	41	11.5%	831	6.1%	0.00097	0.6	*GLI3(3); CCL3(2); CYLD(2); HES5(2); JAK3(2); LTF(2); NOTCH1(2); PGLYRP3(2); TJP2(2); CDC73(1)*	128
BIOCARTA AHSP PATHWAY	Recessive	2	0.6%	4	0%	0.00098	0.61	*ALAS2(1); FECH(1)*	13
GO HISTONE METHYLTRANSFERASE ACTIVITY	Recessive	10	2.8%	119	0.9%	0.001	0.63	*SETD2(4); ASH1L(1); DOT1L(1); NSD1(1); PRDM16(1); PRDM2(1); SETD1A(1)*	58
GO RESPONSE TO DRUG	Flexible non-syn	337	94.4%	12729	92.8%	0.001	0.63	*COL18A1(18); CENPF(17); NPC1L1(15); APC(14); MCM7(12); NOS2(12); ATR(11); PAM(11); SCN11A(11); CAD(10)*	431

Supplementary Table 9: Gene-Set Enrichment Analysis (Megagene) Results. Capped at $p < 0.001$, for each gene, only the model with the lowest p value is shown.

Supplementary References

1. Yip A, Saw J. Spontaneous coronary artery dissection-A review. *Cardiovasc Diagn Ther*. 2015;5(1):37-48.

2. Cingolani P, Platts A, Wang LL, et al. A program for annotating and predicting the effects of single nucleotide polymorphisms, SnpEff: SNPs in the genome of Drosophila melanogaster strain w1118; iso-2; iso-3. Fly (Austin). 6:80–92.

3. Zarate S, Carroll A, Krashenina O, et al. Parliament2: Fast Structural Variant Calling Using Optimized Combinations of Callers. bioRxiv 2018:424267.

4. Fan X, Abbott TE, Larson D, Chen K. BreakDancer: Identification of Genomic Structural Variation from Paired-End Read Mapping. Curr. Protoc. Bioinforma. 2014;45:15.6.1-11.

5. Abyzov A, Urban AE, Snyder M, Gerstein M. CNVnator: an approach to discover, genotype, and characterize typical and atypical CNVs from family and population genome sequencing. Genome Res. 2011;21:974–84.

6. Rausch T, Zichner T, Schlattl A, Stütz AM, Benes V, Korbel JO. DELLY: structural variant discovery by integrated paired-end and split-read analysis. Bioinformatics 2012;28:i333–i339.

7. Layer RM, Chiang C, Quinlan AR, Hall IM. LUMPY: a probabilistic framework for structural variant discovery. Genome Biol. 2014;15:R84.

8. Chen X, Schulz-Trieglaff O, Shaw R, et al. Manta: rapid detection of structural variants and indels for germline and cancer sequencing applications. Bioinformatics 2015;32:1220–1222.

9. Geoffroy V, Herenger Y, Kress A, et al. AnnotSV: An integrated tool for Structural Variations annotation. Bioinformatics 2018.

10. Li H, Durbin R. Fast and accurate short read alignment with Burrows-Wheeler transform. Bioinformatics 2009;25:1754–60.

11. DePristo MA, Banks E, Poplin R, et al. A framework for variation discovery and genotyping using next-generation DNA sequencing data. Nat. Genet. 2011;43:491–8.

12. Manichaikul A, Mychaleckyj JC, Rich SS, Daly K, Sale M, Chen W-M. Robust relationship inference in genome-wide association studies. Bioinformatics 2010;26:2867–73.

13. Wang K, Li M, Hakonarson H. ANNOVAR: functional annotation of genetic variants from high-throughput sequencing data. Nucleic Acids Res. 2010;38:e164.

14. Richards S, Aziz N, Bale S, Bick D, Das S, Gastier-Foster J, et al. Standards and guidelines for the interpretation of sequence variants: a joint consensus recommendation of the American College of Medical Genetics and Genomics and the Association for Molecular Pathology. *Genet Med*. 2015;17(5):405-24.

15. Karczewski KJ, Francioli LC, Tiao G, et al. Variation across 141,456 human exomes and genomes reveals the spectrum of loss-of-function intolerance across human protein-coding genes. bioRxiv 2019:531210.

16. Stenson PD, Ball E V, Mort M, et al. Human Gene Mutation Database (HGMD): 2003 update. Hum. Mutat. 2003;21:577–81.

17. Landrum MJ, Lee JM, Benson M, et al. ClinVar: improving access to variant interpretations and supporting evidence. Nucleic Acids Res. 2018;46:D1062–D1067.

18. Richards S, Aziz N, Bale S, et al. Standards and guidelines for the interpretation of sequence variants: a joint consensus recommendation of the American College of Medical Genetics and Genomics and the Association for Molecular Pathology. Genet. Med. 2015;17:405–423.

19. Conrad DF, Pinto D, Redon R, et al. Origins and functional impact of copy number variation in the human genome. Nature 2010;464:704–12.

20. Noll AC, Miller NA, Smith LD, et al. Clinical detection of deletion structural variants in whole-

genome sequences. NPJ genomic Med. 2016;1:16026.

21. Thorvaldsdóttir H, Robinson JT, Mesirov JP. Integrative Genomics Viewer (IGV): High-performance genomics data visualization and exploration. Brief. Bioinform. 2013;14:178–192.

22. Ip E, Chapman G, Winlaw D, Dunwoodie SL, Giannoulatou E. VPOT: A Customizable Variant Prioritization Ordering Tool for Annotated Variants. Genomics. Proteomics Bioinformatics 2019.

23. Jun G, Flickinger M, Hetrick KN, et al. Detecting and estimating contamination of human DNA samples in sequencing and array-based genotype data. Am. J. Hum. Genet. 2012;91:839–48.

24. Purcell S, Neale B, Todd-Brown K, et al. PLINK: A tool set for whole-genome association and population-based linkage analyses. Am. J. Hum. Genet. 2007;81:559–575.

25. Pedersen BS, Quinlan AR. Who's Who? Detecting and Resolving Sample Anomalies in Human DNA Sequencing Studies with Peddy. Am. J. Hum. Genet. 2017;100:406–413.

26. Ioannidis NM, Rothstein JH, Pejaver V, et al. REVEL: An Ensemble Method for Predicting the Pathogenicity of Rare Missense Variants. Am. J. Hum. Genet. 2016;99:877–885.

27. Rebhan M, Chalifa-Caspi V, Prilusky J, Lancet D. GeneCards: integrating information about genes, proteins and diseases. Trends Genet. 1997;13:163.

28. Amberger JS, Bocchini CA, Schiettecatte F, Scott AF, Hamosh A. OMIM.org: Supplementary Mendelian Inheritance in Man (OMIM®), an Supplementary catalog of human genes and genetic disorders. Nucleic Acids Res. 2015;43:D789-98.

29. Uhlén M, Fagerberg L, Hallström BM, et al. Proteomics. Tissue-based map of the human proteome. Science 2015;347:1260419.

30. Bult CJ, Blake JA, Smith CL, Kadin JA, Richardson JE, Mouse Genome Database Group. Mouse Genome Database (MGD) 2019. Nucleic Acids Res. 2019;47:D801–D806.

31. Vitsios D, Petrovski S. Stochastic semi-supervised learning to prioritise genes from high-throughput genomic screens. bioRxiv 2019:655449.

32. Epi4K consortium, Epilepsy Phenome/Genome Project. Ultra-rare genetic variation in common epilepsies: a case-control sequencing study. Lancet Neurol. 2017;16(2):135-143.

33. Subramanian A, Tamayo P, Mootha VK, et al. Gene set enrichment analysis: a knowledge-based approach for interpreting genome-wide expression profiles. Proc. Natl. Acad. Sci. U. S. A. 2005;102:15545–50.

34. Yates B, Braschi B, Gray KA, Seal RL, Tweedie S, Bruford EA. Genenames.org: the HGNC and VGNC resources in 2017. Nucleic Acids Res. 2017;45:D619–D625.

35. Cameron-Christie S, Wolock CJ, Groopman E, Petrovski S, Kamalakaran S, Povysil G, et al. Exome-Based Rare-Variant Analyses in CKD. J Am Soc Nephrol. 2019;30(6):1109-1122.

36. Kaadan MI, MacDonald C, Ponzini F, et al. Prospective Cardiovascular Genetics Evaluation in Spontaneous Coronary Artery Dissection. Circ. Genomic Precis. Med. 2018;11:e001933.

37. Nakamura M, Yajima J, Oikawa Y, et al. Vascular Ehlers-Danlos syndrome--all three coronary artery spontaneous dissections. J. Cardiol. 2009;53:458–62.

38. Hampole C V, Philip F, Shafii A, et al. Spontaneous coronary artery dissection in Ehlers-Danlos syndrome. Ann. Thorac. Surg. 2011;92:1883–4.

39. Henkin S, Negrotto SM, Tweet MS, et al. Spontaneous coronary artery dissection and its association with heritable connective tissue disorders. Heart 2016;102:876–81.

40. Schwarze U, Goldstein JA, Byers PH. Splicing defects in the COL3A1 gene: marked preference for 5' (donor) spice-site mutations in patients with exon-skipping mutations and Ehlers-Danlos syndrome type IV. Am. J. Hum. Genet. 1997;61:1276–86.

41. von Hundelshausen P, Oexle K, Bidzhekov K, et al. Recurrent spontaneous coronary dissections

in a patient with a de novo fibrillin-1 mutation without Marfan syndrome. Thromb. Haemost. 2015;113:668–70.

42. Dietz HC, Cutting GR, Pyeritz RE, et al. Marfan syndrome caused by a recurrent de novo missense mutation in the fibrillin gene. Nature 1991;352:337–9.

43. Itty CT, Farshid A, Talaulikar G. Spontaneous coronary artery dissection in a woman with polycystic kidney disease. Am. J. Kidney Dis. 2009;53:518–21.

44. Anon. The polycystic kidney disease 1 gene encodes a 14 kb transcript and lies within a duplicated region on chromosome 16. The European Polycystic Kidney Disease Consortium. Cell 1994;77:881–94.

45. Garcia-Bermúdez M, Moustafa A-H, Barrós-Membrilla A, Tizón-Marcos H. Repeated Loss of Consciousness in a Young Woman: A Suspicious SMAD3 Mutation Underlying Spontaneous Coronary Artery Dissection. Can. J. Cardiol. 2017;33:292.e1-292.e3.

46. van de Laar IMBH, Oldenburg RA, Pals G, et al. Mutations in SMAD3 cause a syndromic form of aortic aneurysms and dissections with early-onset osteoarthritis. Nat. Genet. 2011;43:121–6.

47. Turley TN, Theis JL, Sundsbak RS, et al. Rare Missense Variants in TLN1 Are Associated With Familial and Sporadic Spontaneous Coronary Artery Dissection. Circ. Genomic Precis. Med. 2019;12:e002437.

48. Sun Y, Chen Y, Li Y, et al. Association of TSR1 Variants and Spontaneous Coronary Artery Dissection. J. Am. Coll. Cardiol. 2019;74:167–176.

49. Bergen AA, Plomp AS, Schuurman EJ, et al. Mutations in ABCC6 cause pseudoxanthoma elasticum. Nat. Genet. 2000;25:228–31.

50. Wang X, Charng W-L, Chen C-A, et al. Germline mutations in ABL1 cause an autosomal dominant syndrome characterized by congenital heart defects and skeletal malformations. Nat. Genet.

2017;49:613–617.

51. Guo D-C, Pannu H, Tran-Fadulu V, et al. Mutations in smooth muscle alpha-actin (ACTA2) lead to thoracic aortic aneurysms and dissections. Nat. Genet. 2007;39:1488–93.

52. Milewicz DM, Østergaard JR, Ala-Kokko LM, et al. De novo ACTA2 mutation causes a novel syndrome of multisystemic smooth muscle dysfunction. Am. J. Med. Genet. A 2010;152A:2437–43.

53. Kaplan JM, Kim SH, North KN, et al. Mutations in ACTN4, encoding alpha-actinin-4, cause familial focal segmental glomerulosclerosis. Nat. Genet. 2000;24:251–6.

54. Veluchamy A, Ballerini L, Vitart V, et al. Novel locus influencing retinal venular tortuosity is also associated with risk of coronary artery disease. bioRxiv 2018:121012.

55. Johnson DW, Berg JN, Baldwin MA, et al. Mutations in the activin receptor-like kinase 1 gene in hereditary haemorrhagic telangiectasia type 2. Nat. Genet. 1996;13:189–95.

56. Colige A, Sieron AL, Li SW, et al. Human Ehlers-Danlos syndrome type VII C and bovine dermatosparaxis are caused by mutations in the procollagen I N-proteinase gene. Am. J. Hum. Genet. 1999;65:308–17.

57. Alazami AM, Al-Qattan SM, Faqeih E, et al. Expanding the clinical and genetic heterogeneity of hereditary disorders of connective tissue. Hum. Genet. 2016;135:525–540.

58. Baumgartner MR, Hu CA, Almashanu S, et al. Hyperammonemia with reduced ornithine, citrulline, arginine and proline: a new inborn error caused by a mutation in the gene encoding delta(1)-pyrroline-5-carboxylate synthase. Hum. Mol. Genet. 2000;9:2853–8.

59. Levy E, Carman MD, Fernandez-Madrid IJ, et al. Mutation of the Alzheimer’s disease amyloid gene in hereditary cerebral hemorrhage, Dutch type. Science 1990;248:1124–6.

60. Southgate L, Machado RD, Snape KM, et al. Gain-of-function mutations of ARHGAP31, a Cdc42/Rac1 GTPase regulator, cause syndromic cutis aplasia and limb anomalies. Am. J. Hum.

Genet. 2011;88:574–85.

61. Conley YP, Jakobsdottir J, Mah T, et al. CFH, ELOVL4, PLEKHA1 and LOC387715 genes and susceptibility to age-related maculopathy: AREDS and CHS cohorts and meta-analyses. Hum. Mol. Genet. 2006;15:3206–18.

62. Gormley P, Anttila V, Winsvold BS, et al. Meta-analysis of 375,000 individuals identifies 38 susceptibility loci for migraine. Nat. Genet. 2016;48:856–66.

63. Kornak U, Reynders E, Dimopoulou A, et al. Impaired glycosylation and cutis laxa caused by mutations in the vesicular H+-ATPase subunit ATP6V0A2. Nat. Genet. 2008;40:32–4.

64. Van Damme T, Gardeitchik T, Mohamed M, et al. Mutations in ATP6V1E1 or ATP6V1A Cause Autosomal-Recessive Cutis Laxa. Am. J. Hum. Genet. 2017;100:216–227.

65. Nakajima M, Mizumoto S, Miyake N, et al. Mutations in B3GALT6, which encodes a glycosaminoglycan linker region enzyme, cause a spectrum of skeletal and connective tissue disorders. Am. J. Hum. Genet. 2013;92:927–34.

66. Okajima T, Fukumoto S, Furukawa K, Urano T. Molecular basis for the progeroid variant of Ehlers-Danlos syndrome. Identification and characterization of two mutations in galactosyltransferase I gene. J. Biol. Chem. 1999;274:28841–4.

67. Meester JAN, Vandeweyer G, Pintelon I, et al. Loss-of-function mutations in the X-linked biglycan gene cause a severe syndromic form of thoracic aortic aneurysms and dissections. Genet. Med. 2017;19:386–395.

68. Kapferer-Seebacher I, Pepin M, Werner R, et al. Periodontal Ehlers-Danlos Syndrome Is Caused by Mutations in C1R and C1S, which Encode Subcomponents C1r and C1s of Complement. Am. J. Hum. Genet. 2016;99:1005–1014.

69. Olin JW, Di Narzo AF, D'Escamard V, et al. A Plasma Proteogenomic Signature for

Fibromuscular Dysplasia. Cardiovasc. Res. 2019.

70. Dündar M, Müller T, Zhang Q, et al. Loss of dermatan-4-sulfotransferase 1 function results in adducted thumb-clubfoot syndrome. Am. J. Hum. Genet. 2009;85:873–82.

71. Zou Y, Zwolanek D, Izu Y, et al. Recessive and dominant mutations in COL12A1 cause a novel EDS/myopathy overlap syndrome in humans and mice. Hum. Mol. Genet. 2014;23:2339–52.

72. Zekavat SM, Kii E, Chaffin M, et al. Abstract 15404: Whole Exome Sequencing of Spontaneous Coronary Artery Dissection (SCAD) Cases Discovers Enrichment of Rare Disruptive Variants in Fibrillar Collagen Genes. Circulation 2018;138:A15404–A15404.

73. Malfait F, Francomano C, Byers P, et al. The 2017 international classification of the Ehlers-Danlos syndromes. Am. J. Med. Genet. C. Semin. Med. Genet. 2017;175:8–26.

74. Mayer SA, Rubin BS, Starman BJ, Byers PH. Spontaneous multivessel cervical artery dissection in a patient with a substitution of alanine for glycine (G13A) in the alpha 1 (I) chain of type I collagen. Neurology 1996;47:552–6.

75. Malfait F, Hakim AJ, De Paepe A, Grahame R. The genetic basis of the joint hypermobility syndromes. Rheumatology (Oxford). 2006;45:502–7.

76. Gonzaga-Jauregui C, Gamble CN, Yuan B, et al. Mutations in COL27A1 cause Steel syndrome and suggest a founder mutation effect in the Puerto Rican population. Eur. J. Hum. Genet. 2015;23:342–6.

77. Deng H, Huang X, Yuan L. Molecular genetics of the COL2A1-related disorders. Mutat. Res. Rev. Mutat. Res. 768:1–13.

78. Wenstrup RJ, Florer JB, Willing MC, et al. COL5A1 haploinsufficiency is a common molecular mechanism underlying the classical form of EDS. Am. J. Hum. Genet. 2000;66:1766–76.

79. Symoens S, Syx D, Malfait F, et al. Comprehensive molecular analysis demonstrates type V

collagen mutations in over 90% of patients with classic EDS and allows to refine diagnostic criteria. Hum. Mutat. 2012;33:1485–93.

80. Jöbsis GJ, Keizers H, Vreijling JP, et al. Type VI collagen mutations in Bethlem myopathy, an autosomal dominant myopathy with contractures. Nat. Genet. 1996;14:113–5.

81. Demir E, Sabatelli P, Allamand V, et al. Mutations in COL6A3 cause severe and mild phenotypes of Ullrich congenital muscular dystrophy. Am. J. Hum. Genet. 2002;70:1446–58.

82. Hecht JT, Nelson LD, Crowder E, et al. Mutations in exon 17B of cartilage oligomeric matrix protein (COMP) cause pseudoachondroplasia. Nat. Genet. 1995;10:325–9.

83. Dinauer MC, Pierce EA, Bruns GA, Curnutte JT, Orkin SH. Human neutrophil cytochrome b light chain (p22-phox). Gene structure, chromosomal location, and mutations in cytochrome-negative autosomal recessive chronic granulomatous disease. J. Clin. Invest. 1990;86:1729–37.

84. Murase Y, Yamada Y, Hirashiki A, et al. Genetic risk and gene-environment interaction in coronary artery spasm in Japanese men and women. Eur. Heart J. 2004;25:970–7.

85. Cappello S, Gray MJ, Badouel C, et al. Mutations in genes encoding the cadherin receptor-ligand pair DCHS1 and FAT4 disrupt cerebral cortical development. Nat. Genet. 2013;45:1300–8.

86. Müller T, Mizumoto S, Suresh I, et al. Loss of dermatan sulfate epimerase (DSE) function results in musculocontractural Ehlers-Danlos syndrome. Hum. Mol. Genet. 2013;22:3761–72.

87. Adlam D, Olson TM, Combaret N, et al. Association of the PHACTR1/EDN1 Genetic Locus With Spontaneous Coronary Artery Dissection. J. Am. Coll. Cardiol. 2019;73:58–66.

88. Gordon CT, Petit F, Kroisel PM, et al. Mutations in endothelin 1 cause recessive auriculocondylar syndrome and dominant isolated question-mark ears. Am. J. Hum. Genet. 2013;93:1118–25.

89. Hucthagowder V, Sausgruber N, Kim KH, Angle B, Marmorstein LY, Urban Z. Fibulin-4: a novel gene for an autosomal recessive cutis laxa syndrome. Am. J. Hum. Genet. 2006;78:1075–80.

90. Ciurică S, Lopez-Sublet M, Loeys BL, et al. Arterial Tortuosity. Hypertens. (Dallas, Tex. 1979) 2019;73:951–960.

91. Ewart AK, Jin W, Atkinson D, Morris CA, Keating MT. Supravalvular aortic stenosis associated with a deletion disrupting the elastin gene. J. Clin. Invest. 1994;93:1071–7.

92. Capuano A, Bucciotti F, Farwell KD, et al. Diagnostic Exome Sequencing Identifies a Novel Gene, EMILIN1, Associated with Autosomal-Dominant Hereditary Connective Tissue Disease. Hum. Mutat. 2016;37:84–97.

93. Jones GT, Tromp G, Kuivaniemi H, et al. Meta-Analysis of Genome-Wide Association Studies for Abdominal Aortic Aneurysm Identifies Four New Disease-Specific Risk Loci. Circ. Res. 2017;120:341–353.

94. Khan T, Danyi P, Topaz O, Ali A, Jovin IS. Spontaneous coronary artery dissection in a young man with a factor v leiden gene mutation: a case report and review of the literature. Int. J. Angiol. 2013;22:251–4.

95. Guasch JF, Cannegieter S, Reitsma PH, van't Veer-Korthof ET, Bertina RM. Severe coagulation factor V deficiency caused by a 4 bp deletion in the factor V gene. Br. J. Haematol. 1998;101:32–9.

96. Loeys B, Van Maldergem L, Mortier G, et al. Homozygosity for a missense mutation in fibulin-5 (FBLN5) results in a severe form of cutis laxa. Hum. Mol. Genet. 2002;11:2113–8.

97. Putnam EA, Zhang H, Ramirez F, Milewicz DM. Fibrillin-2 (FBN2) mutations result in the Marfan-like disorder, congenital contractural arachnodactyly. Nat. Genet. 1995;11:456–8.

98. Baumann M, Giunta C, Krabichler B, et al. Mutations in FKBP14 cause a variant of Ehlers-Danlos syndrome with progressive kyphoscoliosis, myopathy, and hearing loss. Am. J. Hum. Genet. 2012;90:201–16.

99. Nickerson ML, Warren MB, Toro JR, et al. Mutations in a novel gene lead to kidney tumors, lung

wall defects, and benign tumors of the hair follicle in patients with the Birt-Hogg-Dubé syndrome. Cancer Cell 2002;2:157–64.

100. Kyndt F, Gueffet J-P, Probst V, et al. Mutations in the gene encoding filamin A as a cause for familial cardiac valvular dystrophy. Circulation 2007;115:40–9.

101. Krakow D, Robertson SP, King LM, et al. Mutations in the gene encoding filamin B disrupt vertebral segmentation, joint formation and skeletogenesis. Nat. Genet. 2004;36:405–10.

102. Kuang S-Q, Medina-Martinez O, Guo D-C, et al. FOXE3 mutations predispose to thoracic aortic aneurysms and dissections. J. Clin. Invest. 2016;126:948–61.

103. Paznekas WA, Boyadjiev SA, Shapiro RE, et al. Connexin 43 (GJA1) mutations cause the pleiotropic phenotype of oculodentodigital dysplasia. Am. J. Hum. Genet. 2003;72:408–18.

104. Yu Q, Shen Y, Chatterjee B, et al. ENU induced mutations causing congenital cardiovascular anomalies. Development 2004;131:6211–23.

105. Hennies HC, Kornak U, Zhang H, et al. Gerodermia osteodysplastica is caused by mutations in SCYL1BP1, a Rab-6 interacting golgin. Nat. Genet. 2008;40:1410–2.

106. Endele S, Rosenberger G, Geider K, et al. Mutations in GRIN2A and GRIN2B encoding regulatory subunits of NMDA receptors cause variable neurodevelopmental phenotypes. Nat. Genet. 2010;42:1021–6.

107. Carmona FD, Coit P, Saruhan-Direskeneli G, et al. Analysis of the common genetic component of large-vessel vasculitides through a meta-Immunochip strategy. Sci. Rep. 2017;7:43953.

108. Pang J, Zhang S, Yang P, et al. Loss-of-function mutations in HPSE2 cause the autosomal recessive urofacial syndrome. Am. J. Hum. Genet. 2010;86:957–62.

109. Debette S, Markus HS. The genetics of cervical artery dissection: a systematic review. Stroke 2009;40:e459-66.

110. Altare F, Lammas D, Revy P, et al. Inherited interleukin 12 deficiency in a child with bacille Calmette-Guérin and Salmonella enteritidis disseminated infection. J. Clin. Invest. 1998;102:2035–40.

111. Renauer PA, Saruhan-Direskeneli G, Coit P, et al. Identification of Susceptibility Loci in IL6, RPS9/LILRB3, and an Intergenic Locus on Chromosome 21q22 in Takayasu Arteritis in a Genome-Wide Association Study. Arthritis Rheumatol. (Hoboken, N.J.) 2015;67:1361–8.

112. Ghanemi A, St-Amand J. Interleukin-6 as a “metabolic hormone”. Cytokine 2018;112:132–136.

113. Oda T, Elkahloun AG, Pike BL, et al. Mutations in the human Jagged1 gene are responsible for Alagille syndrome. Nat. Genet. 1997;16:235–42.

114. Plaster NM, Tawil R, Tristani-Firouzi M, et al. Mutations in Kir2.1 cause the developmental and episodic electrical phenotypes of Andersen’s syndrome. Cell 2001;105:511–9.

115. Dreyer SD, Zhou G, Baldini A, et al. Mutations in LMX1B cause abnormal skeletal patterning and renal dysplasia in nail patella syndrome. Nat. Genet. 1998;19:47–50.

116. Guo D, Regalado ES, Gong L, et al. LOX Mutations Predispose to Thoracic Aortic Aneurysms and Dissections. Circ. Res. 2016;118:928–34.

117. Guo D-C, Regalado ES, Pinard A, et al. LTBP3 Pathogenic Variants Predispose Individuals to Thoracic Aortic Aneurysms and Dissections. Am. J. Hum. Genet. 2018;102:706–712.

118. Urban Z, Hucthagowder V, Schürmann N, et al. Mutations in LTBP4 cause a syndrome of impaired pulmonary, gastrointestinal, genitourinary, musculoskeletal, and dermal development. Am. J. Hum. Genet. 2009;85:593–605.

119. Guo D, Gong L, Regalado ES, et al. MAT2A mutations predispose individuals to thoracic aortic aneurysms. Am. J. Hum. Genet. 2015;96:170–7.

120. Schwartz CE, Tarpey PS, Lubs HA, et al. The original Lujan syndrome family has a novel

missense mutation (p.N1007S) in the MED12 gene. J. Med. Genet. 2007;44:472–7.

121. Barbier M, Gross M-S, Aubart M, et al. MFAP5 loss-of-function mutations underscore the involvement of matrix alteration in the pathogenesis of familial thoracic aortic aneurysms and dissections. Am. J. Hum. Genet. 2014;95:736–43.

122. Ye S, Eriksson P, Hamsten A, Kurkinen M, Humphries SE, Henney AM. Progression of coronary atherosclerosis is associated with a common genetic variant of the human stromelysin-1 promoter which results in reduced gene expression. J. Biol. Chem. 1996;271:13055–60.

123. McColgan P, Sharma P. The genetics of carotid dissection: meta-analysis of a MTHFR/C677T common molecular variant. Cerebrovasc. Dis. 2008;25:561–5.

124. Zhu L, Vranckx R, Khau Van Kien P, et al. Mutations in myosin heavy chain 11 cause a syndrome associating thoracic aortic aneurysm/aortic dissection and patent ductus arteriosus. Nat. Genet. 2006;38:343–9.

125. Wang L, Guo D, Cao J, et al. Mutations in myosin light chain kinase cause familial aortic dissections. Am. J. Hum. Genet. 2010;87:701–7.

126. Chang K, Baek SH, Seung K-B, et al. The Glu298Asp polymorphism in the endothelial nitric oxide synthase gene is strongly associated with coronary spasm. Coron. Artery Dis. 2003;14:293–9.

127. Garg V, Muth AN, Ransom JF, et al. Mutations in NOTCH1 cause aortic valve disease. Nature 2005;437:270–4.

128. Carmona FD, Vaglio A, Mackie SL, et al. A Genome-wide Association Study Identifies Risk Alleles in Plasminogen and P4HA2 Associated with Giant Cell Arteritis. Am. J. Hum. Genet. 2017;100:64–74.

129. Wooten EC, Iyer LK, Montefusco MC, et al. Application of gene network analysis techniques identifies AXIN1/PDIA2 and endoglin haplotypes associated with bicuspid aortic valve. PLoS One

2010;5:e8830.

130. Hinkes B, Wiggins RC, Gbadegesin R, et al. Positional cloning uncovers mutations in PLCE1 responsible for a nephrotic syndrome variant that may be reversible. Nat. Genet. 2006;38:1397–405.

131. Tefs K, Gueorguieva M, Klammt J, et al. Molecular and clinical spectrum of type I plasminogen deficiency: A series of 50 patients. Blood 2006;108:3021–6.

132. Yeowell HN, Walker LC. Mutations in the lysyl hydroxylase 1 gene that result in enzyme deficiency and the clinical phenotype of Ehlers-Danlos syndrome type VI. Mol. Genet. Metab. 71:212–24.

133. Arndt A-K, Schafer S, Drenckhahn J-D, et al. Fine mapping of the 1p36 deletion syndrome identifies mutation of PRDM16 as a cause of cardiomyopathy. Am. J. Hum. Genet. 2013;93:67–77.

134. Burkitt Wright EMM, Spencer HL, Daly SB, et al. Mutations in PRDM5 in brittle cornea syndrome identify a pathway regulating extracellular matrix development and maintenance. Am. J. Hum. Genet. 2011;88:767–777.

135. Guo D, Regalado E, Casteel DE, et al. Recurrent gain-of-function mutation in PRKG1 causes thoracic aortic aneurysms and acute aortic dissections. Am. J. Hum. Genet. 2013;93:398–404.

136. Georges A, Albuisson J, Dupré D, et al. PgmNr 2728/T: Mutations in the prostaglandin I2 receptor gene as potential causes of fibromuscular dysplasia. In: American Society of Human Genetics Annual Meeting., 2019.

137. Guernsey DL, Jiang H, Evans SC, et al. Mutation in pyrroline-5-carboxylate reductase 1 gene in families with cutis laxa type 2. Am. J. Hum. Genet. 2009;85:120–9.

138. Reversade B, Escande-Beillard N, Dimopoulou A, et al. Mutations in PYCR1 cause cutis laxa with progeroid features. Nat. Genet. 2009;41:1016–21.

139. Basel-Vanagaite L, Sarig O, Hershkovitz D, et al. RIN2 deficiency results in macrocephaly,

alopecia, cutis laxa, and scoliosis: MACS syndrome. Am. J. Hum. Genet. 2009;85:254–63.

140. Jen JC, Chan W-M, Bosley TM, et al. Mutations in a human ROBO gene disrupt hindbrain axon pathway crossing and morphogenesis. Science 2004;304:1509–13.

141. Gould RA, Aziz H, Woods CE, et al. ROBO4 variants predispose individuals to bicuspid aortic valve and thoracic aortic aneurysm. Nat. Genet. 2019;51:42–50.

142. Shigehara Y, Okuda S, Nemer G, et al. Mutations in SDR9C7 gene encoding an enzyme for vitamin A metabolism underlie autosomal recessive congenital ichthyosis. Hum. Mol. Genet. 2016;25:4484–4493.

143. Doyle AJ, Doyle JJ, Bessling SL, et al. Mutations in the TGF-β repressor SKI cause Shprintzen-Goldberg syndrome with aortic aneurysm. Nat. Genet. 2012;44:1249–54.

144. Coucke PJ, Willaert A, Wessels MW, et al. Mutations in the facilitative glucose transporter GLUT10 alter angiogenesis and cause arterial tortuosity syndrome. Nat. Genet. 2006;38:452–7.

145. Giunta C, Elçioglu NH, Albrecht B, et al. Spondylocheiro dysplastic form of the Ehlers-Danlos syndrome--an autosomal-recessive entity caused by mutations in the zinc transporter gene SLC39A13. Am. J. Hum. Genet. 2008;82:1290–305.

146. Le Goff C, Mahaut C, Abhyankar A, et al. Mutations at a single codon in Mad homology 2 domain of SMAD4 cause Myhre syndrome. Nat. Genet. 2011;44:85–8.

147. Tan HL, Glen E, Töpf A, et al. Nonsynonymous variants in the SMAD6 gene predispose to congenital cardiovascular malformation. Hum. Mutat. 2012;33:720–7.

148. Sherman EA, Strauss KA, Tortorelli S, et al. Genetic mapping of glutaric aciduria, type 3, to chromosome 7 and identification of mutations in c7orf10. Am. J. Hum. Genet. 2008;83:604–9.

149. Kirk EP, Sunde M, Costa MW, et al. Mutations in cardiac T-box factor gene TBX20 are associated with diverse cardiac pathologies, including defects of septation and valvulogenesis and

cardiomyopathy. Am. J. Hum. Genet. 2007;81:280–91.

150. Lindsay ME, Schepers D, Bolar NA, et al. Loss-of-function mutations in TGFB2 cause a syndromic presentation of thoracic aortic aneurysm. Nat. Genet. 2012;44:922–7.

151 Rienhoff HY, Yeo C-Y, Morissette R, et al. A mutation in TGFB3 associated with a syndrome of low muscle mass, growth retardation, distal arthrogryposis and clinical features overlapping with Marfan and Loeys-Dietz syndrome. Am. J. Med. Genet. A 2013;161A:2040–6.

152. Loeys BL, Chen J, Neptune ER, et al. A syndrome of altered cardiovascular, craniofacial, neurocognitive and skeletal development caused by mutations in TGFBR1 or TGFBR2. Nat. Genet. 2005;37:275–81.

153. Fattori R, Sangiorgio P, Mariucci E, et al. Spontaneous coronary artery dissection in a young woman with Loeys-Dietz syndrome. Am. J. Med. Genet. A 2012;158A:1216–8.

154. Mizuguchi T, Collod-Beroud G, Akiyama T, et al. Heterozygous TGFBR2 mutations in Marfan syndrome. Nat. Genet. 2004;36:855–60.

155. Burch GH, Gong Y, Liu W, et al. Tenascin-X deficiency is associated with Ehlers-Danlos syndrome. Nat. Genet. 1997;17:104–8.

156. Williamson KA, Rainger J, Floyd JAB, et al. Heterozygous loss-of-function mutations in YAP1 cause both isolated and syndromic optic fissure closure defects. Am. J. Hum. Genet. 2014;94:295–302.

157. Guo D-C, Duan X-Y, Regalado ES, et al. Loss-of-Function Mutations in YY1AP1 Lead to Grange Syndrome and a Fibromuscular Dysplasia-Like Vascular Disease. Am. J. Hum. Genet. 2017;100:21–30.

158. Abu A, Frydman M, Marek D, et al. Deleterious mutations in the Zinc-Finger 469 gene cause brittle cornea syndrome. Am. J. Hum. Genet. 2008;82:1217–22.

159. Moraes F, Paye J, Mac Gabhann F, et al. Endothelial cell-dependent regulation of arteriogenesis. Circ. Res. 2013;113:1076–86.

160. Ringpfeil F, McGuigan K, Fuchsel L, et al. Pseudoxanthoma elasticum is a recessive disease characterized by compound heterozygosity. J. Invest. Dermatol. 2006;126:782–6.

161. Ringpfeil F, Nakano A, Uitto J, Pulkkinen L. Compound heterozygosity for a recurrent 16.5-kb Alu-mediated deletion mutation and single-base-pair substitutions in the ABCC6 gene results in pseudoxanthoma elasticum. Am. J. Hum. Genet. 2001;68:642–52.

162. Fischer B, Dimopoulou A, Egerer J, et al. Further characterization of ATP6V0A2-related autosomal recessive cutis laxa. Hum. Genet. 2012;131:1761–73.

163. Sheen VL, Dixon PH, Fox JW, et al. Mutations in the X-linked filamin 1 gene cause periventricular nodular heterotopia in males as well as in females. Hum. Mol. Genet. 2001;10:1775–83.

164. Burda P, Schäfer A, Suormala T, et al. Insights into severe 5,10-methylenetetrahydrofolate reductase deficiency: molecular genetic and enzymatic characterization of 76 patients. Hum. Mutat. 2015;36:611–21.

165. Giunta C, Randolph A, Al-Gazali LI, Brunner HG, Kraenzlin ME, Steinmann B. Nevo syndrome is allelic to the kyphoscoliotic type of the Ehlers-Danlos syndrome (EDS VIA). Am. J. Med. Genet. A 2005;133A:158–64.

166. Yoo HJ, Kim M, Kim M, Chae JS, Lee S-H, Lee JH. The peptidylglycine-α-amidating monooxygenase (PAM) gene rs13175330 A>G polymorphism is associated with hypertension in a Korean population. Hum. Genomics 2017;11:29.

167. Johnson DR. Extra-toes: anew mutant gene causing multiple abnormalities in the mouse. J. Embryol. Exp. Morphol. 1967;17:543–81.

168. Renault M-A, Vandierdonck S, Chapouly C, et al. Gli3 regulation of myogenesis is necessary for ischemia-induced angiogenesis. Circ. Res. 2013;113:1148–58.

169. Li M, He H-P, Gong H-Q, et al. NFATc4 and myocardin synergistically up-regulate the expression of LTCC α1C in ET-1-induced cardiomyocyte hypertrophy. Life Sci. 2016;155:11–20.

170. Le K, Li R, Xu S, et al. PPARα activation inhibits endothelin-1-induced cardiomyocyte hypertrophy by prevention of NFATc4 binding to GATA-4. Arch. Biochem. Biophys. 2012;518:71–8.

171. Shroff N, Ander BP, Zhan X, et al. HDAC9 Polymorphism Alters Blood Gene Expression in Patients with Large Vessel Atherosclerotic Stroke. Transl. Stroke Res. 2019;10:19–25.

172. Wang X-B, Han Y, Sabina S, et al. HDAC9 Variant Rs2107595 Modifies Susceptibility to Coronary Artery Disease and the Severity of Coronary Atherosclerosis in a Chinese Han Population. PLoS One 2016;11:e0160449.

173. Mitsuma W, Kodama M, Hanawa H, et al. Serum endostatin in the coronary circulation of patients with coronary heart disease and its relation to coronary collateral formation. Am. J. Cardiol. 2007;99:494–8.

174. de Juan A, Ince LM, Pick R, et al. Artery-Associated Sympathetic Innervation Drives Rhythmic Vascular Inflammation of Arteries and Veins. Circulation 2019;140:1100–1114.

175. Chang L, Xiong W, Zhao X, et al. Bmal1 in Perivascular Adipose Tissue Regulates Resting-Phase Blood Pressure Through Transcriptional Regulation of Angiotensinogen. Circulation 2018;138:67–79.

176. Liu N, Schoch K, Luo X, et al. Functional variants in TBX2 are associated with a syndromic cardiovascular and skeletal developmental disorder. Hum. Mol. Genet. 2018;27:2454–2465.

177. Xie H, Zhang E, Hong N, et al. Identification of TBX2 and TBX3 variants in patients with conotruncal heart defects by target sequencing. Hum. Genomics 2018;12:44.

178. Lee J, Lee Y, Park B, Won S, Han JS, Heo NJ. Genome-wide association analysis identifies multiple loci associated with kidney disease-related traits in Korean populations. PLoS One 2018;13:e0194044.

179. Kato N, Loh M, Takeuchi F, et al. Trans-ancestry genome-wide association study identifies 12 genetic loci influencing blood pressure and implicates a role for DNA methylation. Nat. Genet. 2015;47:1282–1293.

180. Garside VC, Cullum R, Alder O, et al. SOX9 modulates the expression of key transcription factors required for heart valve development. Development 2015;142:4340–50.

181. Orriols M, Varona S, Martí-Pàmies I, et al. Down-regulation of Fibulin-5 is associated with aortic dilation: role of inflammation and epigenetics. Cardiovasc. Res. 2016;110:431–42.

182. Wang C, Qu B, Wang Z, et al. Proteomic identification of differentially expressed proteins in vascular wall of patients with ruptured intracranial aneurysms. Atherosclerosis 2015;238:201–6.

183. Geng J, Picker J, Zheng Z, et al. Chromosome microarray testing for patients with congenital heart defects reveals novel disease causing loci and high diagnostic yield. BMC Genomics 2014;15:1127.

184. CARDIoGRAMplusC4D Consortium, Deloukas P, Kanoni S, et al. Large-scale association analysis identifies new risk loci for coronary artery disease. Nat. Genet. 2013;45:25–33.

185. Schunkert H, König IR, Kathiresan S, et al. Large-scale association analysis identifies 13 new susceptibility loci for coronary artery disease. Nat. Genet. 2011;43:333–8.

186. Yang W, Ng FL, Chan K, et al. Coronary-Heart-Disease-Associated Genetic Variant at the COL4A1/COL4A2 Locus Affects COL4A1/COL4A2 Expression, Vascular Cell Survival, Atherosclerotic Plaque Stability and Risk of Myocardial Infarction. PLoS Genet. 2016;12:e1006127.

187. Yoneda Y, Haginoya K, Arai H, et al. De novo and inherited mutations in COL4A2, encoding

the type IV collagen α2 chain cause porencephaly. Am. J. Hum. Genet. 2012;90:86–90.

188. Jeanne M, Labelle-Dumais C, Jorgensen J, et al. COL4A2 mutations impair COL4A1 and COL4A2 secretion and cause hemorrhagic stroke. Am. J. Hum. Genet. 2012;90:91–101.

Supplementary Appendix: Author Contributions

K.C., S.P., J.A., C.H., N.S. N. B. and D.A. conceived of and designed the study; A.B., T.W., S.H., D.P., A.A., A.W., N.S., and D.A. acquired and processed the University of Leicester clinical samples; S.H., S.E.I, I.T., L.M., D.W.M., S.D., D.F., R.M.G., and E.G. acquired and processed the Victor Chang Institute clinical samples; K.C., A.B., J.A., T.W., S.H., and D.A. performed the review of clinical phenotypes among putative molecular diagnoses; K.C.; Q.W., D.V., S.H.L and S.D. performed the bioinformatic processing; K.C. performed the statistical analyses with support from A.B., D.V., and S.P.; K.C., A.B., J.A., T.W., S.P., C.H., and D.A. interpreted the results and drafted the manuscript and all authors critically revised the manuscript for important intellectual content.
