# Supplementary material for: Spontaneous Coronary Artery Dissection: Insights on Rare Genetic Variation From Genome Sequencing
Source: Circ Genom Precis Med. 2020 Oct 29;13(6):e003030. doi: 10.1161/CIRCGEN.120.003030 (PMC7748045; doi:10.1161/CIRCGEN.120.003030)
Supplement: Supplementary file 2 [file hcg-13-e003030-s002.pdf]

DOI: 10.1161/CIRCGEN.120.003030

Spontaneous Coronary Artery Dissection: Insights on Rare Genetic Variation from Genome Sequencing

Running title: *Carss et al.; Rare Genetic Variation in SCAD*

Keren J. Carss, PhD[1]; Anna A. Baranowska, MRes[2]; Javier Armisen, PhD[1]; Tom R. Webb, PhD[2]; Stephen E. Hamby, PhD[2]; Diluka Premawardhana, MBBS[2]; Abtehale Al-Hussaini, MBBS[2]; Alice Wood, BSc, MBBS[2]; Quanli Wang, MSc[1]; Sri V. V. Deevi, PhD[1]; Dimitrios Vitsios, PhD[1]; Samuel H. Lewis, PhD[1]; Deevia Kotecha, MBBS[2]; Nabila Bouatia-Naji, PhD[3]; Stephanie Hesselson, PhD[4]; Siiri E. Iismaa, PhD[4,5]; Ingrid Tarr, BSc[4]; Lucy McGrath-Cadell, MBBS, MPH[5]; David W. Muller, MD[4,5]; Sally L. Dunwoodie, PhD[4,5]; Diane Fatkin, MD[4,5,6]; Robert M. Graham, MD[4,5]; Eleni Giannoulatou, DPhil[4,5]; Nilesh J. Samani, MD, FRCP[2]; Slavé Petrovski, PhD[1]; Carolina Haefliger, MD[1]*; David Adlam, DPhil, FRCP[2]*

[1]Centre for Genomics Research, Discovery Sciences, BioPharmaceuticals R&D, AstraZeneca; [2]Department of Cardiovascular Sciences, and NIHR Leicester Biomedical Research Centre, University of Leicester, Leicester, United Kingdom; [3]Université de Paris, Inserm UMR 970 – Paris, Centre de Recherche Cardiovasculaire, Paris, France; [4]Victor Chang Cardiac Research Institute, Darlinghurst; [5]St Vincent's Clinical School, University of NSW Sydney, Kensington; [6]Cardiology Department, St. Vincent's Hospital, Darlinghurst, NSW, Australia
*contributed equally

Circulation: Genomic and Precision Medicine

Correspondence:
Dr David Adlam,
Associate Professor of Acute and Interventional Cardiology
University of Leicester
Department of Cardiovascular Sciences
Glenfield Hospital, Groby Road
Leicester, LE3 9QP
United Kingdom
Tel: +441162044751
Email: da134@le.ac.uk

Journal Subject Terms: Etiology; Genetics; Myocardial Infarction

This article is published in its accepted form; it has not been copyedited and has not appeared in an issue of the journal. Preparation for inclusion in an issue of *Circulation: Genomic and Precision Medicine* involves copyediting, typesetting, proofreading, and author review, which may lead to differences between this accepted version of the manuscript and the final, published version.

Abstract:

Background - Spontaneous coronary artery dissection (SCAD) occurs when an epicardial coronary artery is narrowed or occluded by an intramural hematoma. SCAD mainly affects women and is associated with pregnancy and systemic arteriopathies, particularly fibromuscular dysplasia. Variants in several genes, such as those causing connective tissue disorders, have been implicated; however, the genetic architecture is poorly understood. Here, we aim to better understand the diagnostic yield of rare variant genetic testing among a cohort of SCAD survivors and to identify genes or gene-sets that have a significant enrichment of rare variants.

Methods - We sequenced a cohort of 384 SCAD survivors from the UK, alongside 13,722 UK Biobank controls and a validation cohort of 92 SCAD survivors. We performed a research diagnostic screen for pathogenic variants, and exome-wide and gene-set rare variant collapsing analyses.

Results - The majority of patients within both cohorts are female, 29% of the study cohort and 14% validation cohort have a remote arteriopathy. Four cases across the two cohorts had a diagnosed connective tissue disorder. We identified pathogenic or likely pathogenic variants in seven genes (*PKD1*, *COL3A1*, *SMAD3*, *TGFB2*, *LOX*, *MYLK*, and *YY1AP1*) in 14/384 cases in the study cohort and in 1/92 cases in the validation cohort. In our rare variant collapsing analysis, *PKD1* was the highest ranked gene and several functionally plausible genes were enriched for rare variants, although no gene achieved study-wide statistical significance. Gene-set enrichment analysis suggested a role for additional genes involved in renal function.

Conclusions - By studying the largest sequenced cohort of SCAD survivors we demonstrate that, based on current knowledge, only a small proportion have a pathogenic variant that could explain their disease. Our findings strengthen the overlap between SCAD and renal and connective tissue disorders and we highlight several new genes for future validation.

Key words: spontaneous coronary artery dissection; genetics; sequencing; rare variants; UK biobank

This article is published in its accepted form; it has not been copyedited and has not appeared in an issue of the journal. Preparation for inclusion in an issue of *Circulation: Genomic and Precision Medicine* involves copyediting, typesetting, proofreading, and author review, which may lead to differences between this accepted version of the manuscript and the final, published version.

DOI: 10.1161/CIRCGEN.120.003030

Nonstandard Abbreviations and Acronyms

SCAD: Spontaneous Coronary Artery Dissection

FMD: Fibromuscular dysplasia

CTD: Connective tissue disorder

PCKD: Polycystic kidney disease

EDS: Ehlers-Danlos syndrome

LDS: Loeys-Dietz syndrome

CCDS: Consensus Coding Sequence

SNV: Single nucleotide variant

SV: Structural variant

QC: Qualifying variant

PTV: Protein truncating variant

MTR: Missense tolerance ratio

MAF: Minor allele frequency

Popmax: maximum MAF

P: Pathogenic

LP: Likely pathogenic

Circulation: Genomic and Precision Medicine

Introduction

Spontaneous coronary artery dissection (SCAD) results from the development of an expanding hematoma within the wall of a coronary artery, caused either by haemorrhage in the *tunica media* or possibly an intimal tear and resulting in the development of a false lumen. [1] As the hematoma enlarges, it compresses the true lumen resulting in coronary insufficiency leading to myocardial ischemia, infarction or both and, in some cases, heart failure, and sudden death. Once considered a rare disease, it is now clear that the prevalence has been underestimated [2, 3] and up to 4% of

This article is published in its accepted form; it has not been copyedited and has not appeared in an issue of the journal. Preparation for inclusion in an issue of *Circulation: Genomic and Precision Medicine* involves copyediting, typesetting, proofreading, and author review, which may lead to differences between this accepted version of the manuscript and the final, published version.

patients with an acute coronary syndrome (ST elevation myocardial infarct (STEMI), non-STEMI or unstable angina) who undergo coronary angiography present with SCAD. [4]

SCAD mainly affects young to middle-aged women without an increase in typical cardiovascular risk factors and accounts for up to half of pregnancy-associated myocardial infarction. SCAD has also been associated with multiparity, systemic arteriopathies (particularly fibromuscular dysplasia (FMD)), connective tissue disorders (CTD), inflammatory diseases, and polycystic kidney disease (PCKD), and associated precipitating factors include intensive exercise or emotional stress. [2, 3]

The etiology of SCAD is believed to include a genetic component, although this remains poorly understood. There have been reports of familial SCAD risk [5] and 5-8% of SCAD patients have been found to carry deleterious variants in genes that cause heritable CTDs including *FBN1*, *COL3A1*, and *SMAD3*. [6-8] Of less clear significance, variants in *PKD1*, associated with autosomal dominant PCKD, and *LMX1B*, associated with Nail-patella syndrome, have also been described in SCAD. [7] More recently, variants in *TSR1* and *TLN1* have been reported in familial and sporadic SCAD cases [9, 10]. Additionally, common variants including in *PHACTR1/EDN1* have been associated with SCAD, [11] highlighting the genetic and etiological heterogeneity of the condition.

Sequencing enables the examination of rare variation to better understand its contribution to the genetic architecture of SCAD. The objectives of this study were to leverage genome sequencing data generated on SCAD survivors, and exome sequence data from appropriate UK biobank controls to: (i) better understand the diagnostic yield of rare variants in the largest sequenced cohorts of SCAD-survivors, (ii) gain insight into the genetic architecture of SCAD by

Circulation: Genomic and Precision Medicine

This article is published in its accepted form; it has not been copyedited and has not appeared in an issue of the journal. Preparation for inclusion in an issue of *Circulation: Genomic and Precision Medicine* involves copyediting, typesetting, proofreading, and author review, which may lead to differences between this accepted version of the manuscript and the final, published version.

identifying genes or gene-sets with an excess of rare variants in the case/control populations, and (iii) identify biologically plausible genes that could be candidates for further validation.

Methods

Methods are provided in the Supplementary Material. The data that support the findings of this study are available from the corresponding author upon reasonable request. The UK SCAD cohort was approved by the UK National Research Ethics Service (14/EM/0056) and the UK Health Research Authority and conducted in accordance with the Declaration of Helsinki. All patients provided signed informed consent prior to the study start. The Victor Chang Cardiac Research Institute SCAD cohort was approved by the St Vincent's Human Research Ethics Committee (2019/ETH03171) and conducted in accordance with the National Health and Medical Research Council's National Statement on Ethical Conduct in Human Research and the CPMP/ICH Note for Guidance on Good Clinical Practice. All patients provided informed consent prior to the start of the study. Controls for association analyses were selected from UK Biobank participants after screening for cardiovascular disease.

Circulation: Genomic and Precision Medicine

Results

Patient cohorts

The SCAD survivors sequenced for this study consists of 384 patients from the UK SCAD registry and 92 patients from the Victor Chang Cardiac Research Institute SCAD cohort. All patients had angiographically confirmed diagnosis of SCAD. The clinical characteristics of the SCAD patients are described in Table 1. The majority of patients are females of European ancestry with a single SCAD event. 29.17% of the UK SCAD cohort and 14% of the Victor

This article is published in its accepted form; it has not been copyedited and has not appeared in an issue of the journal. Preparation for inclusion in an issue of *Circulation: Genomic and Precision Medicine* involves copyediting, typesetting, proofreading, and author review, which may lead to differences between this accepted version of the manuscript and the final, published version.

Chang Cardiac Research Institute SCAD cohort have a remote arteriopathy, including dilations, dissections, aneurysms and fibromuscular dysplasia, in another vascular bed. Only three individuals in the UK SCAD cohort and one patient in the Victor Chang Cardiac Research Institute SCAD cohort have a CTD.

Pathogenic variants were identified in 3.6% of SCAD patients in the UK cohort

We assessed genes previously reported in SCAD patients or related conditions (tier 1 and tier 2 gene lists as described in Supplemental Methods and Supplementary Table 1) and identified 15 different pathogenic or likely pathogenic variants according to ACMG guidelines [12] in 14/384 (3.6%) cases (Table 2). At least 99% of the SCAD cases have read depth ≥10X for ≥98% of the Consensus Coding Sequence (CCDS) of the six tier 1 genes (*COL3A1*, *FBN1*, *PKD1*, *SMAD3*, *TLN1*, and *TSR1*) (Supplementary Figure 1), suggesting adequate coverage to detect protein coding single nucleotide variants (SNVs) and indels in these genes. Nine individuals had variants in tier 1 genes (*COL3A1* n=2, *PKD1* n=5, *SMAD3* n=2) and five individuals with variants in tier 2 genes (*LOX* n=1, *MYLK* n=1, *TGFB2* n=2, *YY1AP1* n=1). One of our SCAD cases has two heterozygous variants in *YY1AP1*. Homozygous or compound heterozygous PTVs in *YY1AP1* can cause Grange syndrome, which is characterized by severe, early onset vaso-occlusive disease and FMD-like vascular features, brachydactyly, syndactyly, fragile bones and learning disabilities, [14] however, we have been unable to phase the two heterozygous *YY1AP1* PTVs in the patient. Of the 15 different pathogenic/likely pathogenic alleles, 11 (73%) have previously been reported in SCAD or a related condition in HGMD or ClinVar, and four (two in *PKD1* and two in the single *YY1AP1* case) are novel PTVs reported here for the first time.

There were an additional 19 cases where a single heterozygous variant was identified in a recessive tier 1 or 2 gene, including three cases with structural variants (SVs) (Supplementary

Circulation: Genomic and Precision Medicine

This article is published in its accepted form; it has not been copyedited and has not appeared in an issue of the journal. Preparation for inclusion in an issue of *Circulation: Genomic and Precision Medicine* involves copyediting, typesetting, proofreading, and author review, which may lead to differences between this accepted version of the manuscript and the final, published version.

Figure 2). These were not considered pathogenic or likely pathogenic because they were not identified in biallelic form (Supplementary Table 2).

We did not identify any pathogenic or likely pathogenic SVs. However, we did identify a heterozygous deletion (GRCh38.10:52048268-52058807del; 11kb) in ScPt0668423L that causes an in-frame deletion of exon 6 of *PRKG1*. The deletion is absent in gnomAD v2 SVs, Decipher, and ClinVar, and a clear drop in coverage is visible on examination of the reads (Supplementary Figure 2).

We sought to further investigate the seven genes in which we identified pathogenic/likely pathogenic variants in the UK cohort in an independent Victor Chang Cardiac Research Institute cohort of 92 sporadic SCAD cases. Among these seven genes we identified a single putatively pathogenic PTV in *COL3A1* (2-189004115-A-AG, ENST00000304636: c.2798dupG (p.Ser934fs)).

American Heart Association.
Circulation: Genomic and Precision Medicine

Clinical features of SCAD patients with identified pathogenic variants

Clinical details of the 14 cases carrying pathogenic or likely pathogenic variants are provided in Supplementary Table 3. Two (ScPt0150875X and ScPt0395467Z) of the five cases with *PKD1* variants, both PTVs, have PCKD. The mother of ScPt0395467Z has PCKD and hypermobility and their maternal grandmother had PCKD and died due to ruptured berry aneurysm. ScPt0150875X has no family history of PCKD, cardiovascular, other than hypertension and hypercholesterolemia, or CTDs. One case (ScPt0743044L) with a likely pathogenic missense variant in *PKD1* has hypermobility with Ehlers-Danlos syndrome (EDS) like features, reports easy bruising and has a family history of SCAD (1st cousin) and hypermobility (son). Of the remaining two patients, one (ScPt0899224S) has an aortic root diameter at the upper limit of normal, neither patient has any other SCAD associated phenotype or relevant family history.

This article is published in its accepted form; it has not been copyedited and has not appeared in an issue of the journal. Preparation for inclusion in an issue of *Circulation: Genomic and Precision Medicine* involves copyediting, typesetting, proofreading, and author review, which may lead to differences between this accepted version of the manuscript and the final, published version.

Notably, one other participant in the UK SCAD cohort and one patient in the Victor Chang Cardiac Research Institute SCAD cohort have PCKD and missense variants of uncertain significance in *PKD1* (16-2100038-A-G, ENST00000262304: c.9746T>C (p.Leu3249Pro) and 16- 2103514-A-G, ENST00000262304: c.8543T>C (p.Val2848Ala) respectively).

Neither case (BPt00521469 and ScPt0162409J) with *COL3A1* variants has typical characteristics of vascular EDS. BPt00521469 has high palate and pes planus and has one male sibling with patella dislocation and another with recurrent pneumothorax. ScPt0162409J has subconjunctival haemorrhage and both she and her mother have scoliosis.

One patient (ScPT0443400X) with a *TGFB2* variant, which is mutated in Loeys-Dietz syndrome (LDS), has hypermobility, Chiari malformation (mother and sister also affected) and reports easily bruising. The other case (ScPt0698633H) with likely pathogenic *TGFB2* variant has remote arteriopathies including left carotid artery dissection and right internal carotid aneurysm, and no family history of disease. The two patients with *SMAD3* variants, which also causes LDS) have not yet been screened for remote arteriopathies. Both have a family history of aortic aneurysm.

Circulation: Genomic and Precision Medicine

Our patient with a *LOX* variant also had right internal carotid dissection and FMD and their mother died due to intracerebral bleed secondary to aneurysm. The patient with a pathogenic variant in *MYLK* has dyslipidemia and systemic inflammatory disease and no relevant family history. The case with two *YY1AP1* variants has FMD, renal artery stenosis, brachydactyly, and migraines.

We could identify no significant differences between the 14 cases with pathogenic or likely pathogenic variants and the remainder of cases in terms of their age, recurrence, and

This article is published in its accepted form; it has not been copyedited and has not appeared in an issue of the journal. Preparation for inclusion in an issue of *Circulation: Genomic and Precision Medicine* involves copyediting, typesetting, proofreading, and author review, which may lead to differences between this accepted version of the manuscript and the final, published version.

DOI: 10.1161/CIRCGEN.120.003030

several other clinical endpoints including remote arteriopathies and hypermobility (Supplementary Table 4).

The patient with a deletion of *PRKG1* exon 6 had no other notable clinical characteristics besides a single SCAD event.

Gene-level collapsing analysis

To identify genes enriched for rare variants in SCAD cases in the UK cohort compared to controls, we used gene-level collapsing analysis. [15-17] Cases are the subset who are of European ancestry, are unrelated, and pass quality control filters (n=357). For controls, we used exome sequencing data from 13,722 individuals from the UK Biobank who had high-quality exome sequencing data, were unrelated, of European ancestry, and had no report of a relevant disease (Supplementary Table 5). We ran eleven different collapsing analysis models with different definitions of qualifying variants (QVs), each designed to capture slightly different genetic architecture (Supplementary Methods and Table 3).

No association reached study-wide significance (Table 4, Supplementary Table 6, and Supplementary Figure 3). One of the highest ranked associations was *PKD1*, which was the highest ranked gene in the ultra-rare damaging missense tolerance ratio (MTR) model ($p=7.3 \times 10^{-6}$). The association for the ultra-rare damaging model is weaker (p=0.0018), demonstrating that variants in regions of *PKD1* that are intolerant to missense variation are more likely to be associated with SCAD (Supplementary Table 7). The *PKD1* signal could be considered significant upon restricting the search-space and thus multiple-testing correction to the top decile (n=1,928) highest expressed genes in the coronary artery tissue data from the GTEX database (accessed 19/11/2019). [18]

This article is published in its accepted form; it has not been copyedited and has not appeared in an issue of the journal. Preparation for inclusion in an issue of *Circulation: Genomic and Precision Medicine* involves copyediting, typesetting, proofreading, and author review, which may lead to differences between this accepted version of the manuscript and the final, published version.

DOI: 10.1161/CIRCGEN.120.003030

Prioritisation of non-significant genes from collapsing analysis results by manual review and automated machine learning

Although no association reached study-wide significance, we hypothesized that within the highly ranked results may be genes in which rare variants do increase the risk of SCAD, but our current study is underpowered to highlight them. Therefore, we further investigated highly ranked results with the aim of identifying a shortlist of genes not previously associated with SCAD but are functionally plausible and could be further investigated in future larger SCAD studies. We employed two complementary approaches: manual review alongside an automated machine-learning approach. Genes prioritized by the manual approach (which was conducted blind to the results of the automated machine-learning method) include *PAM, GLI3, SEC24B, COL18A1, NFATC4, ARNTL, TBX2, HDAC9, SOX9, SORBS2 and COL4A2*; all implicated in blood pressure regulation and cardiovascular system development and morphology (Supplementary Table 8).

Circulation: Genomic and Precision Medicine

Although the manual approach is flexible and thorough, it requires substantial expertise in the phenotype and can be laborious. Thus, we also employed mantis-ml [19], a machine-learning method for gene prioritisation. We trained mantis-ml on SCAD tier 1 and 2 genes to identify which of the remaining genes share characteristics most commonly with those genes. During the application on SCAD tier 1 and 2 genes, mantis-ml predictions were primarily driven by disease/phenotype-specific mouse knockout models, protein-protein interactions with known SCAD-associated genes, gene expression in heart and aorta, GWAS hits and heart-associated Gene Ontology terms (Supplementary Figure 4).

To assess whether the top ranked genes from the collapsing analysis were preferentially enriched for the top mantis-ml predictions we performed multiple hypergeometric tests between

This article is published in its accepted form; it has not been copyedited and has not appeared in an issue of the journal. Preparation for inclusion in an issue of *Circulation: Genomic and Precision Medicine* involves copyediting, typesetting, proofreading, and author review, which may lead to differences between this accepted version of the manuscript and the final, published version.

DOI: 10.1161/CIRCGEN.120.003030

the top 5% mantis-ml predictions and the top gene hits from collapsing analysis ($p<0.05$) for different types of QVs. We observed that the top ranked genes from the “ultra-rare variant” collapsing analysis were significantly enriched for the top 5% mantis-ml predictions (Supplementary Figure 5a). There was no significant enrichment when adopting the “synonymous variant” collapsing analysis model (Supplementary Figure 5b), suggesting that mantis-ml’s predictions are likely pointing towards the top-ranked genes from collapsing analyses that are likely to be SCAD risk genes.

Mantis-ml yielded a consensus list of ten genes that are highly ranked in the collapsing analysis for “ultra-rare” variants (Table 4 and Supplementary Figure 5c). All ten consensus predictions from mantis-ml were also prioritized by the manual approach.

Circulation: Genomic and Precision Medicine

Gene-set enrichment analysis

We next explored gene-set enrichment among 9,339 pre-defined gene-sets, including our SCAD gene lists. Only the SCAD tier 1 gene-set ($p=3.6x10^{-7}$, comprising six genes) reached the Bonferroni-corrected significance threshold of $p<5.4x10^{-7}$; a key positive control demonstrating that there is clear statistical enrichment of damaging variants in tier 1 genes among SCAD cases compared to controls. This signal is driven by six individuals with QVs in *PKD1*, three in *COL3A1*, and two in *SMAD3*. The gene-set with the second lowest p-value is Loop of Henle development genes ($p=2.4x10^{-6}$, comprising 11 genes), driven by the same six individuals with QVs in *PKD1*, two in *HES5*, two in *UMOD*, and one in *DLL1* (Supplementary Tables 7 and 9, and Supplementary Figure 6). Importantly, for these two gene-sets, upon excluding individuals with *PKD1* QVs, both remained highly ranked albeit no longer significant (for SCAD tier 1 genes p=0.016 (122/9339 gene-sets) and for Loop of Henle development genes p=0.04

This article is published in its accepted form; it has not been copyedited and has not appeared in an issue of the journal. Preparation for inclusion in an issue of *Circulation: Genomic and Precision Medicine* involves copyediting, typesetting, proofreading, and author review, which may lead to differences between this accepted version of the manuscript and the final, published version.

(268/9339 gene-sets)). Thus, while the signals observed in these gene-sets are clearly primarily driven by *PKD1*, there appear to be suggestive signal from the remaining genes in the gene-sets.

Discussion

This study is the largest analysis to date of Mendelian-like rare genetic variants that might be responsible for SCAD. We have assessed the entire coding genome, rather than solely applying a candidate approach and have investigated the contribution of rare genetic variants to SCAD by analysing genomes of a large cohort of SCAD-survivors for pathogenic variants and performing rare variant collapsing analyses. We identified variants deemed pathogenic or likely pathogenic for CTDs and PCKD, including in genes not previously reported in SCAD. Our findings strengthen the evidence that SCAD is an occasional clinical outcome in these conditions with implications for both clinical and genetic screening of SCAD patients. We also identified several new genes enriched for rare variants that require validation in larger future studies.

American Heart Association.
Circulation: Genomic and Precision Medicine

Overall, we identified variants that might be responsible for SCAD in 14/384 (3.6%) cases in the study cohort, which is in line with expectation from smaller studies.[6, 7] Importantly, for controls we used exome sequencing data from the UK Biobank. The size of this cohort along with available phenotypic data allowed us to apply strict selection criteria, providing a major advantage over the 'controls of convenience' used by many previous rare-variant studies. As such, our identification of pathogenic or likely pathogenic variants probably represents an accurate reflection of the genetic burden of rare variants in SCAD and provides a better understanding of the genetic component of disease.

These findings have important implications for patient management. The role of clinical genetic testing in SCAD-survivors has been uncertain. Our results support the hypothesis that

This article is published in its accepted form; it has not been copyedited and has not appeared in an issue of the journal. Preparation for inclusion in an issue of *Circulation: Genomic and Precision Medicine* involves copyediting, typesetting, proofreading, and author review, which may lead to differences between this accepted version of the manuscript and the final, published version.

rare variants are likely causal in only a small subpopulation of SCAD cases, some with clinical features or a family history of CTDs or PCKD, suggesting that although these represent an important and pathophysiologically informative group, the yield from routine clinical genetic screening based on our current knowledge of SCAD genetics would be low. The combination of careful clinical phenotyping (including assessment for typical changes in the palate, skin and facial features as well as musculoskeletal abnormalities [20,21]), remote arteriopathy cross sectional imaging from brain to pelvis (which will necessarily include renal imaging) and assessment of family history, to include CTDs and PCKD, will identify most patients with pathogenic variants for further genetic assessment. Our data suggest a small number of patients with pathogenic variants will still be missed by this approach. However, given the rarity of such patients, the psychological morbidity of genetic screening and the lack of genotype-specific effective medical interventions, the merit of routine genetic screening of all SCAD-survivors is debatable.

Circulation: Genomic and Precision Medicine

Five of our SCAD patients had pathogenic or likely pathogenic in *PKD1*, and *PKD1* was also one of the highest ranked associations from the collapsing analysis. Two additional SCAD patients were also noted with PCKD and *PKD1* missense variants of uncertain significance. Pathogenic *PKD1* variants have previously been reported in SCAD patients, [7, 22] and the observation here highlights that the co-occurrence of SCAD and *PKD1* dysfunction is moving beyond being merely anecdotal, though notably, not every SCAD patient with *PKD1* variants had polycystic kidneys. Polycystin 1 has been implicated in the structural integrity of blood vessels, providing a plausible genotype-phenotype mechanism for SCAD and potentially a useful paradigm to aid understanding of the coronary biomechanical processes leading to SCAD. [23,24] The involvement of *PKD1* but not *PKD2* may be explained by the known milder phenotype, especially in females of *PKD2* where renal disease occurs later and fewer intracranial aneurysms

This article is published in its accepted form; it has not been copyedited and has not appeared in an issue of the journal. Preparation for inclusion in an issue of *Circulation: Genomic and Precision Medicine* involves copyediting, typesetting, proofreading, and author review, which may lead to differences between this accepted version of the manuscript and the final, published version.

are reported. The population prevalence of *PKD2* disease variants is also lower than for *PKD1*. [25-27] Gene-set analysis identified Loop of Henle development genes, driven by individuals with QVs in *PKD1*, *UMOD*, *HES5*, and *DLL1*, suggesting that the association between SCAD events and renal dysfunction may be more extensive than has been recognized. Experiments in mice would support a role for these genes in SCAD pathogenesis via a direct effect on vessel development or maintenance. [28-29]

We also found pathogenic variants in *COL3A1* and *SMAD3*, which respectively cause vascular EDS, and LDS, and have previously been identified in multiple SCAD patients. [6, 7] We also detected variants in *TGFB2*, which is also mutated in LDS, and *MYLK*, where variants are associated with aortic dissection and FMD, where variants have been described in single SCAD patients. [30] These are the first reported SCAD patients with variants in *LOX* and *YY1AP1*. *LOX* encodes an enzyme that cross-links fibres in connective tissue matrices, and PTVs in this gene cause thoracic aortic aneurysms and dissections [31], while homozygous or compound heterozygous PTVs in *YY1AP1* can cause Grange syndrome. [13] As with our patients with *PKD1* variants, phenotypic expressivity of patients with variants in these genes was variable, with patient not always having classical clinical features typical of vascular EDS (*COL3A1*) or LDS (*SMAD3, TGFB2*). This lack of phenotypic concordance suggests selecting sub-sets of SCAD patients for genetic screening based on associated clinical phenotypes would miss some patients with mutations in those genes.

Although we did not find any pathogenic SVs, we did detect an in-frame deletion of *PRKG1* exon 6. *PRKG1* is associated with autosomal dominant familial aortic aneurysm, and the mechanism is thought to be gain-of-function. [32] It remains possible that the deletion in this

American Heart Association.
Circulation: Genomic and Precision Medicine

This article is published in its accepted form; it has not been copyedited and has not appeared in an issue of the journal. Preparation for inclusion in an issue of *Circulation: Genomic and Precision Medicine* involves copyediting, typesetting, proofreading, and author review, which may lead to differences between this accepted version of the manuscript and the final, published version.

DOI: 10.1161/CIRCGEN.120.003030

SCAD patient produces a gain-of-function as it deletes a single, small, in-frame exon, but this would require further functional studies.

We prioritized a total of 11 genes highly ranked in the collapsing analysis, namely, *PAM, GLI3, SEC24B, COL18A1, NFATC4, ARNTL, TBX2, HDAC9, SOX9, SORBS2* and *COL4A2*, for future validation. Each of these genes represent a credible candidate for SCAD, based on function, expression and mouse phenotype, but require validation in future studies. Ten of these genes (bar *PAM)* were also prioritized by our machine learning tool mantis-ml, demonstrating the complementarity of the approaches and confirming mantis-ml as a promising addition to the gene prioritisation toolbox.

Limitations

Given the low frequency and genetic heterogeneity of SCAD, our study is relatively under-powered for novel gene discovery. Furthermore, while we utilized the power of genome sequencing to some extent (i.e. by investigating deletions), several classes of variation were beyond the scope of this study including novel clinically relevant non-coding variants, more complex structural variants, and the contribution of common variants including polygenic risk. Finally, the SCAD cohort recruited at the Victor Chang Cardiac Research Institute was only adopted for reviewing additional variants in the subset of tier 1 and 2 genes where variants had been found in the UK cohort.

American Heart Association.
Circulation: Genomic and Precision Medicine

Conclusion

We have demonstrated that only ~3.6% of SCAD-survivors have a pathogenic variant that is likely responsible for their phenotype. Our study supports previous reports of a connection between *PKD1* and SCAD, indicating a statistically confident association. Moreover, gene-set enrichment analyses suggest the relationship might extend beyond *PKD1* to other renal disease

This article is published in its accepted form; it has not been copyedited and has not appeared in an issue of the journal. Preparation for inclusion in an issue of *Circulation: Genomic and Precision Medicine* involves copyediting, typesetting, proofreading, and author review, which may lead to differences between this accepted version of the manuscript and the final, published version.

genes. The repertoire of CTD genes reported to be involved in SCAD may also be higher than previously thought, as suggested in this study by the identification of patients with pathogenic variants in *TGFB2*, *LOX*, *MYLK*, and *YY1AP1*. We anticipate that the growing catalogue of candidate SCAD risk alleles we have identified here could assist in delineating meaningful genetic endotypes although the overall contribution of rare variants to disease is small.

Acknowledgments: We acknowledge the leadership of the ESC-ACCA SCAD Study Group. We thank SCAD study participants and the participants and investigators in the UK biobank study (Resource Application Number 26041), and UK and Australian clinical colleagues who have referred SCAD cases. We acknowledge Jenny Middleton, Jane Plume, Donna Alexander, Sue Sterland, Daniel Lawday, Emma Beeston, Ellie Clarke, Tara Maitland Andrea Marshall, Pamella Mackenzie, Sarah Ford and Liz Stern. The views presented in this paper are those of the authors and not the NHS, department of health or NIHR. We thank the AstraZeneca Centre for Genomics Research Analytics and Informatics team for processing and analysis of sequencing data.

Sources of Funding: Sequencing was funded by AstraZeneca's Centre for Genomics Research, Discovery Sciences, BioPharmaceuticals R&D, and grants from the Cardiac Society of Australia and New Zealand, National Health and Medical Research Council, Australia (Grant number APP1161200; SEI & RMG), St Vincent's Clinic Foundation (RMG), Catholic Archdiocese of Sydney (RMG), Perpetual Philanthropy (RMG), NSW Health CVD Clinician-Scientist Grant (RMG), and SCAD Research Inc. The UK SCAD study was supported by the British Heart Foundation (BHF) PG/13/96/30608, the NIHR rare disease translational collaboration, the Leicester NIHR Biomedical Research Centre and BeatSCAD. NB is supported by a European grant from the European Commission (ERC-Stg-ROSALIND-716628).

Disclosures: KC, JA, QW, SD, DV, SHL, SP and CH are employees of AstraZeneca. DA has received research funding from Abbott vascular inc. to support a clinical research fellow and has undertaken unrelated consultancy for GE inc. No other authors have potential conflicts of interest to declare.

References:

1. Jackson R, Al-Hussaini A, Joseph S, van Soest G, Wood A, Macaya F, Gonzalo N, Cade J, Caixeta A, Hlinomaz O, et al. Spontaneous Coronary Artery Dissection: Pathophysiological Insights From Optical Coherence Tomography. *JACC Cardiovasc Imaging.* 2019;12:2475-2488.

This article is published in its accepted form; it has not been copyedited and has not appeared in an issue of the journal. Preparation for inclusion in an issue of *Circulation: Genomic and Precision Medicine* involves copyediting, typesetting, proofreading, and author review, which may lead to differences between this accepted version of the manuscript and the final, published version.

2. Adlam D, Alfonso F, Maas A, Vrints C, Writing C. European Society of Cardiology, acute cardiovascular care association, SCAD study group: a position paper on spontaneous coronary artery dissection. *Eur Heart J*. 2018;39:3353-3368.

3. Hayes SN, Kim ESH, Saw J, Adlam D, Arslanian-Engoren C, Economy KE, Ganesh SK, Gulati R, Lindsay ME, Mieres JH, et al. Spontaneous Coronary Artery Dissection: Current State of the Science: A Scientific Statement From the American Heart Association. *Circulation*. 2018;137:e523-e557.

4. Nishiguchi T, Tanaka A, Ozaki Y, Taruya A, Fukuda S, Taguchi H, Iwaguro I, Ueno S, Okumoto Y, Akasaka T. Prevalence of spontaneous coronary artery dissection in patients with acute coronary syndrome. *Eur Heart J Acute Cardiovasc Care*. 2016;5:263-70.

5. Goel K, Tweet M, Olson TM, Maleszewski JJ, Gulati R, Hayes SN. Familial spontaneous coronary artery dissection: evidence for genetic susceptibility. *JAMA Intern Med*. 2015;175:821-6.

6. Henkin S, Negrotto SM, Tweet MS, Kirmani S, Deyle DR, Gulati R, Olson TM, Hayes SN. Spontaneous coronary artery dissection and its association with heritable connective tissue disorders. *Heart*. 2016;102:876-81.

7. Kaadan MI, MacDonald C, Ponzini F, Duran J, Newell K, Pitler L, Lin A, Weinberg I, Wood MJ, Lindsay ME. Prospective Cardiovascular Genetics Evaluation in Spontaneous Coronary Artery Dissection. *Circ Genom Precis.* Med 2018;11:e001933.

8. von Hundelshausen P, Oexle K, Bidzhekov K, Schmitt MM, Hristov M, Blanchet X, Kaemmerer H, Matyas G, Meitinger T, Weber C. Recurrent spontaneous coronary dissections in a patient with a de novo fibrillin-1 mutation without Marfan syndrome. *Thromb Haemost*. 2015;113:668-70.

9. Sun Y, Chen Y, Li Y, Li Z, Li C, Yu T, Xiao L, Yu B, Zhao H, Tao M, et al. Association of TSR1 Variants and Spontaneous Coronary Artery Dissection. *J Am Coll Cardiol*. 2019;74:167-176.

10. Turley TN, Theis JL, Sundsbak RS, Evans JM, O'Byrne MM, Gulati R, Tweet MS, Hayes SN, Olson TM. Rare Missense Variants in TLN1 Are Associated With Familial and Sporadic Spontaneous Coronary Artery Dissection. *Circ Genom Precis Med*. 2019;12:e002437.

11. Adlam D, Olson TM, Combaret N, Kovacic JC, Iismaa SE, Al-Hussaini A, O'Byrne MM, Bouajila S, Georges A, Mishra K, et al. Association of the PHACTR1/EDN1 Genetic Locus With Spontaneous Coronary Artery Dissection. *J Am Coll Cardiol*. 2019;73:58-66.

12. Richards S, Aziz N, Bale S, Bick D, Das S, Gastier-Foster J, Grody WW, Hegde M, Lyon E, Spector E, et al. Standards and guidelines for the interpretation of sequence variants: a joint consensus recommendation of the American College of Medical Genetics and Genomics and the Association for Molecular Pathology. *Genet Med*. 2015;17:405-24.

This article is published in its accepted form; it has not been copyedited and has not appeared in an issue of the journal. Preparation for inclusion in an issue of *Circulation: Genomic and Precision Medicine* involves copyediting, typesetting, proofreading, and author review, which may lead to differences between this accepted version of the manuscript and the final, published version.

13. Verstraeten A, Perik MHAM, Baranowska AA, Meester JAN, Ven Den Heuvel L, Bastianen J, Kempers M, Krapels IPC, Maas A, Rideout A, et al. Enrichment of rare variants in Loeys-Dietz syndrome genes in spontaneous coronary artery dissection but not in sever fibromuscular displasia. *Circulation*. 2020;142:1021-1024.

14. Guo DC, Duan XY, Regalado ES, Mellor-Crummey L, Kwartler CS, Kim D, Lieberman K, de Vries BBA, Pfundt R, Schinzel A, et al. Loss-of-Function Mutations in YY1AP1 Lead to Grange Syndrome and a Fibromuscular Dysplasia-Like Vascular Disease. *Am J Hum Genet*. 2017;100:21-30.

15. Cameron-Christie S, Wolock CJ, Groopman E, Petrovski S, Kamalakaran S, Povysil G, Vitsios D, Zhang M, Fleckner J, March RE, et al. Exome-Based Rare-Variant Analyses in CKD. *J Am Soc Nephrol*. 2019;30:1109-1122.

16. Cirulli ET, Lasseigne BN, Petrovski S, Sapp PC, Dion PA, Leblond CS, Couthouis J, Lu YF, Wang Q, Krueger BJ, et al. Exome sequencing in amyotrophic lateral sclerosis identifies risk genes and pathways. *Science.* 2015;347:1436-41.

17. Petrovski S, Todd JL, Durheim MT, Wang Q, Chien JW, Kelly FL, Frankel C, Mebane CM, Ren Z, Bridgers J, et al. An Exome Sequencing Study to Assess the Role of Rare Genetic Variation in Pulmonary Fibrosis. *Am J Respir Crit Care Med.* 2017;196:82-93.

18. GTEx Consortium. The Genotype-Tissue Expression (GTEx) Project. *Nat Genet.* 2013;45:580-5.

19. Vitsios D, Petrovski S. Stochastic semi-supervised learning to prioritise genes from high-throughput genomic screens. *bioRxiv*.

20. Malfait F, Francomano C, Byers P, Belmont J, Berglund B, Black J, Bloom L, Bowen JM, Brady AF, Burrows NP, et al. The 2017 international classification of the Ehlers-Danlos syndromes. *Am J Med Genet C Semin Med Genet*. 2017;175:8-26.

21. Meester JAN, Verstraeten A, Schepers D, Alaerts M, Van Laer L, Loeys BL. Differences in manifestations of Marfan syndrome, Ehlers-Danlos syndrome, and Loeys-Dietz syndrome. *Ann Cardiothorac Surg*. 2017;6:582-594.

22. Klingenberg-Salachova F, Limburg S, Boereboom F. Spontaneous coronary artery dissection in polycystic kidney disease. *Clin Kidney J.* 2012;5:44-6.

23. Varela A, Piperi C, Sigala F, Agrogiannis G, Davos CH, Andri MA, Manopoulos C, Tsangaris S, Basdra EK, Papavassiliou AG. Elevated expression of mechanosensory polycystins in human carotid atherosclerotic plaques: association with p53 activation and disease severity. *Sci Rep*. 2015;5:13461.

Circulation: Genomic and Precision Medicine

This article is published in its accepted form; it has not been copyedited and has not appeared in an issue of the journal. Preparation for inclusion in an issue of *Circulation: Genomic and Precision Medicine* involves copyediting, typesetting, proofreading, and author review, which may lead to differences between this accepted version of the manuscript and the final, published version.

24. Hassane S, Claij N, Lantinga-van Leeuwen IS, Van Munsteren JC, Van Lent N, Hanemaaijer R, Breuning MH, Peters DJM, DeRuiter MC. Pathogenic sequence for dissecting aneurysm formation in a hypomorphic polycystic kidney disease 1 mouse model. *Arterioscler Thromb Vasc Biol*. 2007;27:2177-83.

25. Demetriou K, Tziakouri C, Anninou K, Eleftheriou A, Koptides M, Nicolaou A, Deltas CC, Pierides A. Autosomal dominant polycystic kidney disease-type 2. Ultrasound, genetic and clinical correlations. *Nephrol Dial Transplant* 2000;15:205-11.

26. Hateboer N, v Dijk MA, Bogdanova N, Coto E, Saggar-Malik AK, San Millan JL, Torra R, Breuning M, Ravine D. Comparison of phenotypes of polycystic kidney disease types 1 and 2. European PKD1-PKD2 Study Group. *Lancet* 1999;353:103-7

27. Torra R, Badenas C, Darnell A, Nicolau C, Volpini V, Revert L, Estivill X. Linkage, clinical features, and prognosis of autosomal dominant polycystic kidney disease types 1 and 2. *J Am Soc Nephrol* 1996;7:2142-51.

28. Kitagawa M, Hojo M, Imayoshi I, Goto M, Ando M, Ohtsuka T, Kageyama R, Miyamoto S. Hes1 and Hes5 regulate vascular remodeling and arterial specification of endothelial cells in brain vascular development. *Mech Dev*. 2013;130:458-66.

29. Sorensen I, Adams RH, Gossler A. DLL1-mediated Notch activation regulates endothelial identity in mouse fetal arteries. *Blood*. 2009;113:5680-8.

30. L Giuliani, A Di Toro, E Disabella, M Grasso, A Serio, M Urtis, A Pilotto, A Repetto, A Valentini, F Calliada, et al. P5539 Genetic heterogeneity of spontaneous coronary artery dissection (SCAD). *European Heart Journal*;40. Abstract.

31. Guo DC, Regalado ES, Gong L, Duan X, Santos-Cortez RL, Arnaud P, Ren Z, Cai B, Hostetler EM, Moran R, et al. LOX Mutations Predispose to Thoracic Aortic Aneurysms and Dissections. *Circ Res*. 2016;118:928-34.

32. Guo DC, Regalado E, Casteel DE, Santos-Cortez RL, Gong L, Kim JJ, Dyack S, Horne SG, Chang G, Jondeau G, et al. Recurrent gain-of-function mutation in PRKG1 causes thoracic aortic aneurysms and acute aortic dissections. *Am J Hum Genet*. 2013;93:398-404.

American Heart Association.
Circulation: Genomic and Precision Medicine

This article is published in its accepted form; it has not been copyedited and has not appeared in an issue of the journal. Preparation for inclusion in an issue of *Circulation: Genomic and Precision Medicine* involves copyediting, typesetting, proofreading, and author review, which may lead to differences between this accepted version of the manuscript and the final, published version.

DOI: 10.1161/CIRCGEN.120.003030

Table 1. SCAD patient characteristics

Feature	University of Leicester cohort	Victor Chang Cardiac Research Institute cohort
Unrelated to any other SCAD case (n)	384	92
Female (%, n)	94.27% (362)	91.3% (84)
Self-reported ethnicity (%)	White British 89.58% (344)<br>White Irish 1.30% (5)<br>Any other white background 4.17% (16)<br>Black or Black British African 0.78% (3)<br>Chinese 0.26% (1)<br>Asian or Asian British Pakistani 0.78% (3)<br>Asian or Asian British Indian 2.08% (8)<br>Mixed White and Asian 0.26% (1)<br>Mixed White and Black Caribbean 0.26% (1)<br>any other ethnic group 0.26% (1)<br>Any other Asian 0.26% (1)	White 85.87%<br>Middle Eastern 1.09%<br>Mixed white and Middle Eastern 2.17%<br>Mixed white and African 2.17%<br>Mixed white and Maori 1.09%<br>Mixed white and Aboriginal 2.17%
BMI (mean, range)	26.33 (16-59)<br>Not known 1	26.229 (17-46.3)<br>Not known 2
Age at first SCAD event (mean, range)	46.89 (25-71)	45.71 (24 – 69)
Known connective tissue disorder (n)	No CTD 99.2% (381)<br>CTD 0.8% (3)	No CTD / not known 98.9% (91)<br>CTD 1.09% (1)
Polycystic kidney disease (n)	No PCKD 99.2% (381)<br>PCKD 0.8% (3)	No PCKD 98.9% (91)<br>PCKD 1.09 (1)
Total number pregnancies (% female)	0 13.25% (48)<br>1 12.15% (44)<br>2 33.98% (123)<br>3 19.89% (72)<br>4 8.56% (31)<br>≥5 11.6% (42)<br>Not known 0.55% (2)	0 7.14% (6)<br>1 10.7% (9)<br>2 47.6% (40)<br>3 21.4% (18)<br>4 8.33% (7)<br>5 1.19% (1)<br>Not known 3.57% (3)
SCAD recurrence (%, n); *variable follow up durations	No recurrence 88.54% (340)<br>Single 8.85% (34)<br>Two or more 1.82% (7)<br>Not known 0.78% (3)	No recurrence 89.13% (82)<br>Single 7.61% (7)<br>Two or more 3.26% (3)
Remote arteriopathies* (%, n)	Fully screened and no arteriopathy 31.25% (120)<br>Arteriopathy in any vascular bed 29.17% (112)<br>Incomplete screening with no known arteriopathy 39.58% (152)	Arteriopathy in any vascular bed 14% (13)<br>Unknown / no arteriopathy 86% (79)
Hypertension (%, n)	Hypertensive 24.5% (94)<br>Not hypertensive 75.5% (290)	Hypertensive 7.61% (7)<br>Not hypertensive 92.4% (85)

American Heart Association.
Circulation: Genomic and Precision Medicine

This article is published in its accepted form; it has not been copyedited and has not appeared in an issue of the journal. Preparation for inclusion in an issue of *Circulation: Genomic and Precision Medicine* involves copyediting, typesetting, proofreading, and author review, which may lead to differences between this accepted version of the manuscript and the final, published version.

DOI: 10.1161/CIRCGEN.120.003030

Pregnancy-related SCAD (% female, n)	P-SCAD 8.8% (32)<br>Non P-SCAD 91.1% (330)	P-SCAD 10.71% (9)<br>Non P-SCAD 85.7% (72)
Smoker (%, n)	Past 27.08% (104)<br>Current 3.65% (14)<br>Never 66.93% (257))<br>Unknown 2.34% (9)	Past 32.6% (30)<br>Current at SCAD 3.26% (3)<br>Never 60.9% (56)<br>Unknown 3.26% (3)
Type 2 diabetes (%, n)	No diabetes 98.18% (377)<br>Diabetes 1.82% (7)	No diabetes 96.7% (89)<br>Diabetes 3.26% (3)

*****Arteriopathy is defined as any arterial abnormality and may include dilations of arteries outside normal limits, dissections, aneurysms and fibromuscular dysplasia but does not include arterial tortuosity. CTD – connective tissue disorder; PCKD – polycystic kidney disease**.** P-SCAD is defined as SCAD occurring during pregnancy or within 12 months of delivery. Note for UK data number of pregnancies is the number of gestations, for Australian data this is the number of live births.

American Heart Association.

Circulation: Genomic and Precision Medicine

This article is published in its accepted form; it has not been copyedited and has not appeared in an issue of the journal. Preparation for inclusion in an issue of *Circulation: Genomic and Precision Medicine* involves copyediting, typesetting, proofreading, and author review, which may lead to differences between this accepted version of the manuscript and the final, published version.

Table 2. Pathogenic and likely pathogenic variants identified in 384 SCAD cases

Gene	Tier	Variant (GRCh38)	Transcript	Transcript codon change	Protein change	Variant type	GT	Sample(s)	GnomAD exome global AF	GnomAD exome popmax AF	Previously reported as P/LP	Variant class
COL3A1	1	2-189011668-G-T	ENST00000304636	c.4295G>T	p.Arg1432Leu	Missense variant	het	BPt00521469	2.0E-5	3.6E-5	Yes (32)	LP
COL3A1	1	2-188990117-C-T	ENST00000304636	c.712C>T	p.Arg238*	Stop gained	het	ScPt0162409J	0	0	Yes (33)	P
PKD1	1	16-2106443-CCA-C	ENST00000262304	c.7442_7443del TG	p.Leu2481fs	Frameshift variant	het	ScPt0150875X	0	0	No	P
PKD1	1	16-2090692-G-C	ENST00000262304	c.12120C>G	p.Tyr4040*	Stop gained	het	ScPt0395467Z	0	0	No	P
PKD1	1	16-2109337-C-T	ENST00000262304	c.5830G>A	p.Gly1944Arg	Missense variant	het	ScPt0743044L	1.8E-4	2.9E-4	Yes (34)	LP
PKD1	1	16-2118102-G-C	ENST00000262304	c.890C>G	p.Pro297Arg	Missense variant	het	ScPt0899224S	3.7E-5	8.3E-5	Yes (35)	LP
PKD1	1	16-2106665-G-A	ENST00000262304	c.7222C>T	p.Arg2408Cys	Missense variant	het	ScPt0932588J	8.5E-5	1.2E-4	Yes (36)	LP
SMAD3	1	15-67066155-A-T	ENST00000327367	c.1A>T	p.Met1?	Initiator codon variant	het	ScPt0115454T	0	0	Yes (37)	LP
SMAD3	1	15-67190432-A-AC	ENST00000327367	c.1179dupC	p.Cys394fs	Frameshift variant	het	ScPt0475518M	0	0	Yes (38)	LP
*LOX**	2	5-122074155-A-C	ENST00000231004	c.893T>G	p.Met298Arg	Missense variant	het	BPt00003870	0	0	Yes (39)	LP
MYLK	2	3-123708719-G-A	ENST00000346322	c.1912C>T	p.Gln638*	Stop gained	het	ScPt0419597W	4.0E-6	9.0E-6	Yes (33)	P
TGFB2	2	1-218436110-C-T	ENST00000366929	c.979C>T	p.Arg327Trp	Missense variant	het	ScPT0443400X	0	0	Yes (22)	LP
*TGFB2**	2	1-218434118-C-T	ENST00000366929	c.631C>T	p.Arg211Cys	Missense variant	het	ScPt0698633H	0	0	Yes (33)	LP
YY1AP1	2	1-155660577-T-A	ENST00000295566	c.1471A>T	p.Lys491*	Stop gained	het	ScPt0059010C	0	0	No	P
YY1AP1	2	1-155672733-T-G	ENST00000295566	c.610-2A>C	NA	Splice acceptor variant	het	ScPt0059010C	8.0E-6	1.8E-5	No	P

LP = likely-pathogenic; P = pathogenic; * variant previously reported. [13]

This article is published in its accepted form; it has not been copyedited and has not appeared in an issue of the journal. Preparation for inclusion in an issue of *Circulation: Genomic and Precision Medicine* involves copyediting, typesetting, proofreading, and author review, which may lead to differences between this accepted version of the manuscript and the final, published version.

Table 3. Eleven different genetic models used to define qualifying variants for collapsing analysis

**Model**	**Genetic model**	**External MAF (GnomAD)**	**Variant type**	**Missense restricted to intolerant sub-regions**
PTV	Dominant	0.001	PTVs	NA
PTV or rare damaging	Dominant	0.001 (PTVs)<br>0.00005 (non-PTVs)	PTVs or non-PTVs REVEL Score >=0.25	No
Ultra-rare damaging	Dominant	0	REVEL Score >=0.25	No
Ultra-rare damaging (MTR)	Dominant	0	REVEL Score >=0.25	Yes
Rare damaging	Dominant	0.00005	REVEL Score >=0.25	No
Rare damaging (MTR)	Dominant	0.00005	REVEL Score >=0.25	Yes
Flexible damaging	Dominant	0.0005 (popmax 0.001)	REVEL Score >=0.25	No
Flexible non-syn	Dominant	0.0005 (popmax 0.001)	All non-synonymous	No
Flexible non-syn (MTR)	Dominant	0.0005 (popmax 0.001)	All non-synonymous	Yes
Recessive	Recessive	0.005	All non-synonymous	No
Synonymous (negative control)	Dominant	0.00005	Synonymous	NA

PTV=protein-truncating variant; MTR=missense tolerance ratio; REVEL=score to predict damage caused by variant; MAF=minor allele frequency; popmax=maximum MAF across the different gnomAD populations. For full details of qualifying variant definitions see Supplementary Methods.

Circulation: Genomic and Precision Medicine

This article is published in its accepted form; it has not been copyedited and has not appeared in an issue of the journal. Preparation for inclusion in an issue of *Circulation: Genomic and Precision Medicine* involves copyediting, typesetting, proofreading, and author review, which may lead to differences between this accepted version of the manuscript and the final, published version.

DOI: 10.1161/CIRCGEN.120.003030

Table 4: Selected highly ranked collapsing analysis results

Gene Name	Gene Description	Model	Qual Cases	Qual Case PC	Qual Ctrls	Qual Ctrl PC	P-value	Odds Ratio	Odds Ratio LCI	Odds Ratio UCI
PKD1	Polycystin-1. Mutated in PCKD and previously implicated in SCAD.	Ultra-rare damaging (MTR)	6	1.7%	14	0.1%	7.31E-06	16.7	6.4	43.8
TBC1D9	TBC domain family member 9.	Rare damaging (MTR)	7	2%	31	0.2%	4.07E-05	8.8	3.9	20.2
TCEAL7	Transcription elongation factor A-like 7.	Flexible non-syn	4	1.1%	5	0%	4.63E-05	31.1	8.3	116.3
DENND5A	DENN domain containing 5 A. RAB guanine nucleotide exchange factor.	Flexible non-syn	15	4.2%	167	1.2%	6.24E-05	3.6	2.1	6.1
ERC1	ELKS/RAB-6-interacting/CAST family member 1. Regulatory subunit of IKK complex.	Flexible damaging	10	2.8%	78	0.6%	7.55E-05	5	2.6	9.8
CHRNA7	Cholinergic receptor nicotinic alpha 7 subunit. Ligand-gated ion channel.	Flexible damaging	4	1.1%	6	0%	7.56E-05	25.9	7.3	92.2
PAM	Peptidylglycine alpha-amidating monooxygenase. Enzyme involved in biosynthesis of neural and endocrine peptides.	Flexible damaging	9	2.5%	73	0.5%	2.25E-04	4.8	2.4	9.7
GLI3	GLI family zinc finger 3. Transcriptional effector of hedgehog signalling.	PTV	2	0.6%	0	0%	6.41E-04	NA	NA	NA
NFATC4	Nuclear factor of activated T cells 4. Transcription factor invoved in numerous processes including cardiovascular development.	PTV	2	0.6%	0	0%	6.41E-04	NA	NA	NA
SEC24B	SEC24 homolog B, COPII coat complex component. Involved in vesicle trafficking.	Ultra-rare damaging	4	1.1%	17	0.1%	0.0017	9.1	3.1	27.3

American Heart Association.
Circulation: Genomic and Precision Medicine

This article is published in its accepted form; it has not been copyedited and has not appeared in an issue of the journal. Preparation for inclusion in an issue of *Circulation: Genomic and Precision Medicine* involves copyediting, typesetting, proofreading, and author review, which may lead to differences between this accepted version of the manuscript and the final, published version.

HDAC9	Histone deacetylase 9. Deacetylases lysine residues of histones. Common variants associated with stroke and CAD.	Flexible damaging	4	1.1%	22	0.2%	0.0039	7.1	2.4	20.6
COL18A1	Collagen type XVIII alpha 1 chain. Endostatin antiangiogenic protein.	Ultra-rare damaging	6	1.7%	56	0.4%	0.0047	4.2	1.8	9.7
ARNTL	Aryl hydrocarbon receptor nuclear translocator like. Transcriptional component of circadian clock.	Ultra-rare damaging (MTR)	2	0.6%	3	0%	0.0061	25.8	4.3	154.7
TBX2	T-Box transcription factor 2. Involved in heart development.	Flexible damaging	7	2%	82	0.6%	0.0074	3.3	1.5	7.3
SOX9	SRY-Box transcription factor 9. Involved in skeletal development.	Ultra-rare damaging	3	0.8%	14	0.1%	0.0084	8.3	2.4	29
SORBS2	Sorbin And SH3 domain containing 2. Adaptor protein involved in regulation of cell adhesion, cytoskeleton and growth factor signalling.	Ultra-rare damaging	3	0.8%	15	0.1%	0.0099	7.7	2.2	26.9
COL4A2	Collagen type IV alpha 2 chain. Component of endothelial basement membrane.	Ultra-rare damaging	5	1.4%	47	0.3%	0.01	4.1	1.6	10.5

Collapsing analysis associations with p<1e-4 (excluding flagged genes, see Supplementary Table 6), plus those that are highly ranked and have been prioritized by manual or machine learning approaches. Only the model with the lowest p-value is shown for each gene. For full results see Supplementary Table 6. LCI=Lower confidence interval (95%), UCI=Upper confidence interval (95%).

Circulation: Genomic and Precision Medicine

American Heart Association.

This article is published in its accepted form; it has not been copyedited and has not appeared in an issue of the journal. Preparation for inclusion in an issue of *Circulation: Genomic and Precision Medicine* involves copyediting, typesetting, proofreading, and author review, which may lead to differences between this accepted version of the manuscript and the final, published version.
